# Inductive Production of the Iron-Chelating 2-Pyridones Benefits the Producing Fungus To Compete for Diverse Niches

**DOI:** 10.1128/mbio.03279-21

**Published:** 2021-12-14

**Authors:** Bo Chen, Yanlei Sun, Shiqin Li, Ying Yin, Chengshu Wang

**Affiliations:** a Key Laboratory of Insect Developmental and Evolutionary Biology, CAS Center for Excellence in Molecular Plant Sciences, Shanghai Institute of Plant Physiology and Ecology, Chinese Academy of Sciences, Shanghai, China; b CAS Center for Excellence in Biotic Interactions, University of Chinese Academy of Sciences, Beijing, China; c School of Life Science and Technology, ShanghaiTech University, Shanghai, China; Cornell University

**Keywords:** 2-pyridone, tenellin, biosynthetic regulation, methylglucosylation, iron chelation, niche competition

## Abstract

Diverse 2-pyridone alkaloids have been identified with an array of biological and pharmaceutical activities, including the development of drugs. However, the biosynthetic regulation and chemical ecology of 2-pyridones remain largely elusive. Here, we report the inductive activation of the silent polyketide synthase-nonribosomal peptide synthetase (PKS-NRPS) (*tenS*) gene cluster for the biosynthesis of the tenellin-type 2-pyridones in the insect-pathogenic fungus Beauveria bassiana when cocultured with its natural competitor fungus Metarhizium robertsii. A pathway-specific transcription factor, *tenR*, was identified, and the overexpression of *tenR* well expanded the biosynthetic mechanism of 15-hydroxytenellin (15-HT) and its derivatives. In particular, a tandemly linked glycosyltransferase-methyltransferase gene pair located outside the *tenS* gene cluster was verified to mediate the rare and site-specific methylglucosylation of 15-HT at its N-OH residue. It was evident that both tenellin and 15-HT can chelate iron, which could benefit B. bassiana to outcompete M. robertsii in cocultures and to adapt to iron-replete and -depleted conditions. Relative to the wild-type strain, the deletion of *tenS* had no obvious negative effect on fungal virulence, but the overexpression of *tenR* could substantially increase fungal pathogenicity toward insect hosts. The results of this study well advance the understanding of the biosynthetic machinery and chemical ecology of 2-pyridones.

## INTRODUCTION

**FIG 1:**
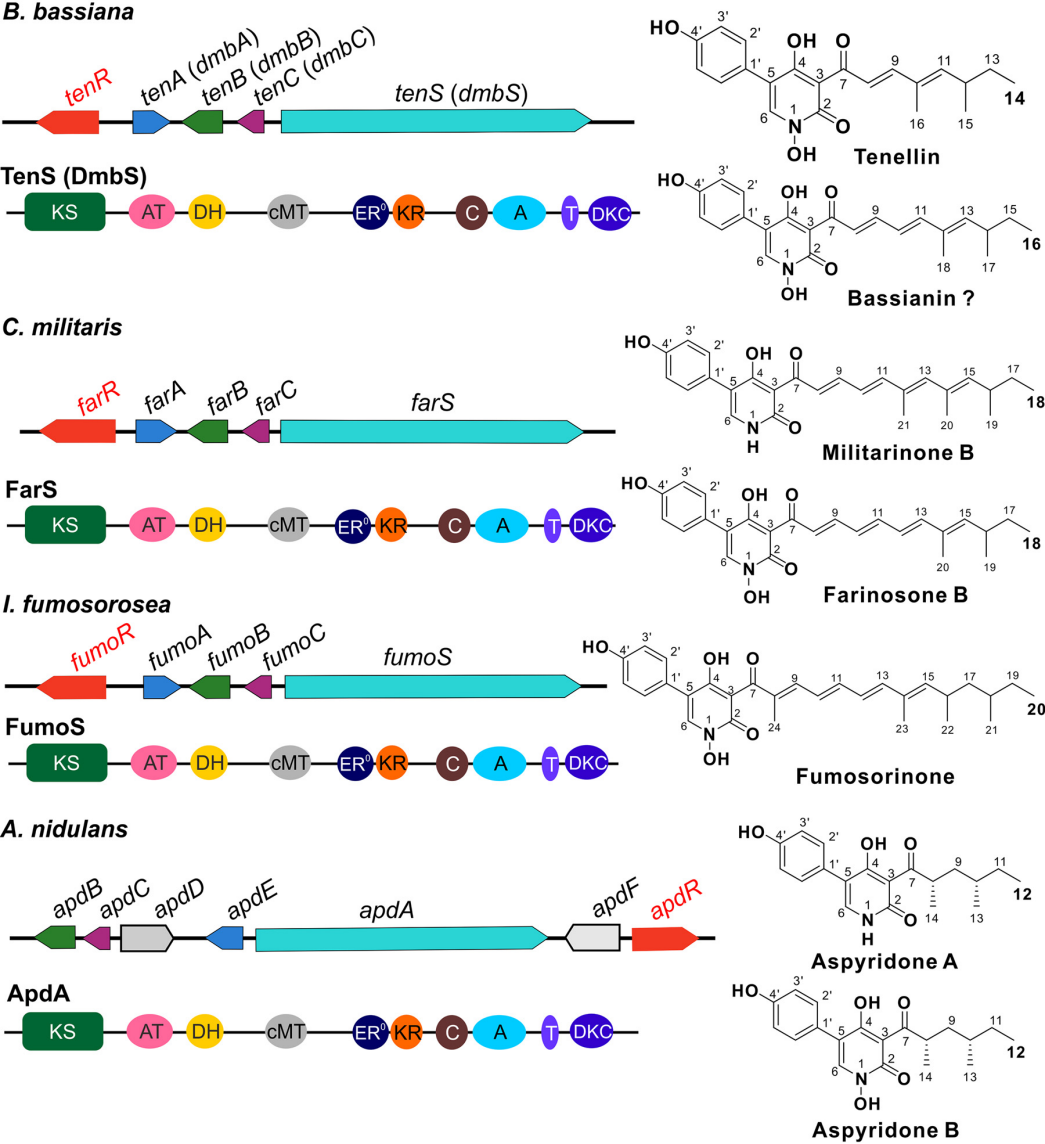
Structuring of the conserved gene clusters and key PKS-NRPS enzymes involved in the biosynthesis of analogous 2-pyridones in different fungi. The genes labeled in the same color show orthologous relationships with each other. The *tenA* and *tenB* homologous genes encode two cytochrome P450 enzymes, *tenC* homologues encode the putative enoyl reductases, *tenS* homologues encode the hybrid PKS-NRPS enzymes, and *tenR* homologues each encode a pathway-specific transcription factor. The domains within the PKS-NRPS hybrid enzyme are indicated. KS, β-ketosynthase; AT, acyl transferase; DH, dehydratase; cMT, C-methyltransferase; ER^0^, nonfunctional enoyl reductase; KR, ketoreductase; C, condensation domain; A, adenylation domain; T, thiolation domain; DKC, Dieckmann cyclase. The production of bassianin by B. bassiana remains questioned.

The chemical ecology of secondary metabolisms (SMs) has received considerable attention (1, 2). The bioactive metabolites with antibiotic activities are implicated in microbial interactions to render either one-sided or dual-inhibition effects (3, 4). Different metabolites have also been confirmed to contribute to the full virulence of both plant- and insect-pathogenic fungi (5–7). Otherwise, different microbes have evolved with the abilities to produce unlike types (e.g., catecholate, hydroxamate, phenolate, and carboxylate types) of extracellular and/or intracellular siderophores for iron sequestration, uptake, transport, storage, or detoxification that may contribute to microbial interactions with different environments, including hosts (8, 9). The hydroxamate-type siderophores are mainly produced by different fungi (8, 10). The *N*-hydroxy-type 2-pyridones contain the hydroxamate moieties (Fig. 1). Except for the 2-pyridones leporin B produced by Aspergillus flavus and tenellin produced by Beauveria bassiana (11, 12), the iron-chelating activity and biological function of 2-pyridones remain elusive in filamentous fungi.

A plethora of 2-pyridones have been identified from different organisms with antimicrobial, antitumor, neurotrophic, and/or insecticidal activities, and quite a few drugs have been developed from these alkaloids (13). Filamentous fungi can produce different structures of 2-pyridones. For example, entomopathogenic fungi like *Beauveria* and *Cordyceps* species produce analogous 2-pyridones such as the tenellin (14), bassianin (14), farinosones (15), militarinones (16, 17), and fumosorinone (18), with variations in the lengths and methylation degrees of the side chains (Fig. 1). Conserved polyketide synthase-nonribosomal peptide synthetase (PKS-NRPS) hybrid gene clusters have been verified in different fungi for the biosynthesis of diverse 2-pyridones and their derivatives such as tenellin (19, 20), (desmethyl)bassianin (21), aspyridones (22), harzianopyridones (23), and ilicicolins (24). The highly reducing PKS region of these core hybrid enzymes contains a nonfunctional enoyl reductase (ER) domain, and the full function of PKS-NRPS requires an ER enzyme encoded by a separate gene within each cluster (Fig. 1). For example, the PKS-NRPS TenS and the ER TenC work together to initiate the biosynthesis of tenellin in B. bassiana (19, 20), and Aspergillus ApdA and ApdC jointly biosynthesize the initial intermediates for the production of aspyridones (22). Otherwise, two divergent cytochrome P450 (CYP) enzymes are encoded by each cluster to mediate the tetramate ring expansion and hydroxylation of intermediates into the *N-*hydroxy type of 2-pyridones (Fig. 1). In particular, a methylglucoside-type derivative of tenellin has also been identified from B. bassiana (25). The production of this compound would require the function of methyltransferase (MT) and glycosyltransferase (GT) that are absent from the PKS-NRPS gene cluster (Fig. 1; see also Table S1 in the supplemental material). The biosynthetic mechanism of 2-pyridones thus requires further elucidation, including the regulation of 2-pyridone production.

10.1128/mbio.03279-21.7 TABLE S1 Conserved gene clusters involved in the biosynthesis of 2-pyridones in different fungi. Download Table S1, PDF file, 0.5 MB. Copyright © 2021 Chen et al. 2021 Chen et al. https://creativecommons.org/licenses/by/4.0/ This content is distributed under the terms of the Creative Commons Attribution 4.0 International license.

It is common that the SM gene clusters of different fungi remain silent under laboratory growth conditions, such as the tenellin biosynthetic genes (26). Different strategies such as the activation of the global regulator and pathway-specific transcription factor (TF) and the use of epigenetic modifiers have proven successful for the induction of metabolite production by fungi (27). Otherwise, microbial coculturing could induce the production of novel metabolites (3, 28). This process can somehow mimic the environmental conditions of microbial interactions. Different insect-pathogenic fungi such as the *Beauveria* and Metarhizium species are omnipresent and coexistent in different environments and microniches (29, 30). We have found that B. bassiana is inferior to compete for insect individuals with Metarhizium robertsii but could outcompete the latter when the two fungi were cocultured in artificial media (31). The mechanism(s) of this kind of antagonistic effect remains unclear.

Here, we report that the production of tenellin 2-pyridones was induced in B. bassiana to outcompete the nonproducer M. robertsii in cocultures by iron sequestration. It was verified that the 2-pyridone biosynthetic gene cluster is controlled by a pathway-specific transcription factor, and metabolite methylglucosylation occurs with the function of the genes located outside the gene cluster. The activation of this cluster could also benefit the producing fungus to tolerate iron stresses and infect insect hosts.

## RESULTS

### Production of the tenellin derivatives by B. bassiana in cocultures.

**FIG 2:**
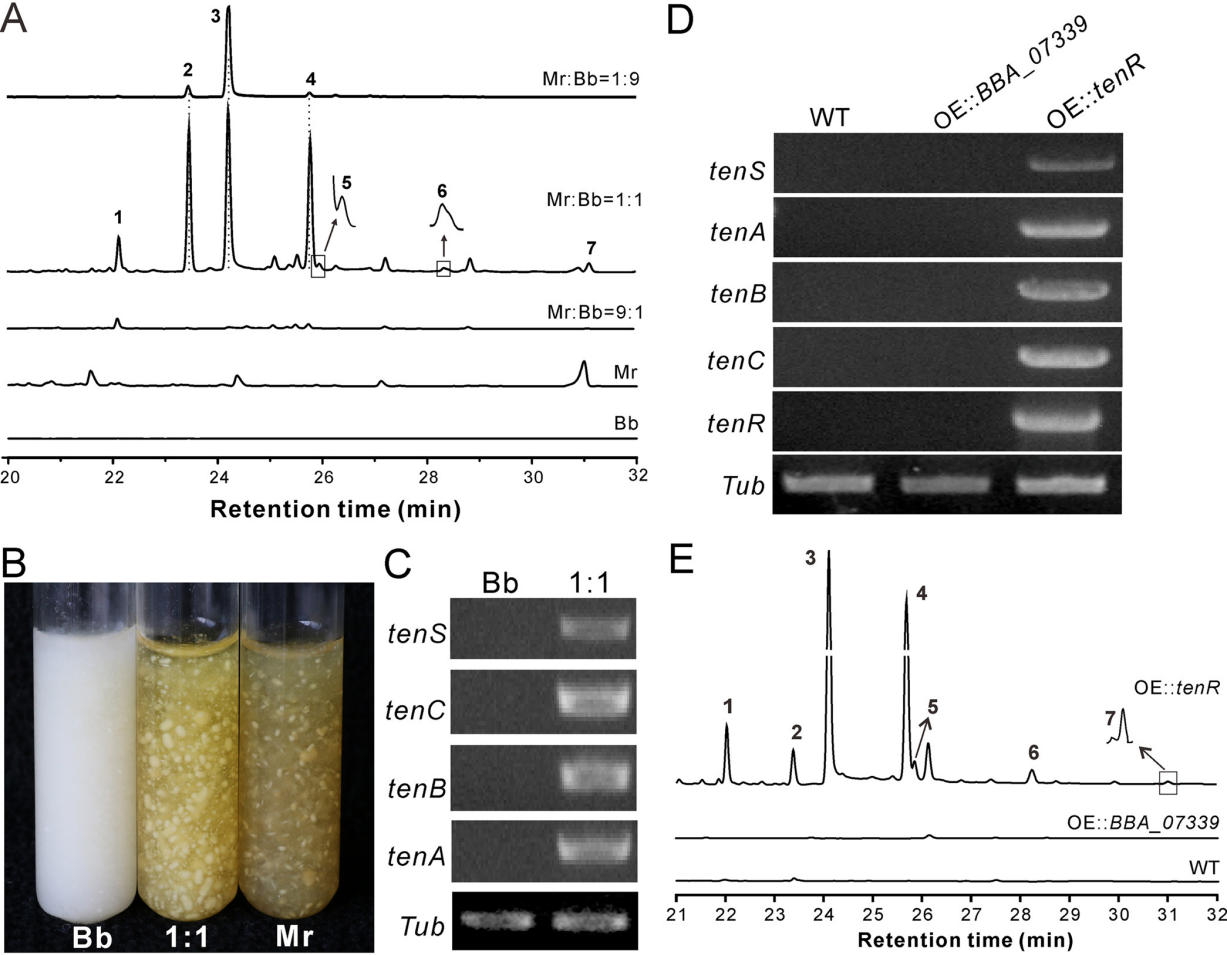
Inductive production of 2-pyridones. (A) HPLC profiles showing the production of the compound peaks in different samples. Spores of *M. robertsii* (Mr), *B.* bassiana (Bb), and their mixtures at different ratios were inoculated into SDB for 9 days before metabolite extraction and profiling. (B) Phenotype of fungal (co)cultures. Spores of *B.* bassiana, *M. robertsii*, and their mixture (1:1) were inoculated into SDB for 9 days. (C) Upregulation of the *tenS* cluster genes in coculture (*B.* bassiana-*M. robertsii* at a 1:1 ratio). *Tub*, β-tubulin gene used as a reference. (D) Upregulation of the clustered genes by the overexpression of *tenR* but not the other putative transcription factor (*BBA_07399*). (E) HPLC analysis showing the production of compounds 1 to 7 by the overexpression of *tenR*. All cultures were grown in SDB for 9 days prior to metabolite extractions.

After coculturing B. bassiana and *M. robertsii* in Sabouraud dextrose broth (SDB), it was found that three peaks were detected in the *M. robertsii*-*B.* bassiana 1:9 cocultures, while seven peaks appeared in the *M. robertsii*-*B.* bassiana 1:1 cocultures compared with that of each pure culture by HPLC (high-performance liquid chromatography) analysis. Similar to the pure culture of either *M. robertsii* or *B.* bassiana, no obvious peak was detected in the *M. robertsii*-*B.* bassiana 9:1 cocultures (Fig. 2A). The phenotype of the 1:1 cocultures was pigmented, which was similar to that of *M. robertsii* instead of *B.* bassiana (Fig. 2B). The 1:1 coculture was then fermented to a large volume for compound purifications. After one-dimensional (1D) and/or two-dimensional (2D) spectrum analyses of the purified compounds (see Data Sets S1 and S2 in the supplemental material), chemicals 1 to 7 were identified as the tenellin-related 2-pyridones (Fig. S1), of which compound 1 [pyridovericin-*N-O*-(4-*O*-methyl-β-d-glucopyranoside) (PMGP)], compound 2 (pyridovericin), compound 3 (15-hydroxytenellin [15-HT]), and compound 7 (tenellin) are the known metabolites that have been identified previously from B. bassiana (20, 25, 32). Compound 4 (1-*O*-methyl-15-HT), compound 5 [(8*Z*)-1-*O*-methyl-15-HT], and compound 6 (termed *O*-methyltenellin A) are novel 2-pyridones associated with tenellin or 15-HT. The production of these compounds indicated that coculturing of *B.* bassiana and *M. robertsii* could induce the former to produce the tenellin-related 2-pyridones. Our reverse transcription (RT)-PCR analysis confirmed that the biosynthetic genes were upregulated by the cocultured *B.* bassiana mycelia but not by the pure *B.* bassiana cultures (Fig. 2C).

10.1128/mbio.03279-21.9 DATA SET S1 1D and 2D NMR data of the compounds identified in this study. Download Data Set S1, PDF file, 0.6 MB. Copyright © 2021 Chen et al. 2021 Chen et al. https://creativecommons.org/licenses/by/4.0/ This content is distributed under the terms of the Creative Commons Attribution 4.0 International license.

10.1128/mbio.03279-21.10 DATA SET S2 1D and 2D NMR spectrum data of the compounds identified in this study. Download Data Set S2, PDF file, 3.9 MB. Copyright © 2021 Chen et al. 2021 Chen et al. https://creativecommons.org/licenses/by/4.0/ This content is distributed under the terms of the Creative Commons Attribution 4.0 International license.

10.1128/mbio.03279-21.1 FIG S1 Structure elucidation of the tenellin-related compounds identified in this study. The compounds labeled in blue are known products reported previously, while those labeled in red are novel chemicals identified in this study. Download FIG S1, TIF file, 2.7 MB. Copyright © 2021 Chen et al. 2021 Chen et al. https://creativecommons.org/licenses/by/4.0/ This content is distributed under the terms of the Creative Commons Attribution 4.0 International license.

### Identification of the pathway-specific transcription factor.

Consistent with the structural similarity of the 2-pyridones produced by different fungi (Fig. 1), the conservative PKS-NRPS gene cluster is present in the genomes of different fungi, including Beauveria brongniartii, Cordyceps militaris, Isaria fumosorosea, and Aspergillus nidulans (Table S1). Phylogenetic analysis of the core PKS-NRPS domains indicated that the ketosynthase (KS) and ketoreductase (KR) domain trees are congruent with each other, and the phylogenetic relationship demonstrated an association with the compound side chain length (Fig. S2). With the obtained genome information for B. bassiana (33), we next found that two putative TF genes, i.e., *BBA_07334* and *BBA_07339* (21% identity with each other at the amino acid level), are closely located to the characterized *tenS* cluster (19, 20). To test the possibility of pathway-specific control by either TF, we overexpressed either gene in a wild-type (WT) strain of B. bassiana. The follow-up RT-PCR analysis revealed that the overexpression of *BBA_07334* but not *BBA_07339* could upregulate the clustered genes in *B.* bassiana when grown solely in SDB (Fig. 2D). Consistently, HPLC profiling detected compounds 1 to 7 in the mutant culture overexpressing the *BBA_07334* gene, whereas the metabolites were not produced by the WT and *BBA_07339* transgenic strains (Fig. 2E). We thus identified the pathway-specific TF gene *BBA_07334*, termed *tenR*. This *tenR*-like gene is also conservatively present in other fungi (Fig. 1; Table S1). To further verify its function, we overexpressed *tenR* in a WT strain of C. militaris, a close relative of *B.* bassiana also containing the conserved PKS-NRPS (*farS*) gene cluster (Table S1). As a result, we found that the cluster genes could be activated, and a sharp peak was produced in the pigmented mutant culture (Fig. S3A to C). The compound was identified to be the 2-pyridone farinosone B (Fig. S3D and Data Sets S1 and S2).

10.1128/mbio.03279-21.2 FIG S2 Phylogenetic analysis of the core PKS-NRPS biosynthetic enzyme domains. (A) Phylogenetic relationship of different enzymes based on the ketosynthase (KS) domain sequences. (B) Phylogenetic relationship of different enzymes based on the ketoreductase (KR) domain sequences. The PKS-NRPS enzymes are TenS (XP_008600657) from B. bassiana strain ARSEF 2860 (used in this study), GenBank accession number CAL69597 from B. bassiana strain CBS 110.25, GenBank accession number PQK13186 from B. bassiana ARSEF 8028, DmbS (GenBank accession number ADN43685) from B. bassiana strain 992.05, the putative conserved PKS-NRPS OAA40384 from *B. brongniartii* strain RCEF 3172, FarS (XP_006673463) from *C. militaris* strain Cm01, GenBank accession number ATY66088 from *C. militaris* strain ATCC 34164, FumoS (XP_018700480) from *Isaria fumosorosea* strain ARSEF 2679, and ApdA (Q5ATG8) from A. nidulans strain A4. B. bassiana strain ARSEF 2860 used in this study is highlighted in boldface type. Download FIG S2, TIF file, 0.5 MB. Copyright © 2021 Chen et al. 2021 Chen et al. https://creativecommons.org/licenses/by/4.0/ This content is distributed under the terms of the Creative Commons Attribution 4.0 International license.

10.1128/mbio.03279-21.3 FIG S3 Production and conversion assays of the 2-pyridone farinosone B. (A) Activation of the *farS* gene cluster by the overexpression of *tenR* in *C. militaris*. *Tub*, β-tubulin gene used as an RT-PCR reference. (B) Phenotyping of the liquid cultures. (C) HPLC profiling of the culture filtrates. (D) Farinosone B structure. (E) Yeast feeding assay showing the unconversion of farinosone B by BbGT1/MT1. Farinosone B was added to the transgenic yeast culture (OD_600_ = 0.6) at a final concentration of 10 μg/ml for 2 days. Download FIG S3, TIF file, 1.9 MB. Copyright © 2021 Chen et al. 2021 Chen et al. https://creativecommons.org/licenses/by/4.0/ This content is distributed under the terms of the Creative Commons Attribution 4.0 International license.

**FIG 3:**
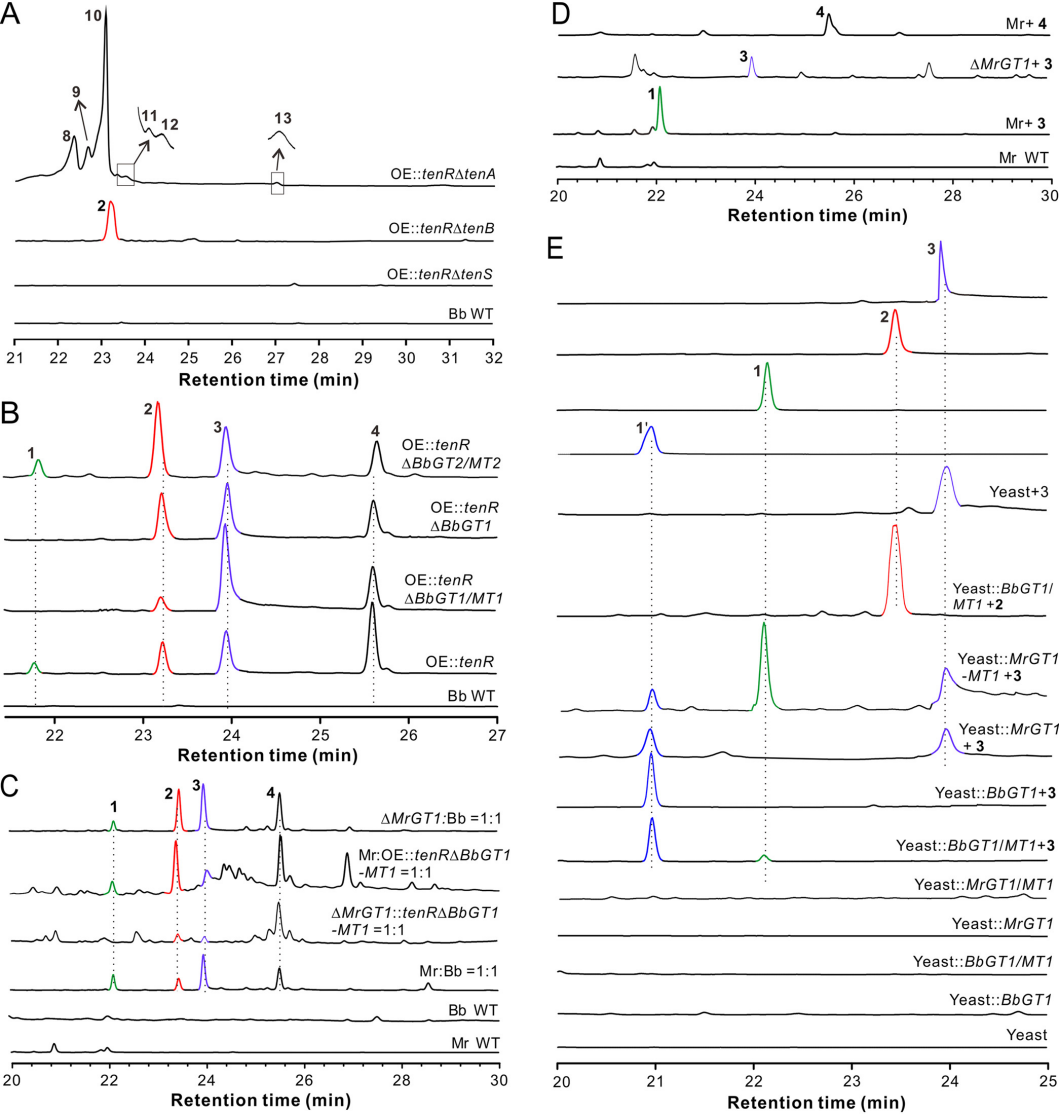
Intermediate production and cross-modification of 15-HT by the nonclustered methylglucosylation genes. (A) HPLC analysis showing the production or nonproduction of different intermediate compounds after gene deletions in the OE::*tenR* mutant of B. bassiana (Bb). (B) Verification of the methylglucosylation genes contributing to the production of compound 1 in B. bassiana. (C) Verification of the cross-modification of 15-HT by *M. robertsii* (Mr). (D) Substrate feeding assay confirming the conversion of compound 3 to compound 1 by *MrGT1*. Feeding with compound 4 could not be converted by *M. robertsii*. (E) Compound conversions by transgenic yeast cells. Compound 2 or 3 was added to the media at a final concentration of 10 μg/ml for 2 days.

We next performed deletions of the core PKS-NRPS gene *tenS* and two CYP genes, *tenA* and *tenB*, in the *tenR* overexpression (OE::*tenR*) strain. Deletion of *tenS* was also conducted in the WT strain for different experiments. After fungal growth in SDB for 9 days, HPLC analysis identified peaks 8 to 13 produced by the OE::*tenR* Δ*tenA* strain, while a single peak was produced by the OE::*tenR* Δ*tenB* strain. Similar to the WT strain grown as a pure culture, no peaks were detected from the OE::*tenR* Δ*tenS* samples (Fig. 3A). The single compound produced by the OE::*tenR* Δ*tenB* strain was identified to be the known compound 2 pyridovericin (32). Peak 8 (12-hydropretenellin A), peak 10 (14-hydropretenellin A), and peak 13 (prototenellin D) were identified as the known compounds reported previously (26), while metabolite 9 (13-hydropretenellin A), metabolite 11 (9-hydropretenellin A), and metabolite 12 (12-oxopretenellin A) are novel chemicals (Fig. S1 and Data Sets S1 and S2).

### Identification of the 4-*O*-methylglucosylation genes outside the gene cluster.

Having found that compound 1, PMGP, is the 4-*O-*methyl glycoside of 15-HT, we were curious about the genes involved in mediating the methylglucosylation of 15-HT. Further examination of the *tenS* cluster did not find any proximal GT and MT genes. We then performed transcriptome sequencing (RNA-seq) analysis of the *B.* bassiana-*M. robertsii* 1:1 coculture together with each pure culture. Not surprisingly, thousands of genes were differentially expressed in cocultures by reference to either the *B.* bassiana or *M. robertsii* pure culture under the same growth conditions (Fig. S4A and B). The data confirmed that the *tenS* cluster genes were substantially upregulated in cocultured *B.* bassiana compared with those expressed by *B.* bassiana alone in SDB (Fig. S4C).

10.1128/mbio.03279-21.4 FIG S4 Differential gene expressions by cocultured B. bassiana (Bb) and *M. robertsii* (Mr). (A) Differential gene expression by *B.* bassiana in coculture (*B.* bassiana-*M. robertsii* at a 1:1 ratio) in reference to its pure culture. (B) Differential gene expression by *M. robertsii* in coculture in reference to its pure culture. FPKM, fragments per kilobase of exon per million reads mapped. (C) Upregulation of the *tenS* cluster genes by *B.* bassiana in coculture compared with the levels expressed by the pure *B.* bassiana culture. (D) Differential expression of the clustered *BbGT/MT* genes by *B.* bassiana in coculture compared with the levels expressed by the pure *B.* bassiana culture. (E) Differential expression of the clustered *MrGT/MT* genes by *M. robertsii* in coculture compared with the levels expressed by the pure *M. robertsii* culture. Fungal cultures were grown in SDB for 9 days prior to RNA extractions for RNA-seq analysis. Download FIG S4, TIF file, 1.5 MB. Copyright © 2021 Chen et al. 2021 Chen et al. https://creativecommons.org/licenses/by/4.0/ This content is distributed under the terms of the Creative Commons Attribution 4.0 International license.

It has been reported that the methylglucosylation of phenolic compounds could be catalyzed by the clustered GT-MT gene pairs of B. bassiana and other fungi (34, 35). Our genome survey found two pairs of clustered GT-MT genes present in the genomes of B. bassiana and *M. robertsii*. In particular, reciprocal BLAST analyses indicated that the pairs *BBA_08686/BBA_08685* (termed *B.* bassiana *GT1/MT1* [*BbGT1/MT1*]) (versus *MAA_06259/MAA_06258* [*M. robertsii GT1/MT1* {*MrGT1/MT1*}]) and *BBA_03583/BBA_03582* (*BbGT2/MT2*) (versus *MAA_00471/MAA_00472* [*MrGT2/MT2*]) are conservatively present in B. bassiana and *M. robertsii* or different fungi other than aspergilli. The transcriptome data indicated that relative to the pure *B.* bassiana culture, the *BbGT1/MT1* but not the *BbGT2/MT2* pair was highly upregulated in cocultures (Fig. S4D). In contrast, the orthologous gene pair *MrGT1/MT1* was substantially downregulated, whereas the *MrGT2/MT2* pair was upregulated in cocultures compared with those of the pure *M. robertsii* sample (Fig. S4E).

To examine the potential contribution of these two gene pairs to the production of the glycoside PMGP, we deleted these two gene pairs in the OE::*tenR* strain. HPLC analysis revealed that the *BbGT1/MT1* but not the *BbGT2/MT2* pair is responsible for PMGP production (Fig. 3B). Not surprisingly, the deletion of *BbGT1* also disabled the production of PMGP by the fungus. We also cocultured *M. robertsii* with the OE::*tenR* Δ*BbGT1/MT1* strain and found evidence of the cross-modification of 15-HT, i.e., the catalysis of 15-HT to PMGP by *M. robertsii* (Fig. 3C). Consistently, PMGP was yielded by direct feeding of the WT strain but not the Δ*MrGT1* strain of *M. robertsii* with 15-HT. However, coculturing of the OE::*tenR* Δ*BbGT1/MT1* and Δ*MrGT1* strains failed to produce detectable PMGP. In addition, it was confirmed that feeding of the Δ*MrGT1* strain with 15-HT or *M. robertsii* with compound 4 (i.e., 1-*O*-methyl-15-HT) did not lead to the occurrence of any conversion (Fig. 3D).

The feeding of transgenic yeast cells further confirmed that 15-HT could be converted to PMGP by either BbGT1/MT1 or MrGT1/MT1. In addition, a novel peak, 1′, appeared in the yeast cultures after feeding with 15-HT (Fig. 3E). This compound was purified and structurally identified as a novel compound, pyridovericin-*N-O*-(β-d-glucopyranoside), i.e., the 4-*O-*position-unmethylated PMGP (Fig. S1 and Data Sets S1 and S2). The feeding of *BbGT1/MT1* transgenic yeast cells with pyridovericin did not show any additional peak (Fig. 3E). Taken together, the results indicated that BbGT1 and MrGT1 target only the N-OH hydroxyl residue of 15-HT. Intriguingly, however, *BbGT1/MT1* transgenic yeasts failed to catalyze the methylglucosylation of farinosone B (Fig. S3E).

### Proposal of the 2-pyridone biosynthetic pathway.

**FIG 4:**
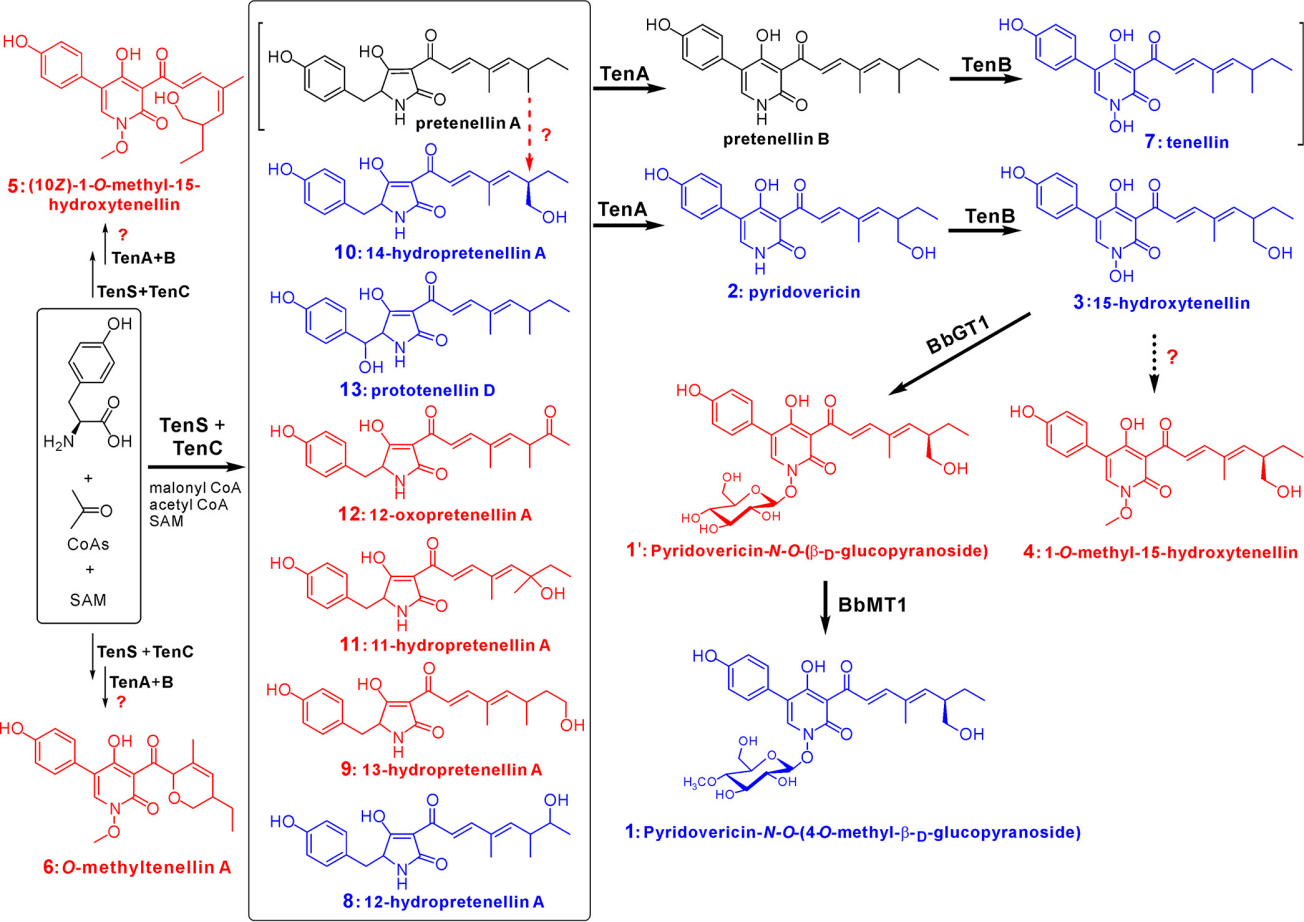
Schematic of the biosynthesis of the tenellin-related compounds. The scheme shown in square brackets for tenellin biosynthesis was suggested previously (20). The question marks indicate that the involved enzymes or pathways remain unclear. The compounds labeled in blue are known products reported previously, while those labeled in red are novel chemicals identified in this study. Pretenellins A and B were not detected in this study. SAM, *S*-adenosylmethionine; CoA, coenzyme A.

Having determined the gene functions and compound structures, we propose the biosynthetic scheme of 15-HT and its derivatives (Fig. 4). By the overexpression of *tenR*, the activated *tenS* and *tenC* complex may be involved in the production of at least six pyrrolidine-2-diones (compounds 8 to 13) with the substrates malonyl-CoA and acetyl-CoA, which was evident from the deletion of *tenA*. The OE::*tenR* Δ*tenB* mutant produced only the compound pyridovericin (compound 2), which would be the product converted by the CYP TenA from compound 10 through the expansion of the tetramate ring, and the CYP TenB would therefore function as an *N-*hydroxylase to mediate the production of 15-HT (compound 3) from pyridovericin. Our data confirmed that the *BbGT1/MT1* genes located outside the *tenS* gene cluster contribute to the stepwise glycosylation and methylation of 15-HT to obtain the glycoside PMGP (Fig. 4). No compound 4 (1-*O-*methyl-15-HT) could be obtained in the 15-HT feedings of *GT1/MT1* transgenic yeast cells (Fig. 3E), which indicated that both *BbMT1* and *MrMT1* are not responsible for the methylation of the N-OH residue of 15-HT to produce chemical 4. The production of compounds 5 and 6 is still elusive, which is involved in the putative processes of oxidative catalysis by either TenA/TenB or an additional oxidase, the Diels-Alder reaction (only for metabolite 6), and the methylation of the N-OH residue catalyzed by an unclear methyltransferase (Fig. 4).

### Biosynthesis of 2-pyridones benefits competitive growth and insect infection of B. bassiana.

**FIG 5:**
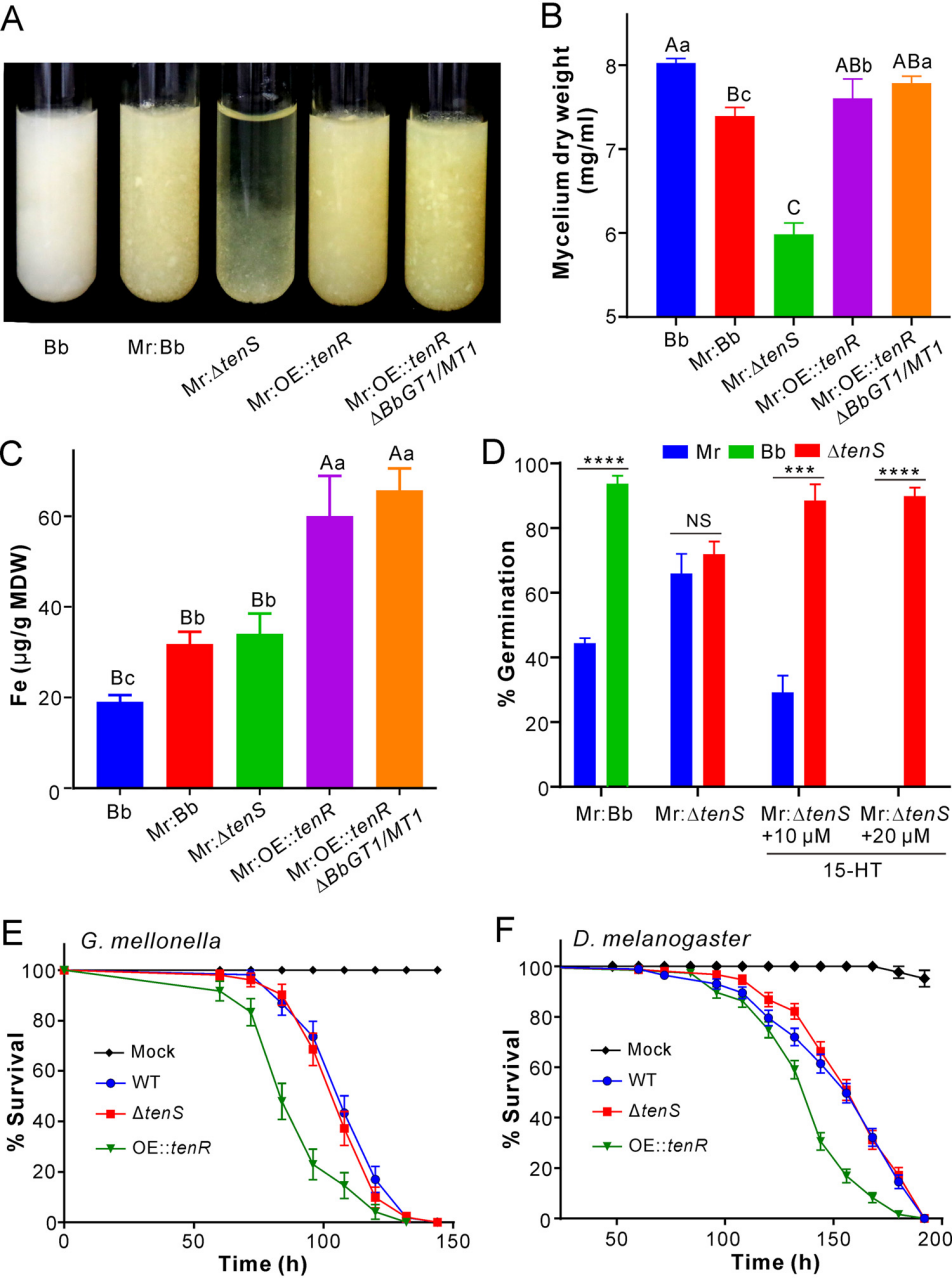
Biological effects of 2-pyridone biosynthesis in B. bassiana. (A) Phenotypes of different B. bassiana (Bb) strains after being cocultured with *M. robertsii* (Mr). (B) Comparison of the biomasses between WT *B.* bassiana and its mutants after being cocultured with *M. robertsii*. (C) Measurement and comparison of the iron contents accumulated in the mycelia of different *B.* bassiana strains after being cultured with *M. robertsii*. In panels A to C, the *M. robertsii* cultures were sealed in dialysis tubing for coculturing. For panels B and C, different letters above each column indicate the level of difference between samples at the level of a *P* value of <0.01 (for uppercase letters) or a *P* value of <0.05 (for lowercase letters) after one-way ANOVA. MDW, mycelium dry weight. (D) Rescue of Δ*tenS* spore germination with 15-HT when the fungus was competed with *M. robertsii*. The spores were mixed at a ratio of 1:1, and the differences were compared between strains germinated under the same conditions by two-tailed Student’s *t* test (***, *P < *0.001; *****, *P < *0.0001; NS, no significant difference). (E) Survival of wax moth larvae after topical infection with different strains. (F) Survival of fruit flies after topical infection with different strains. The mock control was treated with 0.05% Tween 20.

Next, we aimed to know the biological effect of the inductive production of 2-pyridones by B. bassiana. Except for the variation of culture pigmentations, the mycelial biomasses had no obvious difference between the WT and mutants of *B.* bassiana after growing individual strains in SDB (Fig. S5A and B). Further coculturing of *B.* bassiana with the *M. robertsii* mycelia sealed in dialysis tubing revealed that the cocultured *B.* bassiana biomasses were considerably (*P < *0.01) reduced compared with the pure *B.* bassiana culture, i.e., the growth inhibition effect of coculturing (Fig. 5A and B). After the deletion of *tenS*, the mutant biomasses were significantly reduced (*P < *0.01) compared with those of the WT or other mutants. However, the biomasses of the OE::*tenR* and OE::*tenR* Δ*BbGT1/MT1* strains were substantially (*P < *0.05) increased compared with that of *B.* bassiana harvested from the *M. robertsii*-*B.* bassiana cocultures. Thus, the production of 2-pyridones could facilitate *B.* bassiana to counteract the inhibition effect of *M. robertsii* in cocultures.

10.1128/mbio.03279-21.5 FIG S5 Liquid culturing and spore germination assays. (A and B) After growing the WT and mutants of *B. bassiana* in SDB with *M. robertsii*, OE::*tenR* and OE::*tenR* Δ*BbGT1/MT1* cultures turned yellow (A), whereas the mycelial biomass had no obvious difference between the WT and mutants (B). (C) Spore germinations also had no difference when the fungi were germinated in SDB, but the rates were inhibited to different levels with the addition of either FeSO_4_ (3 mM) or phenanthroline (Phen) (20 μM). (D and E) After supplementation with 15-HT (20 μM), the inhibition rates of FeSO_4_ (D) and Phen (E) on the WT and Δ*tenS* strains of *B. bassiana* were significantly increased, but the potentiating effect was not obvious for the OE::*tenR* (except for the Phen plus 15-HT treatment) and OE::*tenR* Δ*BbGT1/MT1* strains. Different letters above each column indicate the level of difference between samples at the level of a *P* value of <0.01 (for uppercase letters) or a *P* value of <0.05 (for lowercase letters) after one-way ANOVA. Download FIG S5, TIF file, 1.3 MB. Copyright © 2021 Chen et al. 2021 Chen et al. https://creativecommons.org/licenses/by/4.0/ This content is distributed under the terms of the Creative Commons Attribution 4.0 International license.

We performed iron chelation tests and found that both tenellin and 15-HT but not methylated 15-HT (i.e., compound 4) could chelate ferric iron (Fig. S6). Iron quantification analysis revealed that coculturing substantially (*P < *0.05) facilitated *B.* bassiana to sequester and take up iron compared with the pure *B.* bassiana culture. In particular, the mycelia of the OE::*tenR* and OE::*tenR* Δ*BbGT1/MT1* strains accumulated a much higher (*P < *0.01) level of iron than those of other strains (Fig. 5C). To test the contribution of 15-HT to fungal competition, we performed spore germination assays in a mixed ratio (1:1) with *M. robertsii* in SDB. It was found that WT *B.* bassiana spores could germinate much faster than those of *M. robertsii* (*P < *0.0001), whereas no significant difference was observed between *M. robertsii* and the Δ*tenS* strain; i.e., the deletion of *tenS* impaired *B.* bassiana spore germination (*P < *0.01) when competing with *M. robertsii*. After the addition of 15-HT, however, the germination rate of Δ*tenS* spores could be rescued to the WT level, while *M. robertsii* spore germination was reduced or completely inhibited (Fig. 5D). Thus, the inductive production of 2-pyridones might facilitate *B.* bassiana to compete for iron to benefit fungal germination and proliferation in cocultures.

10.1128/mbio.03279-21.6 FIG S6 Iron chelation tests. LC-MS analysis confirmed the chelation of ferric irons by tenellin (A and C) and 15-hydroxytenellin (B and C) but not by the compound 1-*O-*methyl-15-hydroxytenellin (D). Download FIG S6, TIF file, 1.5 MB. Copyright © 2021 Chen et al. 2021 Chen et al. https://creativecommons.org/licenses/by/4.0/ This content is distributed under the terms of the Creative Commons Attribution 4.0 International license.

We also performed insect bioassays by topical infection of wax moth larvae and fruit fly females. The results indicated that the overexpression of *tenR* could significantly increase fungal virulence against both caterpillars (χ^2^ = 21.69; *P < *0.001 [by a log rank test]) and fruit flies (χ^2^ = 41.09; *P < *0.001) compared with the WT strain. However, no obvious difference was observed between the WT and Δ*tenS* strains against both insect species (Fig. 5E and F).

### Production of 2-pyridones alleviates fungal iron stress.

**FIG 6:**
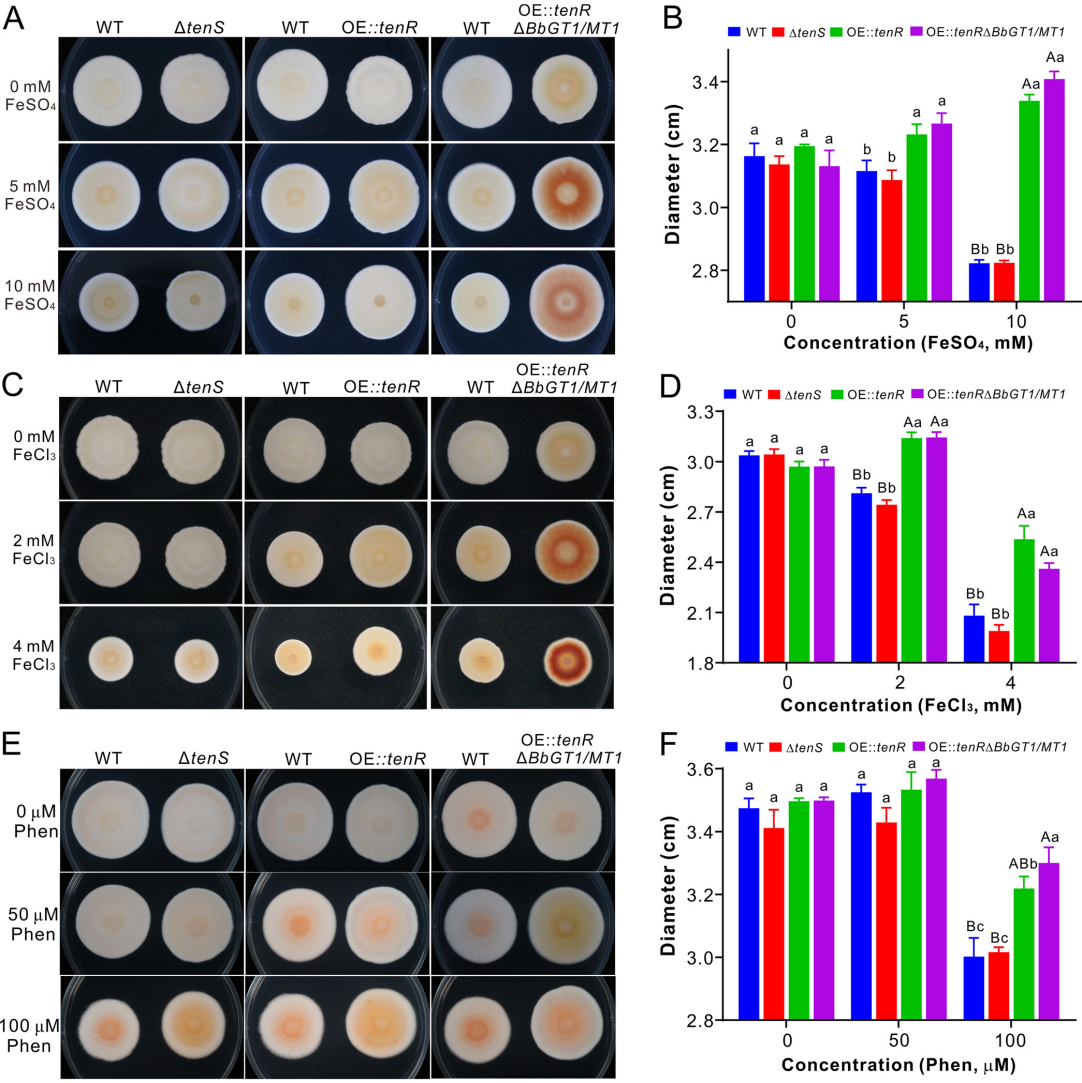
Iron stress responses. (A and B) Phenotyping and comparison of different B. bassiana strains after growth on PDA amended with different concentrations of FeSO_4_ for 15 days. (C and D) Phenotyping and comparison of different strains after growth on PDA amended with different concentrations of FeCl_3_ for 15 days. (E and F) Phenotyping and comparison of different strains after growth on PDA amended with different concentrations of the iron chelator phenanthroline (Phen) for 15 days. Different letters above each column indicate the level of difference between samples at the level of a *P* value of <0.01 (for uppercase letters) or a *P* value of <0.05 (for lowercase letters) after one-way ANOVA.

Iron competition or toxicity can determine the outcome of interspecies interactions and organismal growth (36). By growing the fungi on potato dextrose agar (PDA) and PDA amended with different concentrations of FeSO_4_ or FeCl_3_, obvious toxic effects could be found for the WT and Δ*tenS* strains grown on iron-replete media. In contrast, the OE::*tenR* and OE::*tenR* Δ*BbGT1/MT1* strains grew quicker (*P < *0.05) than the WT and Δ*tenS* strains did on PDA amended with FeSO_4_ or FeCl_3_ (Fig. 6A to D). Relative to the OE::*tenR* strain, the growth of the OE::*tenR* Δ*BbGT1/MT1* strain was substantially increased (*P *= 0.008) on PDA amended with 10 mM FeSO_4_, whereas it was reduced (*P* = 0.046) on the medium amended with 4 mM FeCl_3_. On the other hand, it was found that *B.* bassiana growth could also be inhibited by the addition of the iron chelator phenanthroline (Phen), while *tenR* overexpression mutants could substantially (*P < *0.05) potentiate the inhibition effect compared with the WT and Δ*tenS* strains (Fig. 6E and F). The OE::*tenR* Δ*BbGT1/MT1* strain grew quicker (*P *= 0.022) than the OE::*tenR* strain on PDA amended with 100 μM Phen. Thus, the overexpression of *tenR* could increase fungal abilities to adapt to both iron-replete and -depleted conditions, while the deletion of *BbGT1/MT1* in the OE::*tenR* strain either increased or decreased fungal capacity against a high concentration of iron or an iron chelator agent.

We further performed spore germination assays and found that there was no significant difference between the WT and mutants when the spores had been germinated in SDB. In contrast, the addition of either FeSO_4_ or Phen could significantly inhibit the germination of both WT and mutant spores, of which the Δ*tenS* strain was the most susceptible, followed by the WT, whereas the OE::*tenR* and OE::*tenR* Δ*BbGT1/MT1* mutants were relatively tolerant (Fig. S5C). However, after supplementation with 15-HT, the germination rates of the WT and Δ*tenS* strains were significantly (*P < *0.05) increased compared with sole treatment with either FeSO_4_ or Phen, which could not reach the level of each mock control. In contrast, the addition of this 2-pyridone had no obvious effect to alleviate the inhibition of FeSO_4_ on the germination of OE::*tenR* and OE::*tenR* Δ*BbGT1/MT1* spores (Fig. S5D). However, after the addition of 15-HT, a significant (*P < *0.01) increase of OE::*tenR*, but not OE::*tenR* Δ*BbGT1/MT1*, germination was observed in SDB with Phen (Fig. S5E). Overall, the obtained data support the results of culture inhibition assays (Fig. 6).

## DISCUSSION

In this study, we found that the tenellin-type 2-pyridones were inductively produced by B. bassiana when cocultured with another insect-pathogenic fungus, *M. robertsii*. It was verified that the pathway-specific transcription factor *tenR* controls the *tenS* gene cluster. In contrast to the previous identifications of the intermediates pretenellins A and B for tenellin biosynthesis (20, 26), these two compounds were not detected, and 15-HT instead of tenellin was produced by the B. bassiana strain used in this study. In addition, we did not find the production of the pyridomacrolidin analogues, tenellin, and lactone-conjugated products (26, 37). Apart from the different strains used in previous studies, the alternative growth conditions, including the use of the epigenetic modifier 5-azacytidine, might jointly result in the production of different structural analogues. In this study, nevertheless, six novel compounds were identified. In particular, the methylglucosylated 2-pyridone PMGP was found to be converted from 15-HT by the *BbGT1*/*BbMT1* genes located outside the *tenS* gene cluster. However, the putative methyltransferase involved in the production of the novel metabolite 1-*O-*methyl-15-HT (4) and the Diels-Alderase involved in the formation of the novel compound 6 remain to be determined.

Reclassification and misclassification of fungi, including *Beauveria* species (38), are common. For example, the 2-pyridone bassianin-producing fungus Beauveria tenella has been reclassified as *B. brongniartii* (39). However, the *dmbS* gene cluster was characterized as being highly conserved with the *tenS* cluster in B. bassiana strain 992.05 for desmethylbassianin production (21, 40, 41). We did not find bassianin or desmethylbassianin in this study. Taken together with the results of our phylogenetic analysis and the rule of fungal chemical taxonomy (42), this would suggest that bassianin and its analogues might be produced by a *Beauveria* species other than B. bassiana. We also find that the overexpression of *tenR* in *C. militaris* led to the production of farinosone B, a metabolite that was first isolated from Paecilomyces farinosus (now reclassified as Isaria farinosa) (15). Instead, the mutant did not produce any militarinone-type 2-pyridones, which were previously isolated from Paecilomyces militaris (now reclassified as *C. militaris*) (17). As indicated above, the B. bassiana strain used in this study mainly produced 15-HT instead of tenellin. Thus, the chemodiversity of 2-pyridone biosynthesis can occur at both inter- and intraspecific levels of different fungi. The variation of side chain length among these 2-pyridones is well associated with fungal speciation, which can be an ideal model for future investigation of the mechanism of the polyketide chain length control that has been related to different domains of PKS (43, 44).

A plethora of glycosylated natural products with diverse activities have been isolated from different organisms (45). The common glycosylation patterns of different products can be summarized as the mode of C-X-Glc (where X is O, C, N, or S) (46). It is rare to find in this study that the glycoside PMGP has the glucosyl moiety at the N-OH residue of 15-HT. To our knowledge, the other *N*-*O*-Glc-type glycosides found so far include only trichostatin D identified from Streptomyces violaceusniger (47) and the glycosylated *N-*hydroxy-pipecolic acid found in Arabidopsis thaliana (48). It has been found that BbGT1 (also called BbGT86) could promiscuously convert a large number of polyketides, flavonoids, and naphthalenes into C*-O-*Glc- or C-*N*-Glc-type glycosides by compound feeding of transgenic yeasts (34). In contrast, we did not find the occurrence of (methyl)glucosylation at any C-OH residue of 15-HT (i.e., the hydroxyl sites 4, 4′, and 15) within the authentic host B. bassiana or in our yeast feeding assays. Even Metarhizium species do not contain the *tenS*-like 2-pyridone biosynthetic genes (49); the MrGT1/MrMT1 enzyme pair is also encoded by each species and can convert 15-HT to PMGP. Intriguingly, *BbGT1/BbMT1* transgenic yeast cells failed to catalyze the compound farinosone B. The stereoselectivity and stereospecificity of BbGT1 and its orthologues remain to be determined in the future.

Extracellular siderophores are functionally important for iron sequestration and uptake, while intracellular siderophores contribute to iron storage (8). Consistent with the finding that tenellin can chelate iron (12), we found that the main excreted product, 15-HT, identified in this study could also chelate and sequester iron. We found that the cocultured *B.* bassiana mycelia contained a higher level of iron than the pure *B.* bassiana culture, and the deletion of *tenS* could substantially impair *B.* bassiana competitive germination and growth in the cocultures and spore germination under both iron-replete and -depleted conditions. Thus, the inductive production of the iron-chelating 2-pyridones may be the strategy at least partially employed by *B.* bassiana to outcompete *M. robertsii* in cocultures. This finding unveils a previously unsuspected tactic employed by B. bassiana to maintain coexistence in the environment since the fungus is inferior to compete with *M. robertsii* for insect individuals (31). It can be expected that the production of the iron-chelating 2-pyridones may also benefit the fungus to compete with other microbes. In addition to 2-pyridones, additional hydroxamate-type ferricrocins and the coprogen-type siderophore beauverichelin A can also be produced by B. bassiana (10, 12). Both types of siderophores can also be biosynthesized by *M. robertsii* (50, 51). The balancing control of different siderophores in iron sequestration and fungal competition remains to be determined. The *MrGT1*/*MrMT1* genes of *M. robertsii* can function as an additional approach to neutralize iron competition from competitors, a good example of xenobiotic detoxification.

Similar to previous findings (19), we found no obvious difference between the WT and Δ*tenS* strains during topical infection of two insect species. However, the overexpression of *tenR* could considerably increase the virulence of the mutant compared with the WT strain. Both pathogenic microbes and hosts will compete for iron during infective interactions (52). For example, the extracellular siderophore is required for the full virulence of *M. robertsii*, while the iron-binding transferrins were highly upregulated in insects infected by this pathogenic fungus (50). It has been reported that the 2-pyridone leporin A has an anti-insectan/antifeedant effect (11); the activity remains to be determined for tenellin derivatives that may facilitate fungal conquering of insect hosts beyond iron sequestration.

It is common that iron stress responses may occur in different organisms growing under iron-depleted or -replete conditions, in which case the function of siderophores is also required (12, 53). Consistently, we found that the spore germination of the WT and Δ*tenS* strains was deterred in iron-replete and -depleted media, which could be partially rescued by the addition of 15-HT. Also, *tenR* overexpression could increase the adaptive ability of the mutant strains against both ferric/ferrous iron-replete and -depleted conditions. As indicated above, 15-HT can be modified at the N-OH residue with either a methyl or a methylglucosyl moiety. Both modifications can block the iron chelation ability of 15-HT. The OE::*tenR* Δ*BbGT1/MT1* mutant sequestered a higher level of iron than the OE::*tenR* strain did, which could help explain the more severe effect of cell toxicity or tolerance of this strain after being inoculated into iron-rich or -poor media. Thus, the activation and level of 15-HT modifications can potentiate the ability of the fungus to adapt to different iron conditions. This kind of fine-tuning mechanism remains to be determined in terms of the up- or downregulation control of the nonclustered tailoring enzymes.

In conclusion, we found that fungus-fungus coculturing could activate the silent *tenS* gene cluster in B. bassiana to produce the iron-chelating 2-pyridones to benefit the producing fungus to compete for different niches. The biosynthetic mechanism of tenellin derivatives is greatly expanded with the identification of the pathway-specific regulator and the nonclustered genes involved in the methylglucosylation of 15-HT. The results of this study well advance the biosynthetic machinery and chemical ecology of 2-pyridone alkaloids in fungi.

## MATERIALS AND METHODS

### Fungal strains and maintenance.

The WT strains B. bassiana ARSEF 2860, *M. robertsii* ARSEF 23, and *C. militaris* Cm01 were used for genetic modifications and metabolite isolations. The WT and mutant strains were maintained on PDA (BD Difco, USA) for 2 weeks at 25°C for harvesting conidial spores. Fungi were also grown in Sabouraud dextrose broth (SDB; BD Difco) in a rotary shaker (200 rpm) for different times for metabolite isolation. The yeast strain BJ5464-NpgA was maintained on YPD medium (yeast extract at 10 g/liter, peptone at 20 g/liter, dextrose at 20 g/liter, and agar at 20 g/liter) and used for heterologous protein expression, substrate feeding, and compound identification (34). Different synthetic dropout media were used for yeast transformations.

### Fungal coculturing and HPLC analysis.

Two-week-old conidial spores of B. bassiana and *M. robertsii* were harvested from PDA plates and suspended in 0.05% Tween 20 to a concentration of 1 × 10^8^ conidia/ml. The *M. robertsii*-*B.* bassiana suspensions were mixed at 1:9, 1:1, and 9:1 volume ratios and then inoculated into SDB medium (100 ml in a 250-ml flask), each at a final concentration of 5 × 10^5^ conidia/ml, for incubation in a rotary shaker at 25°C at 200 rpm for 9 days. There were three replicates for each sample. The culture supernatants were collected by filtration and extracted with the same volume of ethyl acetate. The samples were concentrated with a rotatory concentrator (Martin Christ) under a vacuum and dissolved in 1 ml of methanol under sonication. Each sample (10 μl) was then subjected to HPLC analysis with an LC-20 AD system (Shimadzu, Japan) equipped with an SPD-20A UV-visible detector and a C_18_ reverse-phase column (particle size of 5 μm, 4.6 by 250 mm; Athena, China) (5). Samples were eluted at a flow rate of 1 ml/min with deionized water (solution A) and acetonitrile (solution B) (0 to 5 min, 15% solution B; 5 to 35 min, 15% to 100% solution B; 35 to 40 min, 100% solution B; 40 to 45 min, 100% to 15% solution B; 45 to 50 min, 15% solution B) and monitored at a wavelength of 254 nm. The column oven was set at 40°C.

### Phylogenetic analysis of the PKS-NRPS domains.

The KS and KR domains were retrieved from different fungal PKS-NRPS enzymes involved in producing 2-pyridones. The PKS-NRPS enzymes are from the fungal species B. bassiana (XP_008600657 [TenS] and GenBank accession numbers CAL69597, PQK13186, and ADN43685 [DmbS]), *B. brongniartii* (OAA40384), *C. militaris* (XP_006673463 [FarS] and GenBank accession number ATY66088), *Isaria fumosorosea* (XP_018700480 [FumoS]), and A. nidulans (Q5ATG8 [ApdA]) (21, 22, 54, 55). The sequences were aligned with the Clustal X program (version 2.0) (56). The maximum likelihood trees were generated using the JTT (Jones-Taylor-Thornton) matrix-based model and 500 bootstrap replicates with the MEGA X program (57).

### Gene expression analysis.

The harvested mycelia of *B.* bassiana, *M. robertsii*, and *M. robertsii*-*B.* bassiana at a 1:1 ratio were used for RNA extraction using the TransZol Up plus RNA kit (Transgen Biotech, China). The RNA samples were subjected to Illumina sequencing to detect differential gene expression by each fungus in coculture. For quantitative RT-PCR (qRT-PCR) verifications, cDNA samples were obtained by converting the RNA samples with the ReverTra Ace quantitative PCR (qPCR) RT master mix (Toyobo, Japan). The β-tubulin gene of B. bassiana was used as the reference (58). The expressions of the *tenS* cluster genes were individually examined by semiquantitative RT-PCR.

### Gene overexpression and deletions in different fungi.

Considering the gene cluster containing two putative transcription factor genes, *BBA_07334* and *BBA_07339* (see Table S1 in the supplemental material), overexpressions of these two genes were performed. Thus, the cDNA of each gene was amplified using the ClonExpress II one-step cloning kit (Vazyme, China) and integrated into the binary vector pDHt-Ben (conferring resistance to benomyl) by fusion PCR with different primers (Table S2). The gene was made under the control of the constitutive *gpdA* gene promoter to transform the WT strain of B. bassiana using the method of *Agrobacterium-*mediated transformation (59). The *tenR* gene was also overexpressed in *C. militaris* to obtain the Cm-OE::*tenR* transformant. The drug-resistant colonies were transferred to plates containing benomyl at a final concentration of 50 μg/ml for 2 weeks. The conidia were then used for single-spore isolation. At least five independent transformants were selected for RT-PCR verification, and the stable one with the highest expression level of the target gene was then used for further experiments.

10.1128/mbio.03279-21.8 TABLE S2 PCR primers used in this study. Download Table S2, PDF file, 0.4 MB. Copyright © 2021 Chen et al. 2021 Chen et al. https://creativecommons.org/licenses/by/4.0/ This content is distributed under the terms of the Creative Commons Attribution 4.0 International license.

To elucidate the biosynthetic pathway of 2-pyridones, we conducted individual deletions of *tenA*, *tenB*, *tenC*, and *tenS* in the OE::*tenR* mutant background. The *tenS* gene was also deleted in the WT strain of B. bassiana for different experiments. The 5′- and 3′-flanking regions of each target gene were amplified by PCR with different primer pairs (Table S2). The purified fragments were then cloned into the binary plasmid pDHt-Bar (conferring resistance to glufosinate ammonium). The obtained plasmids were then used for individual transformations of the OE::*tenR* strain. The drug-resistant (300 μg/ml of glufosinate ammonium) colonies were used for single-spore isolation and verifications.

To identify the genes involved in the methylglucosylation of tenellin analogues, we performed high-throughput RNA-seq analysis of pure *M. robertsii* and *B.* bassiana cultures and *M. robertsii*-*B.* bassiana 1:1 cocultures harvested from SDB. There were three biological repeats for each sample. The mycelia were harvested for RNA extraction, and 1 μg RNA from each sample was used for the generation of the library using the Illumina TruSeq kit. The libraries were sequenced using the Illumina HiSeq platform, and the clean reads were used for gene mapping and expression analysis by calculating the index of the fragments per kilobase of exon per million reads mapped. Relative to the *B.* bassiana pure cultures, the upregulated glycosyltransferase (GT) and methyltransferase (MT) genes were either individually or jointly deleted in the OE::*tenR* strain. The homologous *GT/MT* genes were also deleted in the WT strain of *M. robertsii* for substrate feeding assays. To further determine the functions of *BbGT1* and *BbMT1*, cDNAs of these two genes were cloned by fusion PCR into the yeast plasmids pXK30F (*Leu*^−^) and pXW06F (*Trp*^−^), respectively. The obtained vectors were either individually or jointly transformed into the yeast strain BJ5464 (34). Likewise, the Metarhizium *MrGT1* and *MrMT1* genes were also cloned into this yeast strain.

### Metabolite isolation and purifications.

For metabolite purifications, a total of 5 liters of the *B.* bassiana-*M. robertsii* (1:1) coculture was obtained after incubation in SDB (300 ml per 1-liter flask) at 25°C at 200 rpm for 2 weeks. Culture filtrates were extracted with ethyl acetate. The pooled samples were concentrated by rotary evaporation and redissolved in methanol. The aliquots (150 μl) were repeatedly loaded into the LC-20 AD HPLC system equipped with a semipreparative C_18_ reverse-phase column (particle size of 5 μm, 10 by 250 mm; Athena, China). Eluates were maintained at a flow rate of 3 ml/min with deionized water (solution A) and acetonitrile (solution B) (0 to 5 min, 15% solution B; 5 to 25 min, 15% to 100% solution B; 25 to 27 min, 100% solution B; 27 to 29 min, 100% to 15% solution B; 29 to 30 min, 15% solution B). Ten fractions were obtained after separation by preparative HPLC. Compounds 1 to 7 (all as faint yellow powders) were then purified by analytical HPLC as indicated above. The OE::*tenR* Δ*tenA* mutant was also used for fermentation at up to 5 liters, and compounds 8 to 13 (all as faint yellow powders) were purified using similar protocols. The OE::*tenR* Δ*tenB* mutant was fermented for purification and structure identification of compound 2. During the trial HPLC analysis, a strong peak was produced by the *C. militaris* transformant Cm-OE::*tenR*. The mutant was thus fermented to a large volume (5 liters) for compound purification according to the same methods. During the yeast feeding assay with compound 3, a novel peak (termed compound 1′) was found. For elucidation of its structure, yeast::*BbGT1/MT1* cells were fermented in the Synthetic Drop-out (SD)−Leu/−Trp medium for 16 h to reach an optical density at 600 nm (OD_600_) value of 0.6, and compound 3 was then added to a final concentration of 10 μg/ml for additional incubation for 48 h at 30°C at 220 rpm. The supernatant was collected by centrifugation and then used for metabolite extraction with ethyl acetate. Compound 1′ was then purified according to the same steps.

### Compound structure identification.

The purified compounds were individually subjected to spectrum analysis for structure identification. The high-resolution electrospray ionization mass spectrometry (HRESIMS) spectrum of the compound was recorded with an Agilent QTOF 6545 instrument operated in a positive-ion mode at capillary and cone voltages of 3.6 kV and 40 to 150 V, respectively. The collision energy was optimized from 15 to 50 V. For accurate measurement of metabolite mass, the instrument was calibrated each time using a standard calibration mix (Agilent) in the range of *m/z* 150 to 1,900. The 1D and 2D NMR (nuclear magnetic resonance) spectrum data for each compound (including ^1^H, ^13^C, heteronuclear single quantum coherence [HSQC], correlation spectroscopy [COSY], nuclear Overhauser effect spectroscopy distortionless enhancement by polarization transfer [NOESY DEPT], and/or heteronuclear multiple-bond correlation [HMBC]) were recorded at 25°C using a Bruker Avance III-500 spectrometer equipped with a 5-mm Pabbo BB-^1^H/D probe (60). The chemical shift values (δ) are given in parts per million, and the coupling constants (*J* values) are shown in hertz. Chemical shifts were referenced to the residual solvent peaks of CD_3_OD-*d*_4_. All spectra were processed with the MestRe Nova program (version 9.0.1; Metrelab Research, Santiago de Compostela, Spain).

### Substrate feeding and cross-conversion assays.

The WT and Δ*MrGT1* strains of *M. robertsii* were grown in SDB for 3 days at 25°C at 200 rpm, and compound 3 or 4 was then added to a final concentration of 10 μg/ml for an additional 2 days. The culture filtrates were analyzed by HPLC to examine the possibility of cross-modification of the compounds by *M. robertsii*. In addition, transgenic yeast cells with *BbGT1/MT1*, *MrGT1/MT1*, or individual GT or MT genes were fed with compound 2 or 3 or farinosone B for possible conversion assays. The corresponding yeast cells were first gown in alternative SD media to reach an OD_600_ value of 0.6 prior to the addition of the compound to a final concentration of 10 μg/ml for 2 days. The WT and Δ*MrGT1* strains of *M. robertsii* were also cocultured with either the WT or OE::*tenR* Δ*BbGT1/MT1* strain of B. bassiana in SDB for 9 days to profile metabolite production by HPLC.

### Fungal competition and insect bioassays.

The spores of the WT and different mutant strains (including Δ*tenS*, OE::*tenR*, and OE::*tenR* Δ*BbGT1/MT1*) of B. bassiana and the WT strain of *M. robertsii* were inoculated in SDB at a final concentration 5 × 10^5^ conidia/ml (100 ml per 250-ml flask) for 4 days. The culture samples (5 ml each) of B. bassiana were then transferred into a sterile flask (50 ml). The *M. robertsii* culture (5 ml each) was transferred to sterile and one-end-sealed regenerated cellulose dialysis tubing (1- to 50-kDa-molecular-weight cutoff [MWCO]; Fisher Scientific, USA). The sample tubes were sealed and put into the flasks containing B. bassiana cultures for coculturing without mycelial contacts. After incubation at 25°C at 200 rpm for a week, the tubes containing Metarhizium cultures were carefully taken out. The mycelia of B. bassiana strains were collected and washed three times with sterile water. The WT and mutants of B. bassiana were also grown in SDB without *M. robertsii* for 11 days for biomass comparisons. The samples were then dried at 65°C overnight and weighed. There were three replicates for each treatment. The mycelial samples (20 to 30 mg each) of B. bassiana were then treated with nitric acid (1 ml; 98%) for 24 h. The samples were diluted with deionized water (12.5 ml each) prior to iron quantification analysis using an inductively coupled plasma mass spectrometry (ICP-MS) system (Nexion 300D; PerkinElmer [PE]) (61). There were three replicates for each sample, and the experiments were repeated twice. One-way analysis of variance (ANOVA) was conducted to compare the mycelial biomass differences between samples.

Insect bioassays were performed using the WT, Δ*tenS*, and OE::*tenR* strains of B. bassiana against last-instar larvae of the wax moth (Galleria mellonella) and the W1118 line of female adults of the fruit fly (Drosophila melanogaster) (2 days posteclosion) (62). The conidial spores of the different strains were harvested from the 2-week-old PDA plates and suspended in 0.05% Tween 20. Spore concentrations were adjusted to 3 × 10^7^ conidia/ml for immersion assays against wax moth larvae and 2 × 10^7^ conidia/ml for immersion assays against fruit flies. There were three replicates (15 insects per replicate) for each strain, and the experiments were repeated twice. Treatments with 0.05% Tween 20 were used as the mock control. The estimations of the median lethal time (LT_50_) and differences in insect survival were conducted by Kaplan-Meier analysis (5).

### Iron chelation and resistance assays.

To examine the iron chelation ability of compound 3 (15-HT) and compound 4 (1-*O*-methyl-hydryoxtenellin), we mixed the compounds individually with FeCl_3_ at a molar ratio of 3:1 for 30 min. The samples were then subjected to liquid chromatography-mass spectrometry (LC-MS) analysis using a Q Exactive mass spectrometer (Thermo Fisher, USA). For iron resistance and depletion assays, PDA plates (9 cm in diameter) were amended with FeSO_4_ (at final concentrations of 5 and 10 mM), FeCl_3_ (2 and 4 mM), and the iron chelator 1,10-phenanthroline (50 and 100 μM) (Sigma-Aldrich). Spores of the WT and mutants (Δ*tenS*, OE::*tenR*, and OE::*tenR* Δ*BbGT1/MT1*) of B. bassiana were harvested from the 2-week-old PDA plates and adjusted in 0.05% Tween 20 to final concentrations of 1 × 10^7^ conidia/ml. Spore suspensions (2 μl each) of the WT and mutants were then inoculated as a pair on each PDA plate. After incubation for 2 weeks, the diameter of the culture colonies was measured. Spore germination assays were also conducted in SDB and SDB with the addition of FeSO_4_ (3 mM) or phenanthroline (20 μM) in combination with 15-HT (20 μM). To test the effect of 2-pyridone production on fungal competition, both WT and Δ*tenS* spores were mixed at a 1:1 ratio with conidia of *M. robertsii* with and without the addition of the purified 15-HT at final concentrations of 10 μM and 20 μM in SDB. Spore germinations were determined and compared after culturing at 25°C at 200 rpm for 12 h. There were three replicates for each treatment. The growth and germination differences between strains were compared using either one-way ANOVA or two-tailed Student’s *t* test.

### Data availability.

The RNA-seq data from fungal cocultures have been deposited in the NCBI database with BioProject accession number PRJNA716748.
