# Supplementary material for: Inductive Production of the Iron-Chelating 2-Pyridones Benefits the Producing Fungus To Compete for Diverse Niches
Source: mBio. 2021 Dec 14;12(6):e03279-21. doi: 10.1128/mbio.03279-21 (PMC8669486; doi:10.1128/mbio.03279-21)
Supplement: TABLE S1 [file mbio.03279-21-st001.pdf]

TABLE S1 Conserved gene clusters involved in biosynthesis of 2-pyridones in different fungi.

B. bassiana	***B. brongniartii***	***C. militaris***	***I. fumosorosea***	***A. nidulans***	**Annotation**
BBA_07334, *tenR*	BBO_05972	CCM_08264, *farR*	ISF_08694, *fumoR*	AN8414, *apdR*	Fungal zinc cluster transcription factor
BBA_07335, *tenA*	BBO_05971	CCM_08263, *farA*	ISF_08693, *fumoA*	AN8411, *apdE*	Cytochrome P450 CYP655C1
BBA_07336, *tenB*	BBO_05970	CCM_08262, *farB*	ISF_08692, *fumoB*	AN8408, *apdB*	Cytochrome P450 CYP623C1
BBA_07337, *tenC*	BBO_05969	CCM_08262, *farC*	ISF_08691, *fumoC*	AN8409, *apdC*	Enoyl reductase
BBA_07338, *tenS*	BBO_05968	CCM_08261, *farS*	ISF_08690, *fumoS*	AN8412, *apdA*	PKS-NRPS synthase
BBA_07339	BBO_05967	CCM_08260	ISF_08689	–	Putative Zn(II)2Cys6 transcription factor
–	–	–	–	AN8413, *adpF*	Aspyridones efflux protein
–	–	–	–	AN8415, *apdG*	Acyl-CoA dehydrogenase
