# Supplementary material for: Inductive Production of the Iron-Chelating 2-Pyridones Benefits the Producing Fungus To Compete for Diverse Niches
Source: mBio. 2021 Dec 14;12(6):e03279-21. doi: 10.1128/mbio.03279-21 (PMC8669486; doi:10.1128/mbio.03279-21)
Supplement: DATA SET S1 [file mbio.03279-21-sd001.pdf]

^{1}H (500 MHz) and ^{13}C (100 MHz) NMR Data for compounds **1** and **1'**. Measured in CD_3OD.

**Position**	**1 (500 Hz)**		**1' (500 Hz)**	
	δH, mult (*J* in Hz)	δC	δH, mult (*J* in Hz)	δC
2	-	160.1	-	160.2
3	-	108.3	-	108.3
4	-	176.6	-	176.6
5	-	114.1	-	115.5
6	8.10, s	143.0	8.08, s	143.0
7	-	196.0	-	195.7
8	7.97, d (15.5)	124.3	7.94, d (15.5)	124.3
9	7.64, d (15.5)	151.6	7.61, d (15.5)	151.5
10	-	137.2	-	137.1
11	5.91, d (11.5)	147.9	5.87, d (10.0)	147.9
12	2.65, m	45.1	2.64, m	45.1
13	1.66, m; 1.31,m	25.4	1.66, m; 1.30, m	25.5
14	0.91, t (7.5)	12.2	0.90, t (7.5)	12.1
15	3.52, m	66.0	3.52, m	66.1
16	1.96, s	13.1	1.94, s	13.3
17	-	124.8	-	124.6
18/22	7.33, d (8.5)	131.9	7.32, d (8.5)	131.7
19/21	6.83, d (8.5)	116.3	6.82, d (8.5)	116.2
20	-	158.6	-	158.5
1'	5.03, d (8.0)	108.4	5.04, d (8.5)	108.6
2'	3.43, m	73.3	3.44, m	73.6
3'	3.59, m	77.3	3.49, m	77.7
4'	3.20, t (9.0)	79.9	3.40, m	70.6
5'	3.49, m	77.6	3.38, m	78.6
6'	3.81, dd (12.0, 1.5);<br>3.71, dd (12.0, 4.5)	61.8	3.85, m;<br>3.73, m	62.2
4'- OCH_3	3.57, s	60.8	-	-
N- OCH_3	-	-	-	-

^{1}H (400/500 MHz) and ^{13}C (100 MHz) NMR Data for compounds **2** and **3**. Measured in CD_3OD.

**Position**	**2**		**3**	
	δH, mult (*J* in Hz)	δC	δH, mult (*J* in Hz)	δC
2	-	164.5	-	160.2
3	-	107.7	-	107.6
4	-	178.1	-	174.3
5	-	116.4	-	114.1
6	7.47, s	140.9	7.98, s	140.5
7	-	196.1	-	196.0
8	7.98, d (15.5)	125.3	8.04, d (15.5)	125.2
9	7.59, d (15.5)	150.9	7.62, d (15.5)	151.1
10	-	137.2	-	137.2
11	5.87, d (10.0)	147.3	5.88, d (11.0)	147.5
12	2.65, m	45.1	2.65, m	45.1
13	1.66, m; 1.30, m	25.5	1.67, m; 1.31,m	25.5
14	0.90, t (10.0)	12.1	0.91, t (7.5)	12.1
15	3.52, m	66.1	3.52, m	66.1
16	1.95, s	13.3	1.96, s	13.3
17	-	124.7	-	124.7
18/22	7.29, d (8.5)	131.4	7.32, d (8.5)	131.5
19/21	6.81, d (8.5)	116.1	6.82, d (8.5)	116.2
20	-	158.3	-	158.4

^1H (400/500 MHz) and ^{13}C (100 MHz) NMR Data for compounds **4**, **5** and **6**. Measured in CD_3OD.

Position	**4**		**5**		**6**	
	δH, mult (*J* in Hz)	δC	δH, mult (*J* in Hz)	δC	δH, mult (*J* in Hz)	δC,
2	-	159.6	-	159.7	-	160.4
3	-	108.2	-	108.4	-	107.6
4	-	176.1	-	176.2	-	174.0
5	-	115.6	-	115.7	-	114.3
6	8.08, s	140.5	7.97, s	139.6	7.97, s	140.7
7	-	195.7	-	196.1	-	196.1
8	7.99, d (13.2)	124.3	8.02, d (15.5)	127.1	8.02, d (15.5)	125.1
9	7.64, d (13.2)	151.3	7.58, d (15.5)	143.0	7.58, d (15.5)	151.7
10	-	137.2	-	135.2	-	134.6
11	5.90, d (9.2)	147.8	5.86, d (9.0)	145.0	5.86, d (9.0)	151.3
12	2.67, m	45.0	2.56, m	44.1	2.56, m	36.4
13	1.66, m; 1.33, m	25.5	1.47, m; 1.36, m	25.6	1.47, m; 1.36, m	31.0
14	0.91, t (7.6)	12.1	0.90, t (7.5)	12.1	0.90, t (7.5)	12.5
15	3.52, m	66.2	1.03, d (7.0)	66.5	1.03, d (7.0)	20.5
16	1.97, s	13.3	1.92, s	20.7	1.92, s	13.0
17	-	124.7	-	124.4	-	124.9
18/22	7.33, d (8.4)	131.5	7.32, d (8.5)	131.6	7.32, d (8.5)	131.5
19/21	6.83, d (8.4)	116.3	6.82, d (8.5)	116.2	6.82, d (8.5)	116.2
20	-	158.5	-	158.6	-	158.5
N- OCH_3	4.06, s	65.3	4.05, s	65.8	-	-

^{1}H (400/500 MHz) and ^{13}C (100 MHz) NMR Data for compounds **7**, **8** and **9**. Measured in CD3OD.

**Pos.**	**7**		**8**		**9**	
	δH, mult (*J* in Hz)	δC	δH, mult (*J* in Hz)	δC	δH, mult (*J* in Hz)	δC
2	-	176.5	-	176.7	-	176.6
3	-	101.5	-	101.2	-	101.3
4	-	197.6	-	197.4	-	197.2
5	4.05, s	64.3	4.07, s	64.3	4.04, s	64.4
6	-	176.0		176.0	-	175.8
7	7.05, d (15.6)	116.9	7.08, d (15.5)	117.0	7.08, d (15.6)	116.9
8	7.46, d (15.6)	151.0	7.49, d (15.5)	151.0	7.49, d (15.6)	150.9
9	-	135.0	-	134.5	-	136.7
10	5.93, d (10.0)	149.4	5.88, d (9.5)	152.5	5.88, d (10.0)	148.7
11	2.61, m	42.4	2.79, m	31.3	2.63, m	45.1
12	3.60, m	72.1	1.55, m; 1.64, m	40.0	1.55, m; 1.62, m	25.4
13	1.12, d (6.4)	21.3	3.48, m; 3.53, m	60.7	0.87, t (10.0)	12.0
14	1.05, d (6.4)	16.5	1.04, d (11.0)	20.3	3.47, m; 3.54, m	65.9
15	1.87, s	12.7	1.89, s	12.4	1.91, s	12.9
16	2.82, dd(14.0, 6.4)<br>2.99, dd(14.0, 2.0)	37.6	2.80, dd14.0, 6.0)<br>2.86, dd(14.0, 4.0)	37.3	2.84, m<br>3.01, dd (11.2, 3.2)	37.5
17	-	127.0	-	127.8	-	127.9
18/22	6.98, d (8.4)	131.7	6.99, d (8.0)	131.7	6.99, d (8.0)	131.7
19/21	6.68, d (8.4)	116.1	6.67, d (8.0)	116.1	6.67, d (8.0)	116.1
20	-	157.0	-	157.1	-	157.4

^{1}H (500 MHz) and ^{13}C (100 MHz) NMR Data for **farinosone B.** Measured in CD_3OD.

Position	Farinosone B	
	δH, mult (*J* in Hz)	δC
2	-	159.9
3	-	107.6
4	-	174.5
5	-	114.2
6	7.94, s	140.1
7	-	195.0
8	7.98, d (15.0)	124.7
9	7.63, dd (12.0, 14.5)	145.6
10	6.54, m	131.4
11	6.82, m	144.8
12	6.37, dd (11.0, 15.0)	128.6
13	6.51, m	147.0
14	-	134.5
15	5.46, d (10.0)	144.7
16	2.47, m	35.7
17	1.43, m; 1.30, m	12.4
18	0.87, t (7.5)	20.8
19	0.99, d (6.5)	12.8
20	1.82, s	158.5
21	-	127.3
22/26	7.31, d (9.0)	131.5
23/25	6.82, d (9.0)	116.2
24	-	158.4

^{1}H (400/500 MHz) and ^{13}C (100 MHz) NMR Data for compounds **10**, **11** and **12**. Measured in CD_3OD.

Pos.	**10**		**11**		**12**	
	δH, mult (*J* in Hz)	δC	δH, mult (*J* in Hz)	δC	δH, mult (*J* in Hz)	δC
2	-	176.5	-	176.5	-	176.1
3	-	102.1	-	104.7	-	104.5
4	-	197.6	-	197.8	-	198.0
5	4.09, s	64.0	4.10, s	64.2	4.25, s	68.6
6	-	176.0	-	175.7	-	176.0
7	7.16, d (15.5)	117.3	7.17, d (15.5)	118.4	6.99, d (15.5)	116.7
8	7.49, d (15.5)	151.9	7.49, d (15.5)	149.4	7.45, d (15.5)	151.1
9	-	136.0	-	137.0	-	134.3
10	6.06, s	151.6	5.96, d (9.5)	143.3	5.85, d (9.5)	153.2
11	-	74.3	3.73, m	47.6	2.53, m	36.5
12	1.68, m	36.9	-	210.5	1.33, m; 1.45, m	31.0
13	0.94, t (7.5)	8.7	2.15, s	28.5	0.87, t (7.5)	12.3
14	1.36, s	28.3	1.20, d (7.0)	16.4	1.00, d (7.0)	20.2
15	2.11, s	13.0	1.97, s	12.6	1.86, s	12.5
16	2.88, dd (14.5, 5.0)<br>3.01, dd (14.5, 3.5)	37.6	2.87, dd (14.0, 6.0)<br>3.00, dd (14.0, 4.5)	37.5	5.00, d (3.5)	74.8
17	-	127.8	-	127.7	-	130.6
18/22	6.99, d (8.0)	131.7	6.99, d (8.0)	131.7	7.13, d (8.5)	129.6
19/21	6.67, d (8.0)	116.1	6.66, d (8.0)	116.1	6.68, d (8.5)	115.6
20	-	157.3	-	157.3	-	158.2

^1H (500 MHz) and ^{13}C (100 MHz) NMR Data for compound **13**. Measured in CD3OD.

**Position**	**13**	
	δH, mult (*J* in Hz)	δC
2	-	159.5
3	-	108.6
4	-	175.2
5	-	115.4
6	8.13, s	141.3
7	-	206.3
8	3.66, m; 3.55, m	66.2
9	4.66, t (11.0)	74.4
10	-	135.9
11	5.60, d (10.0)	126.7
12	2.05, m	37.7
13	1.32, m	26.6
14	0.96, t (7.0)	11.6
15	1.76, s	19.6
16	-	124.2
17/21	7.34, d (7.5)	131.6
18/20	6.84, d (7.5)	116.2
19		158.6
N- OCH_3	4.07, s	65.8
