# Supplementary material for: Inductive Production of the Iron-Chelating 2-Pyridones Benefits the Producing Fungus To Compete for Diverse Niches
Source: mBio. 2021 Dec 14;12(6):e03279-21. doi: 10.1128/mbio.03279-21 (PMC8669486; doi:10.1128/mbio.03279-21)
Supplement: DATA SET S2 [file mbio.03279-21-sd002.pdf]

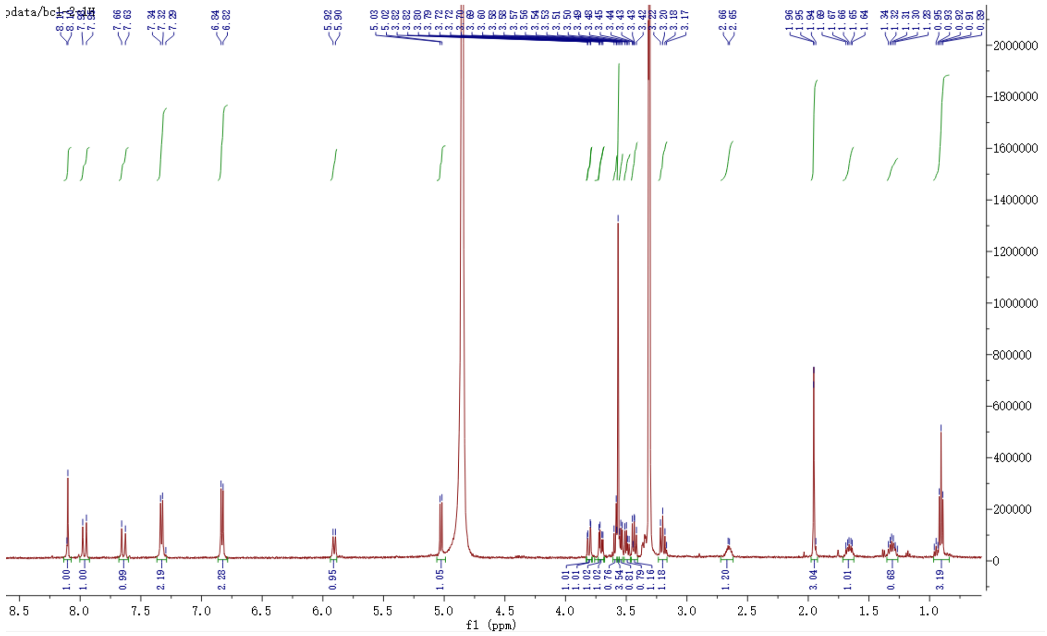

^{1}H NMR spectrum of Pyridovericin-*N*-*O*-(4-O-methyl-β-D-glucopyranoside) (**1**) (500 Hz, CD3OD-d_4).

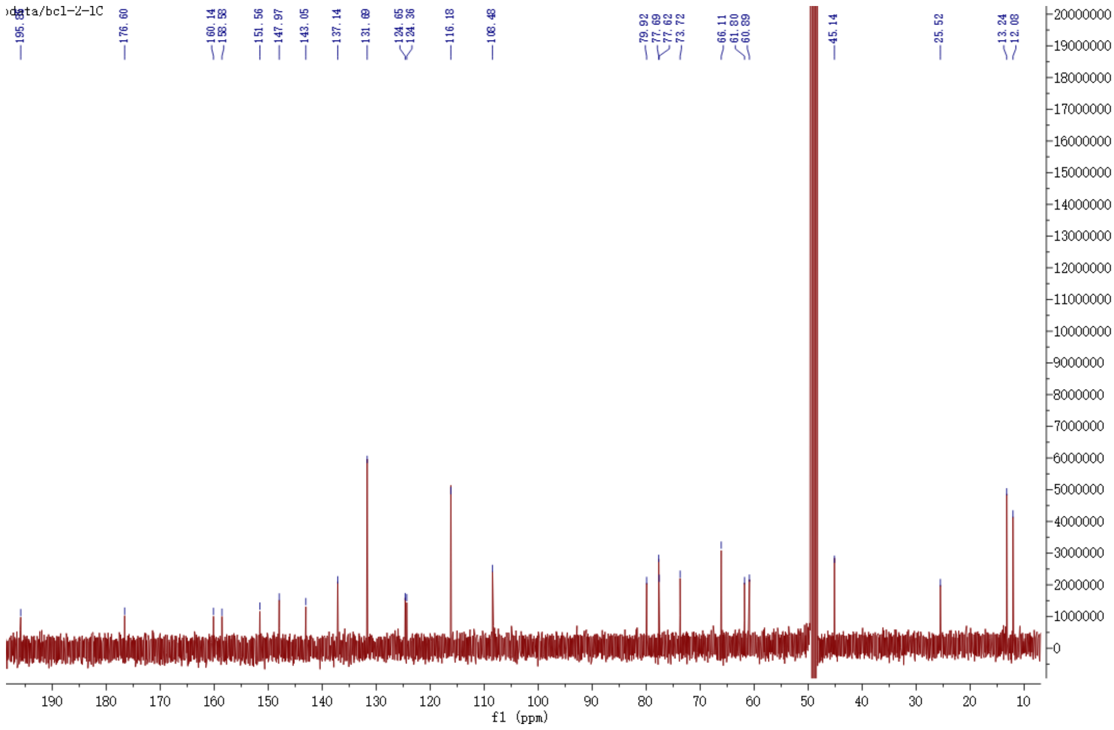

^{13}C NMR spectrum of Pyridovericin-*N*-*O*-(4-O-methyl-β-D-glucopyranoside) (**1**) (100 Hz, CD3OD-d_4).

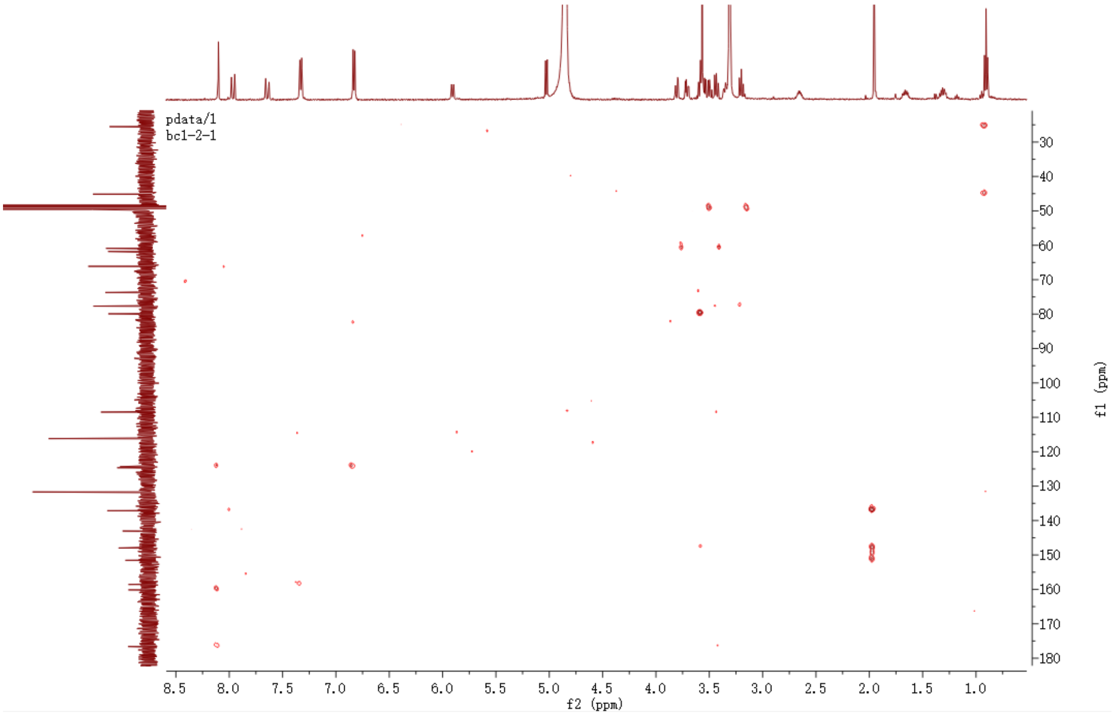

HMBC spectrum of Pyridovericin-*N*-*O*-(4-O-methyl-β-D-glucopyranoside) (**1**).

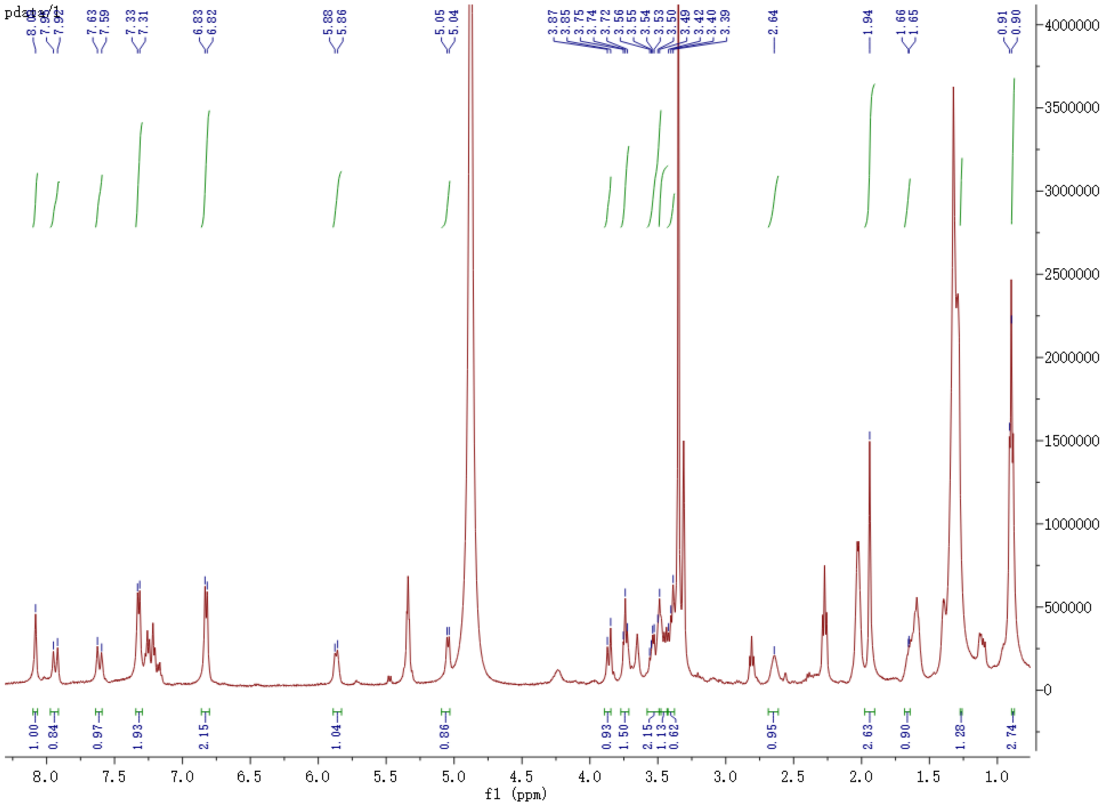

^{1}H NMR spectrum of Pyridovericin-*N*-*O*-(β-D-glucopyranoside) (**1'**) (500 Hz, CD_3OD-*d*$_4$).

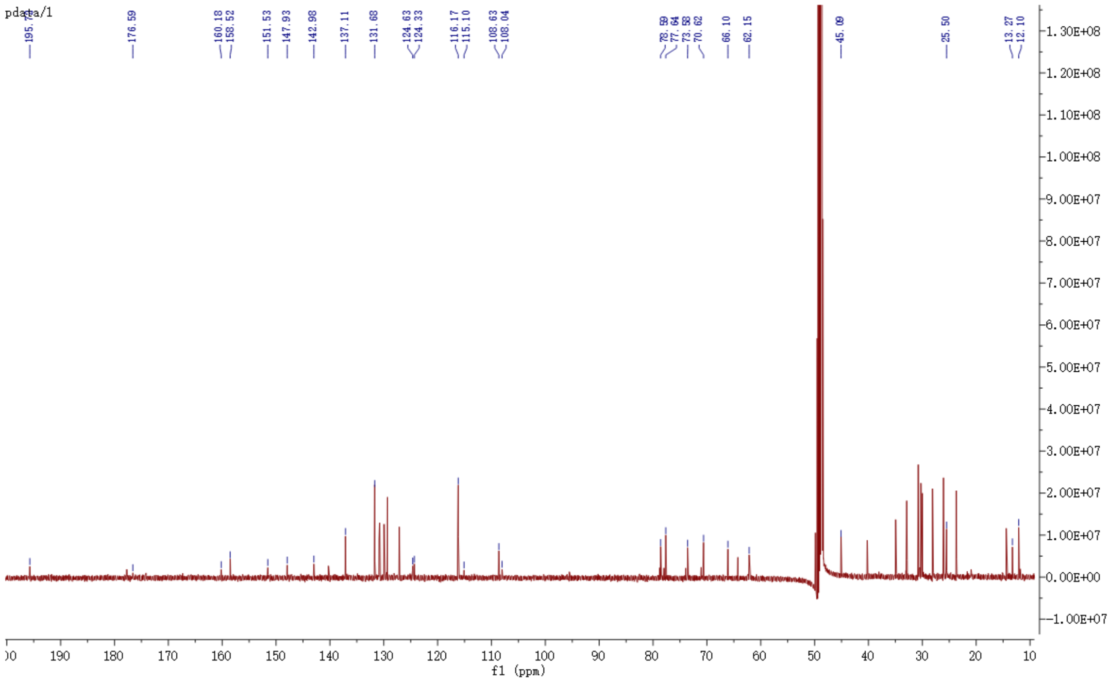

^{13}C NMR spectrum of Pyridovericin-*N*-*O*-(β-D-glucopyranoside) **(1')** (100 Hz, CD_3OD-*d_4*).

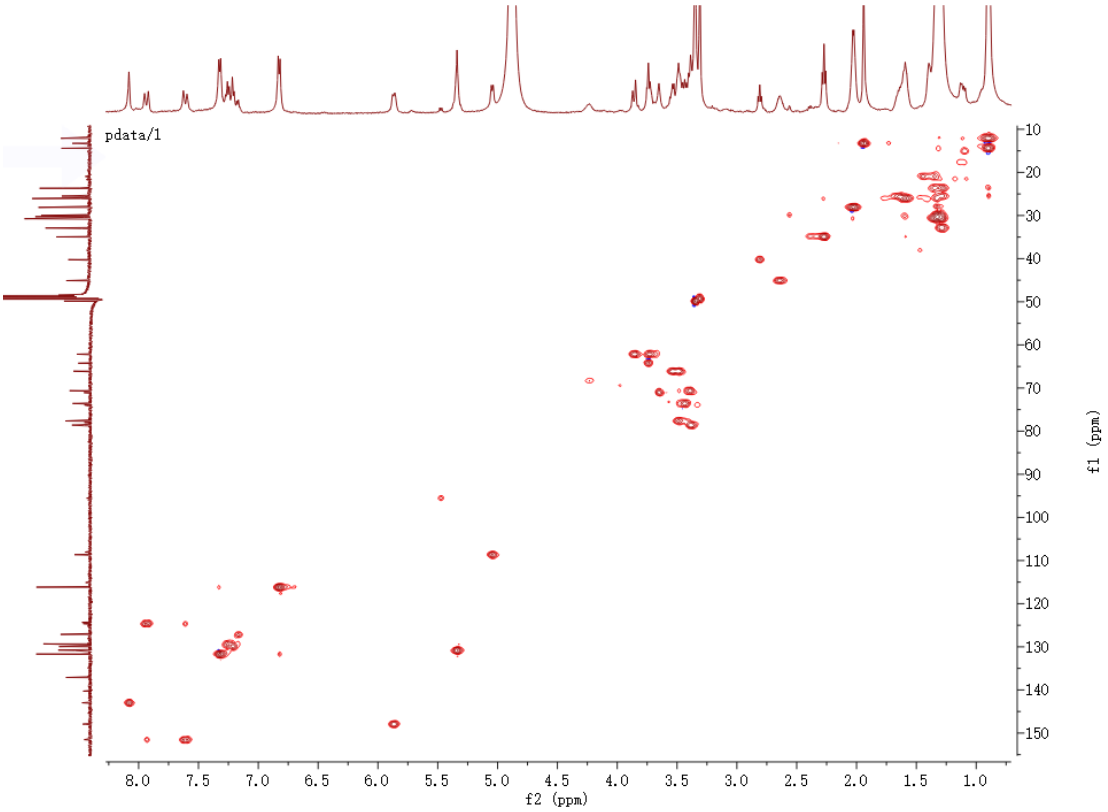

HSQC spectrum of Pyridovericin-*N*-*O*-(β-D-glucopyranoside) (**1'**).

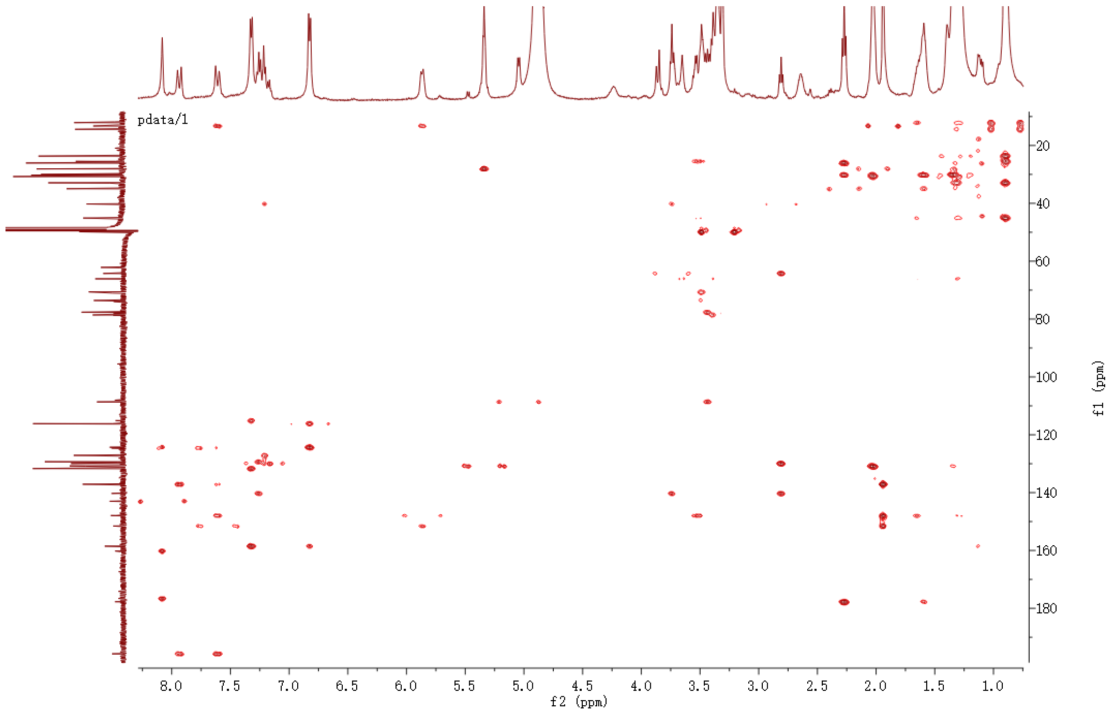

HMBC spectrum of Pyridovericin-*N*-*O*-(β-D-glucopyranoside) (**1'**).

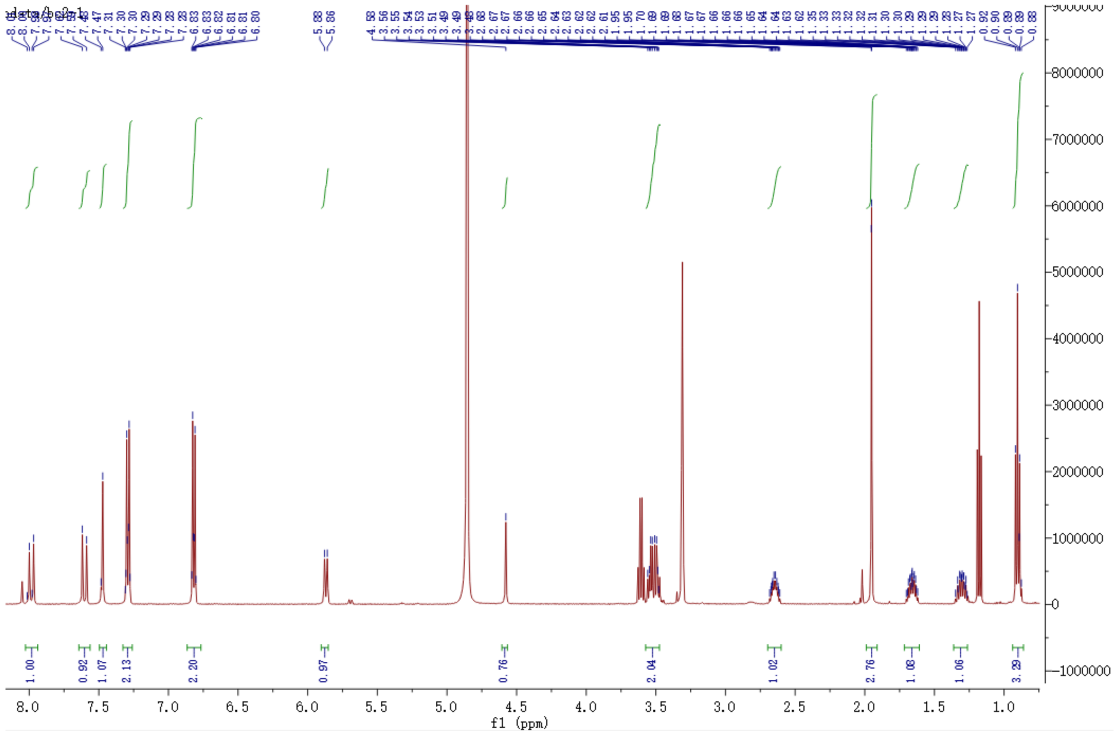

^{1}H NMR spectrum of pyridovericin **(2)** (500 Hz, CD3OD-d_4).

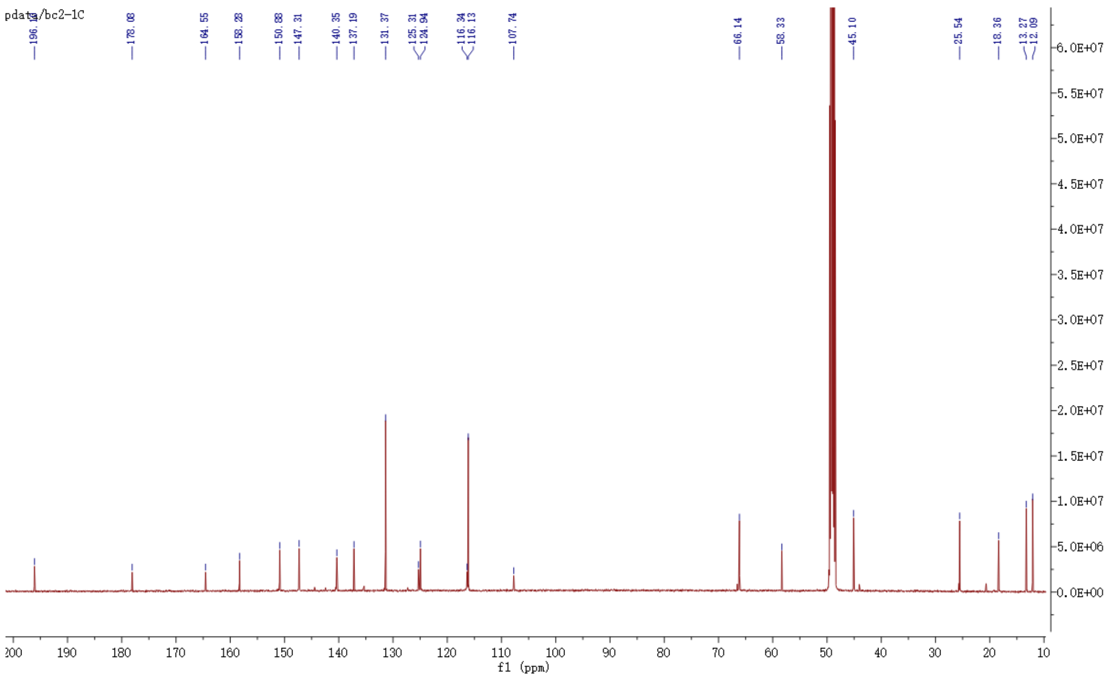

^{13}C NMR spectrum of pyridovericin **(2)** (100 Hz, CD3OD-d_4).

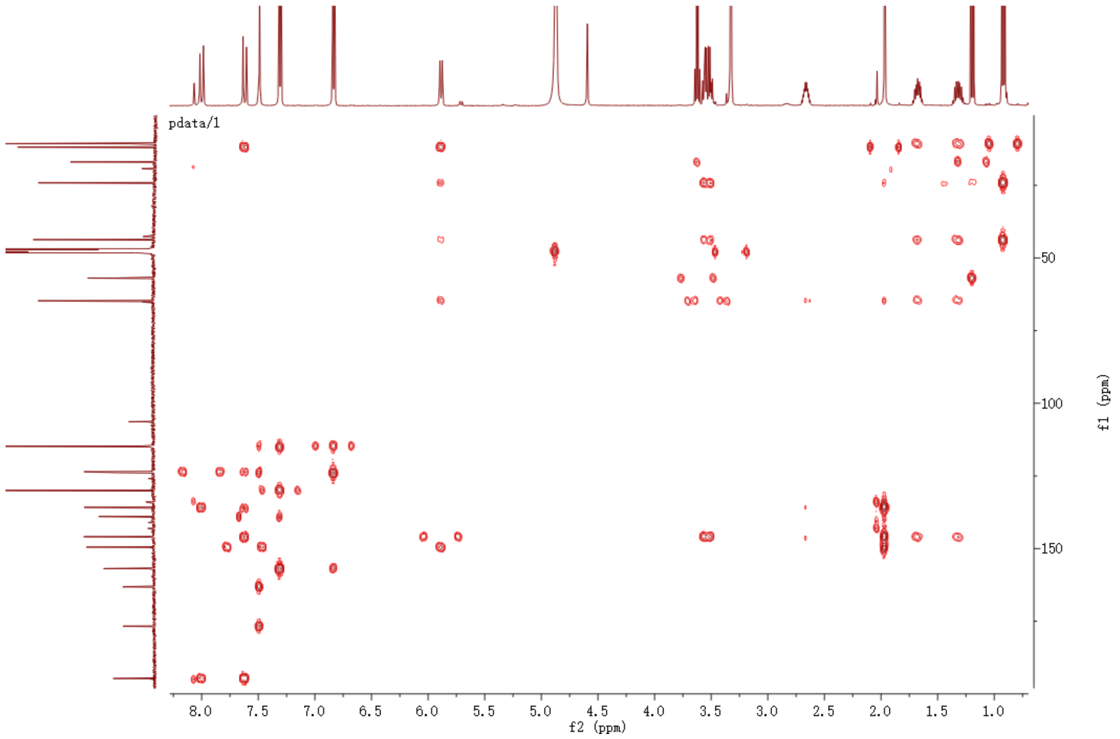

HMBC spectrum of pyridovericin (**2**).

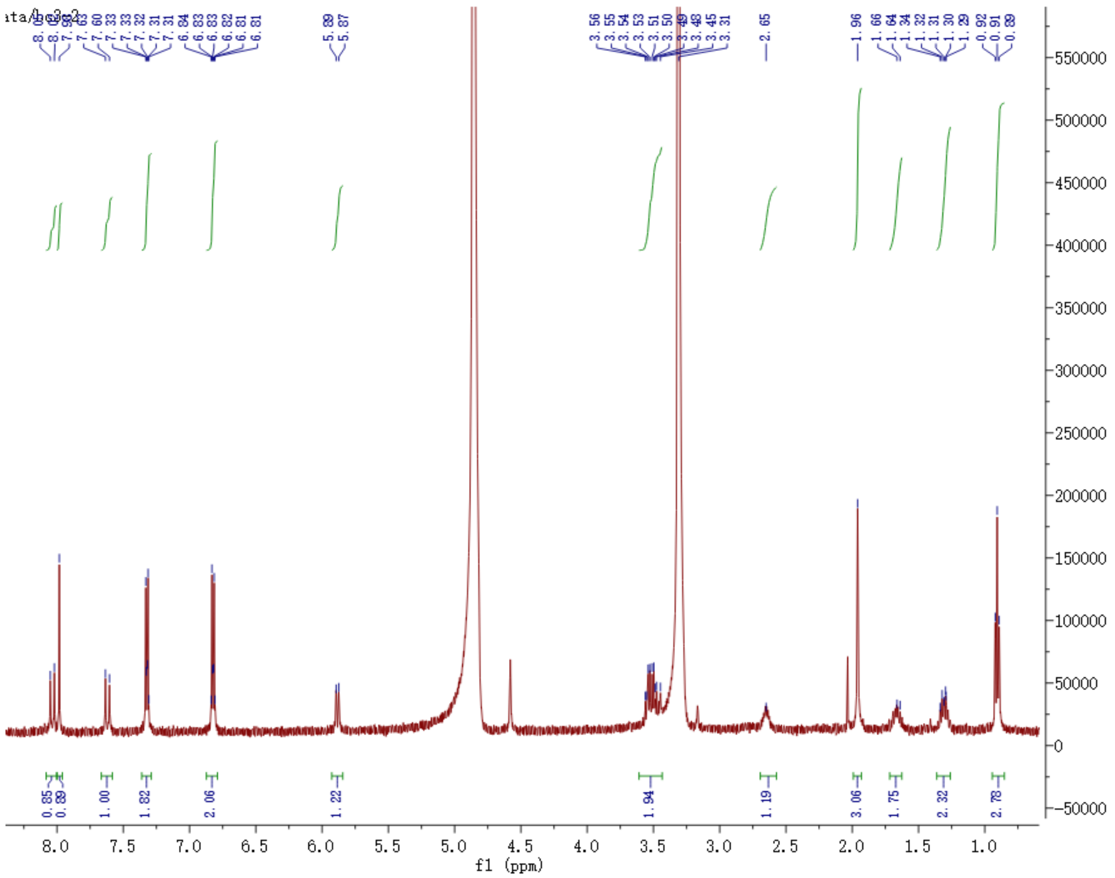

^{1}H NMR spectrum of 15-hydroxytenellin **(3)** (500 Hz, CD3OD-*d₄*).

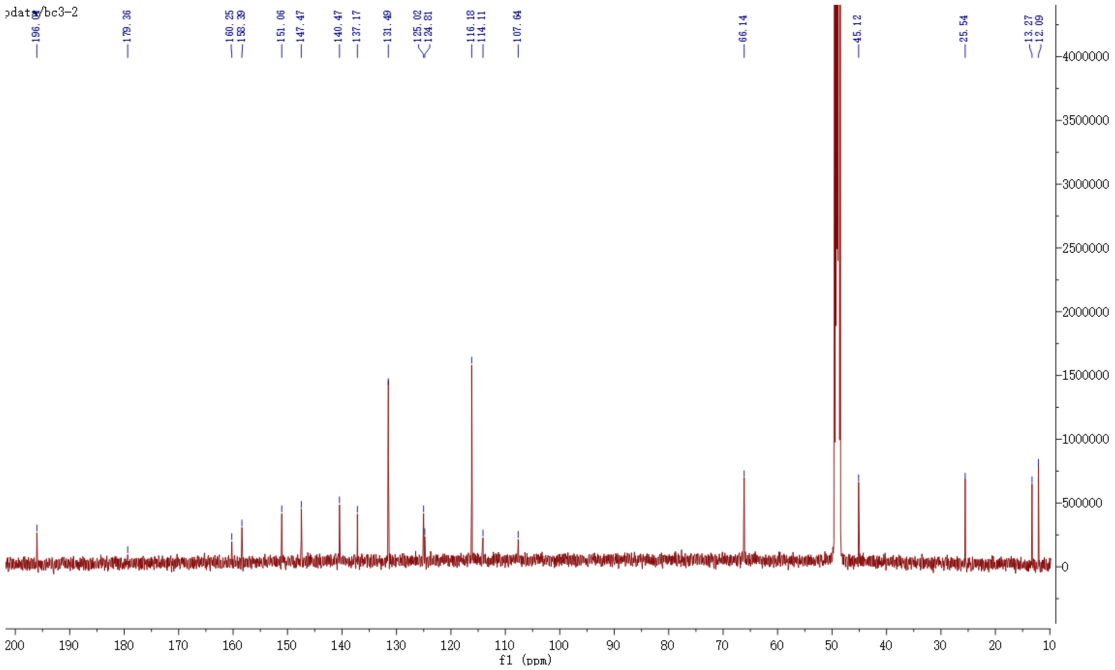

^{13}C NMR spectrum of 15-hydroxytenellin **(3)** (100 Hz, CD3OD-*d₄*).

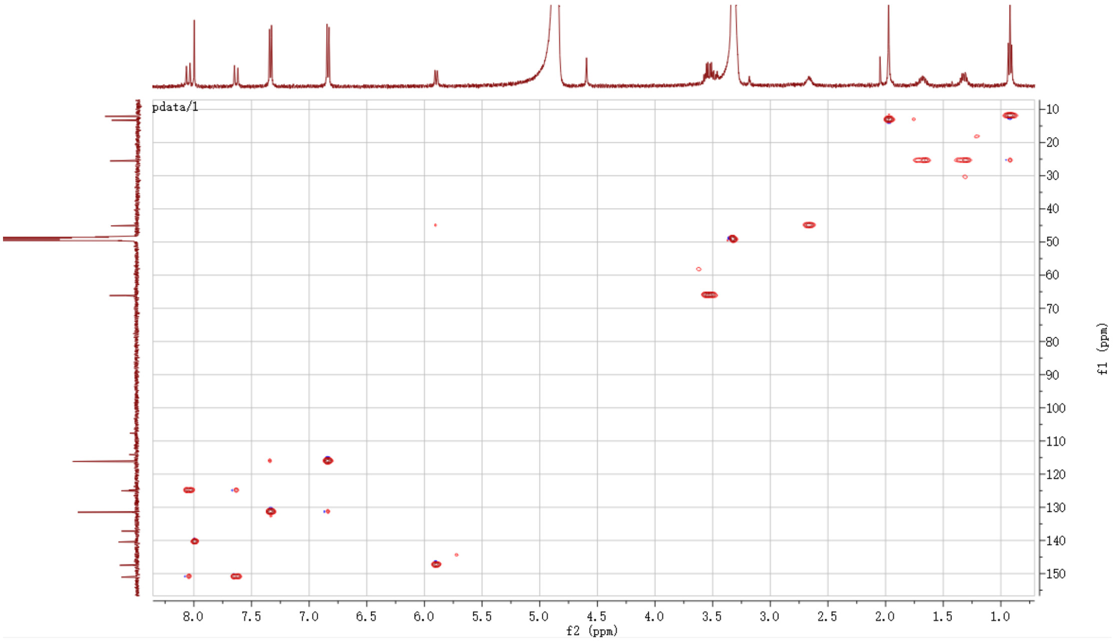

HSQC spectrum of 15-hydroxytenellin (**3**).

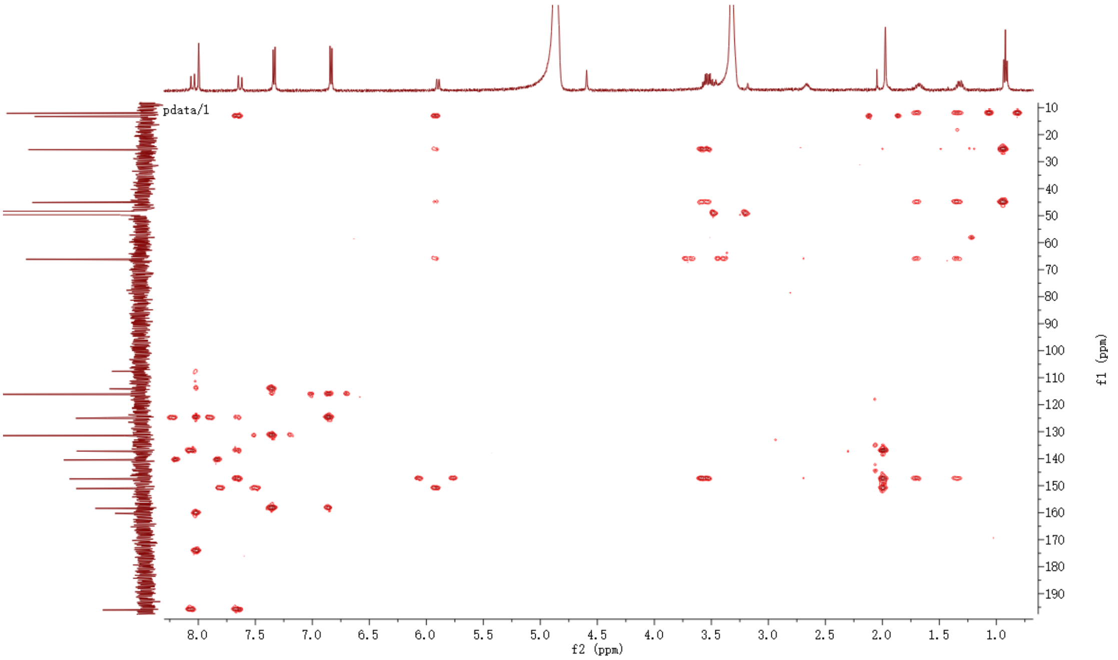

HMBC spectrum of 15-hydroxytenellin (**3**).

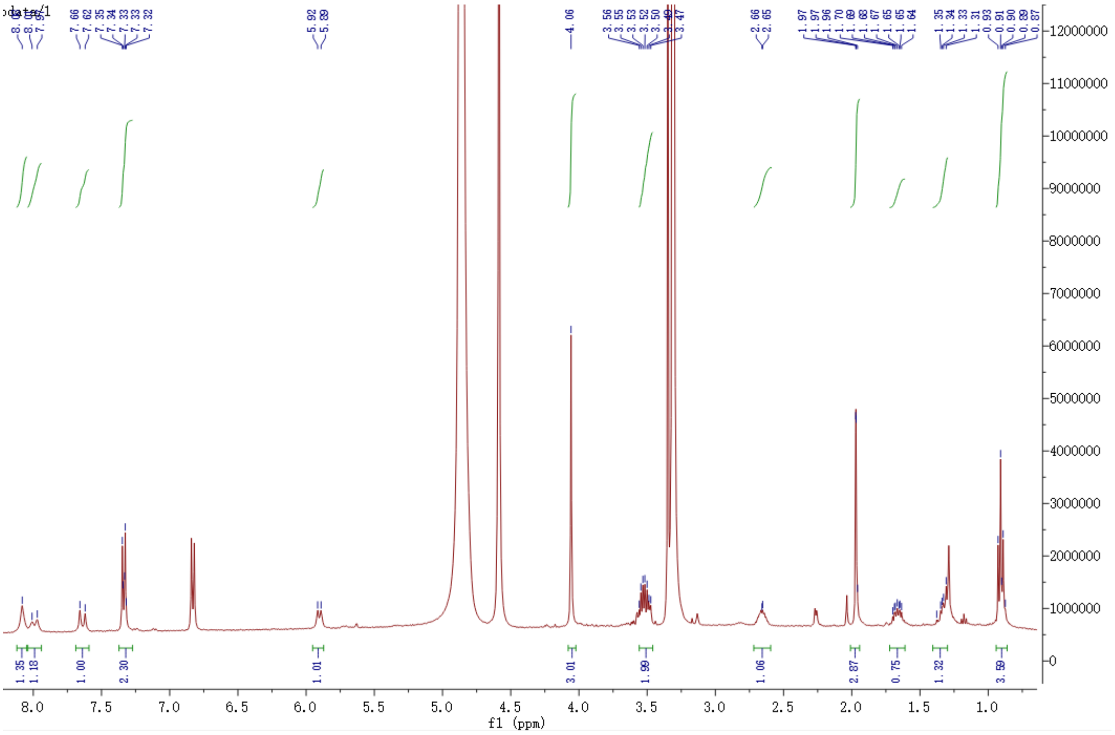

^{1}H NMR spectrum of 1-O-methyl-15-hydroxytenellin **(4)** (400 Hz, CD3OD-d_4).

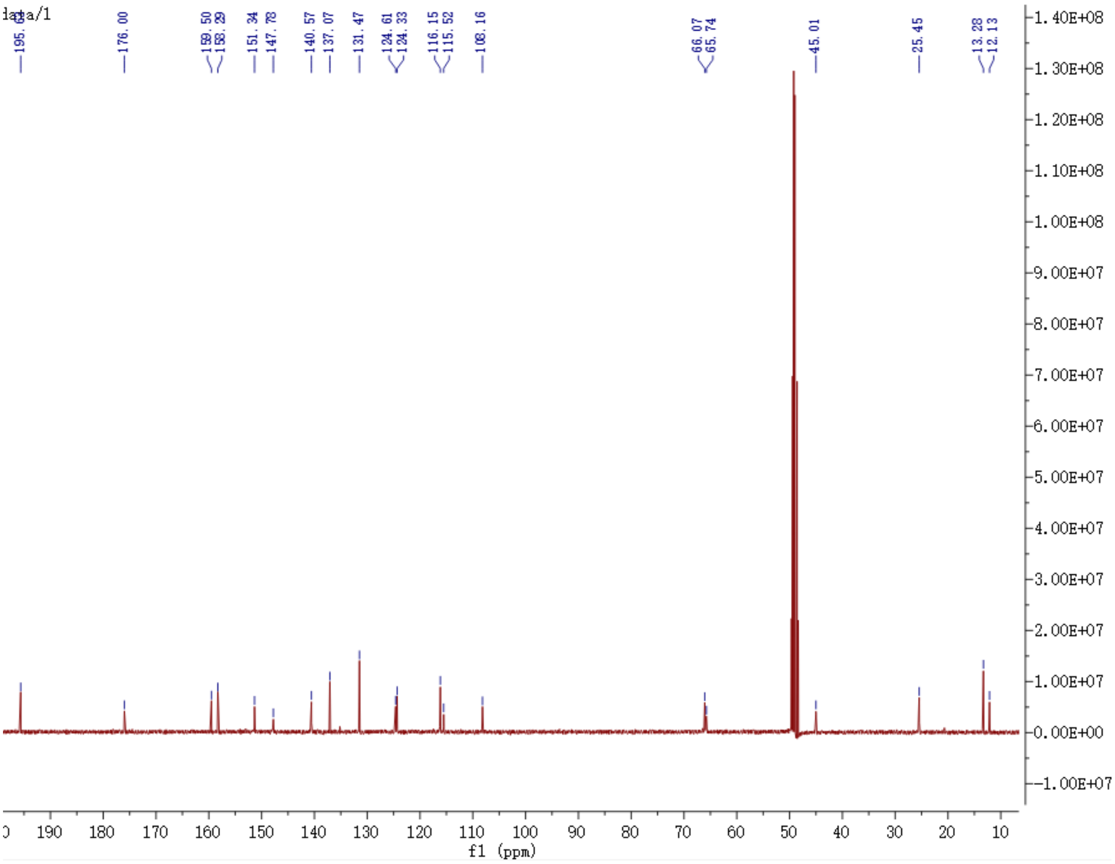

^{13}C NMR spectrum of 1-O-methyl-15-hydroxytenellin **(4)** (100 Hz, CD3OD-d_4).

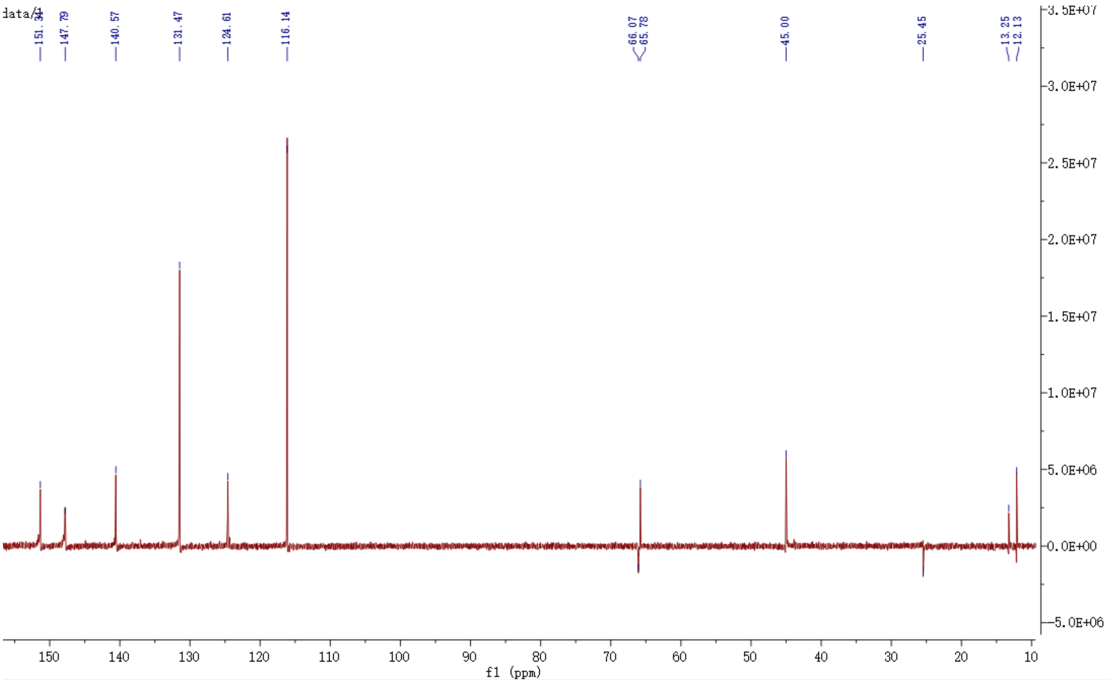

DEPT spectrum of 1-O-methyl-15-hydroxytenellin **(4)** (100 Hz, CD3OD-*d*4).

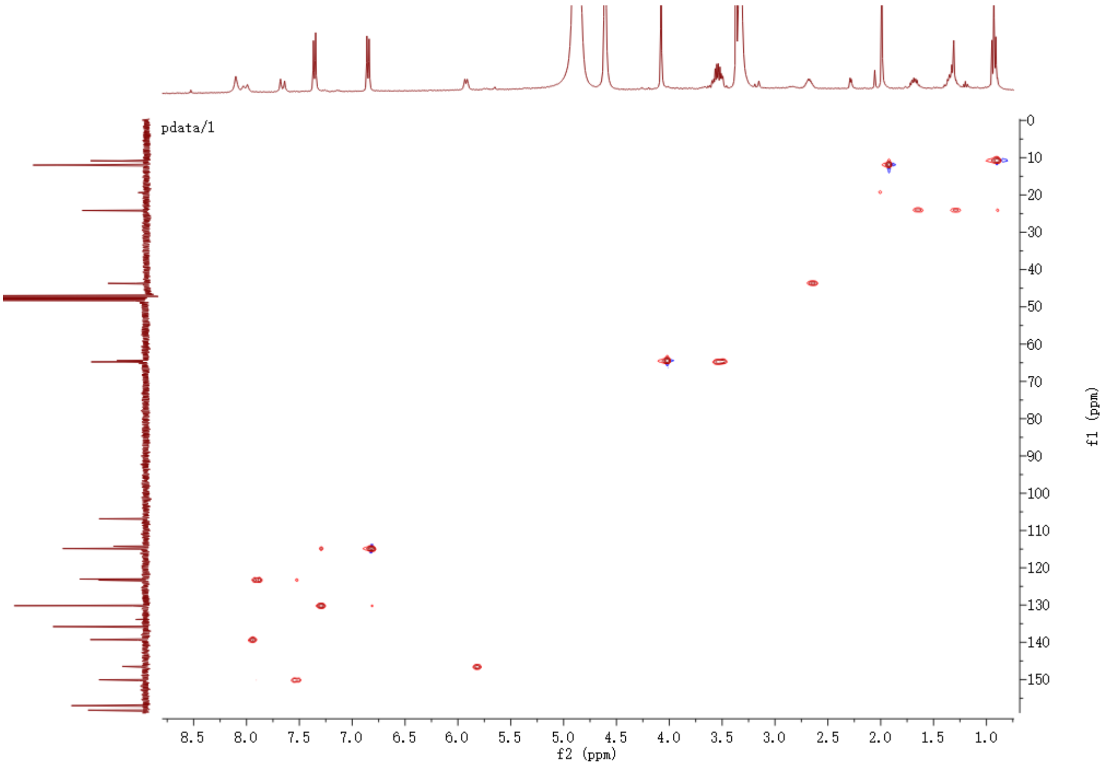

HSQC spectrum of 1-O-methyl-15-hydroxytenellin (**4**).

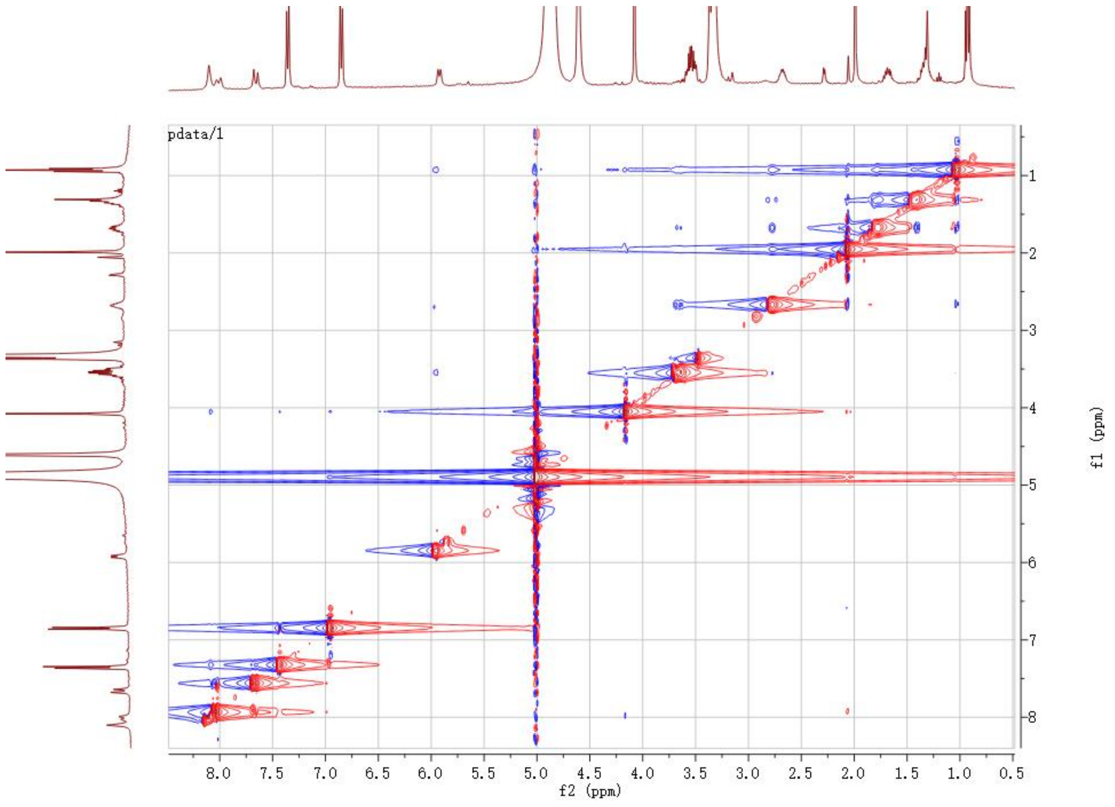

^{1}H-^{1}H NOESY spectrum of 1-O-methyl-15-hydroxytenellin (**4**).

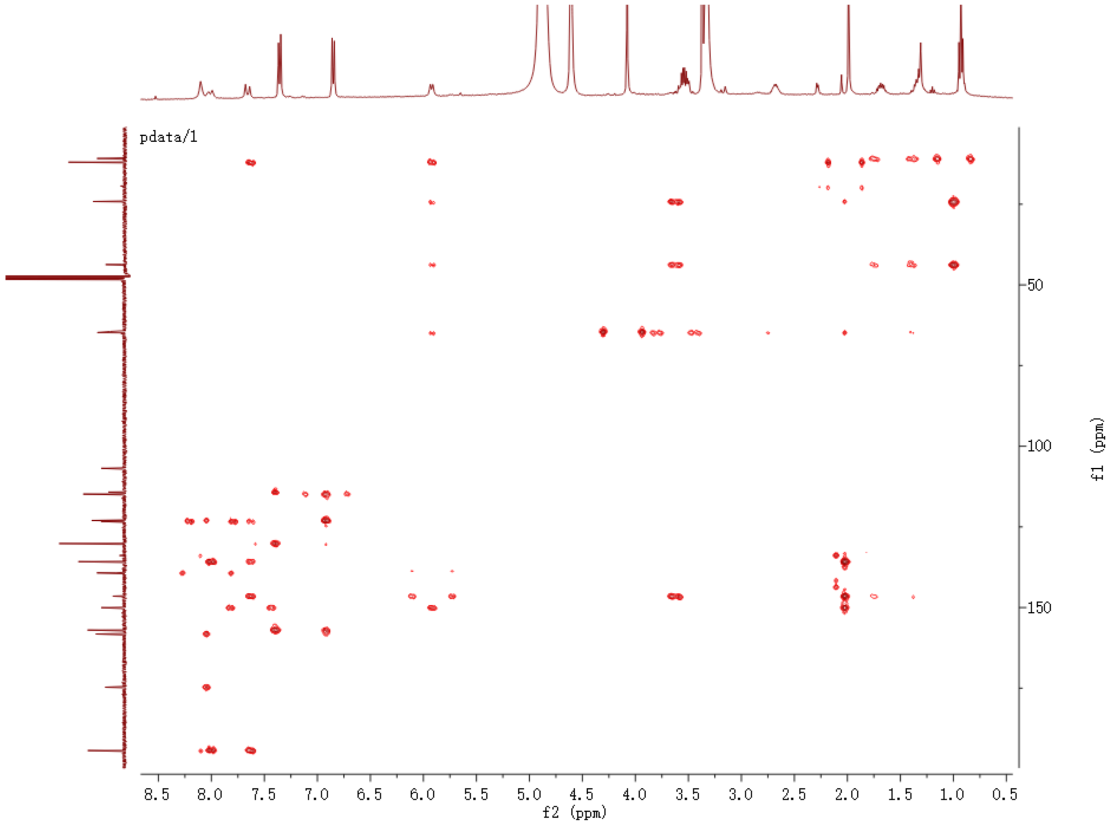

HMBC spectrum of 1-O-methyl-15-hydroxytenellin (**4**).

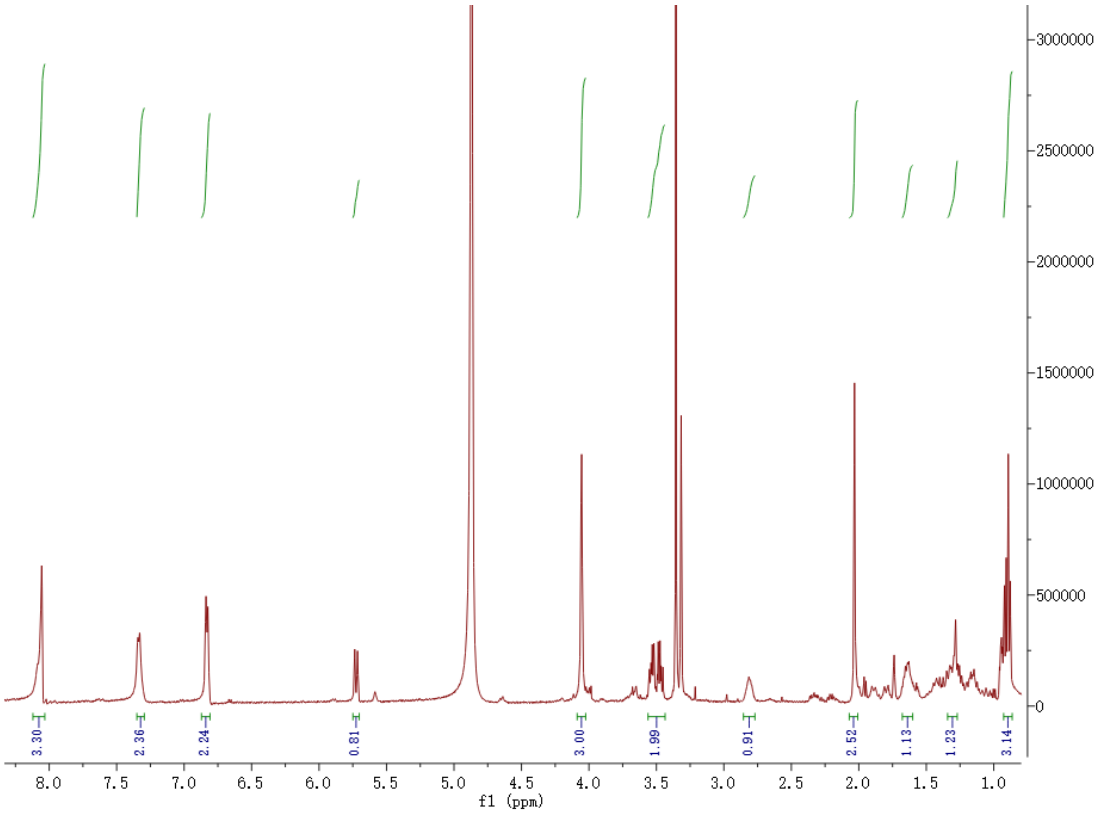

^{1}H NMR spectrum of (10*Z*)-1-O-methyl-15-hydroxytenellin **(5)** (500 Hz, CD3OD-*d*$_4$).

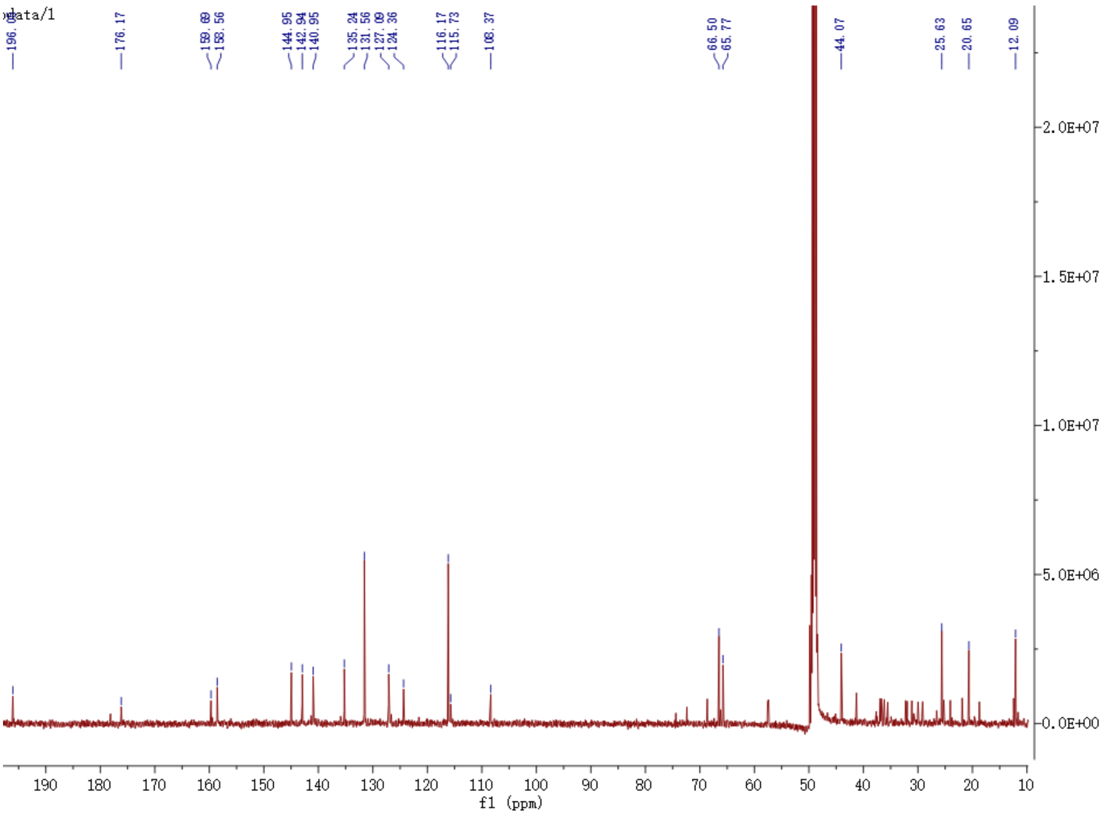

^{13}C NMR spectrum of (10*Z*)-1-O-methyl-15-hydroxytenellin **(5)** (100 Hz, CD3OD-*d*$_4$).

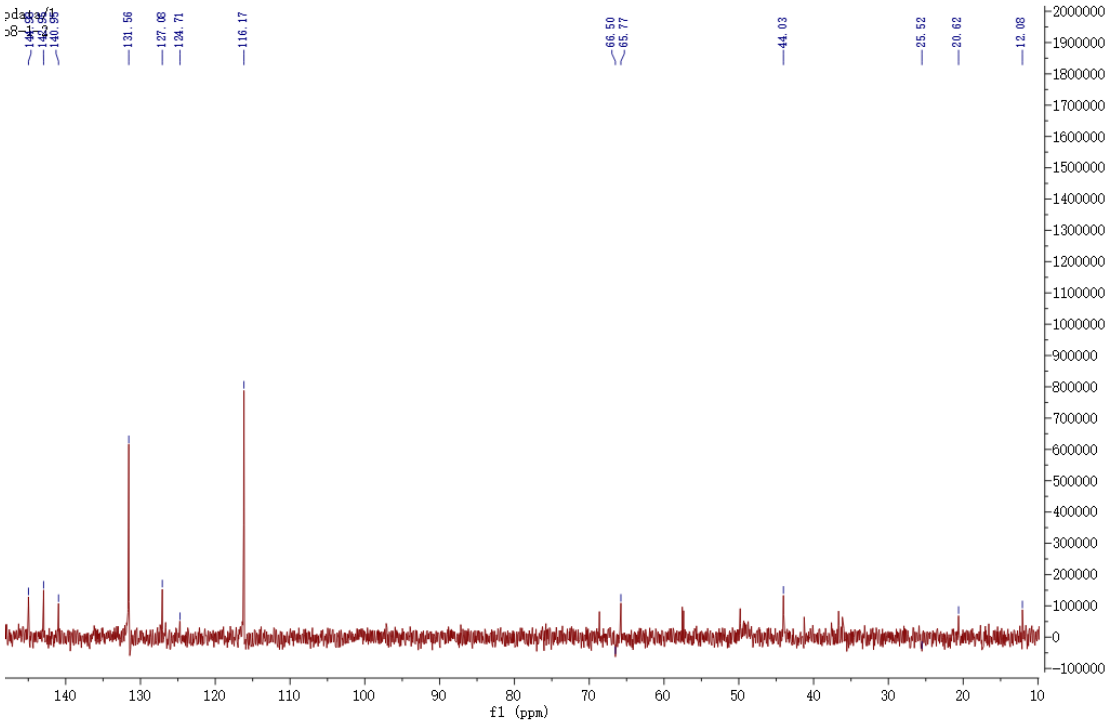

DEPT spectrum of (10*Z*)-1-O-methyl-15-hydroxytenellin **(5)** (100 Hz, CD3OD-d_4).

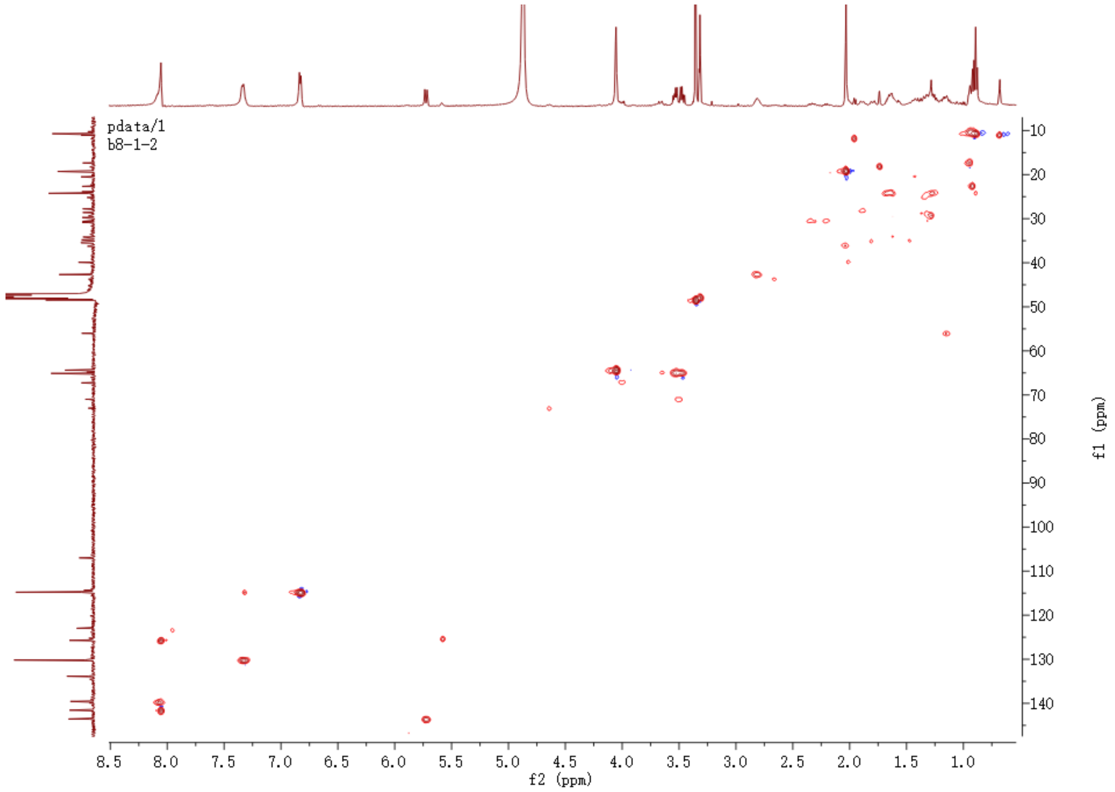

HSQC spectrum of (10*Z*)-1-O-methyl-15-hydroxytenellin (**5**).

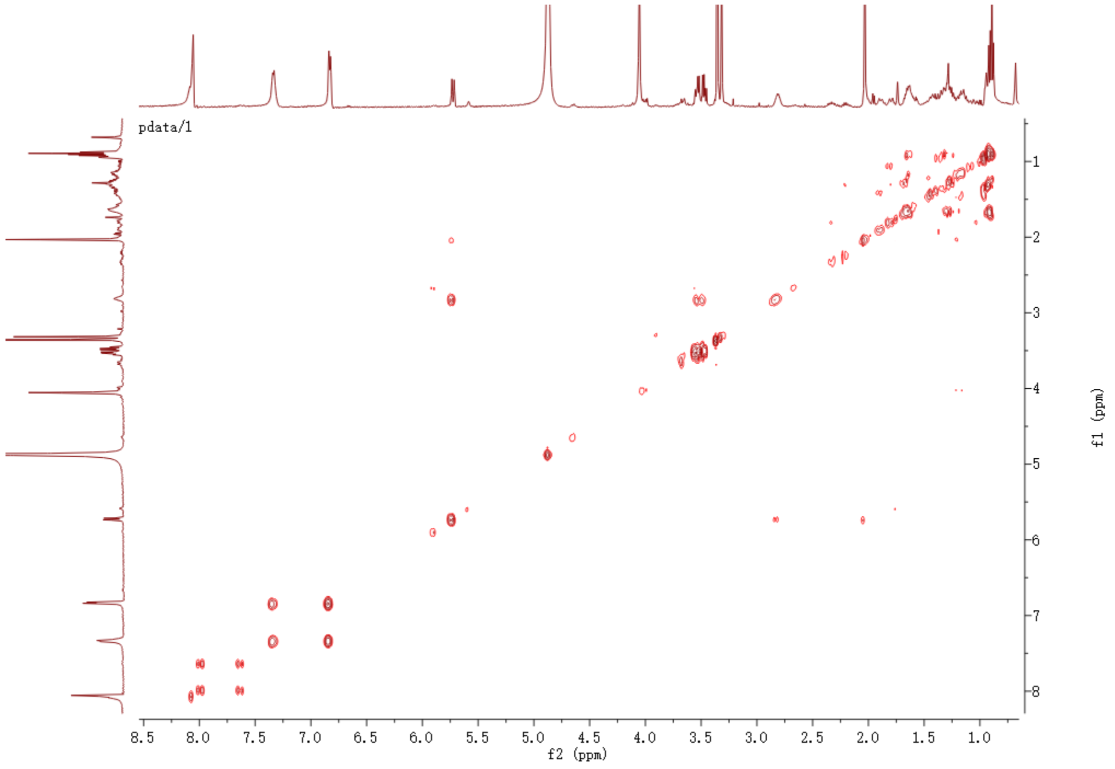

^{1}H-^{1}H COSY spectrum of (10*Z*)-1-*O*-methyl-15-hydroxytenellin **(5)** (500 Hz, CD3OD-*d*$_4$).

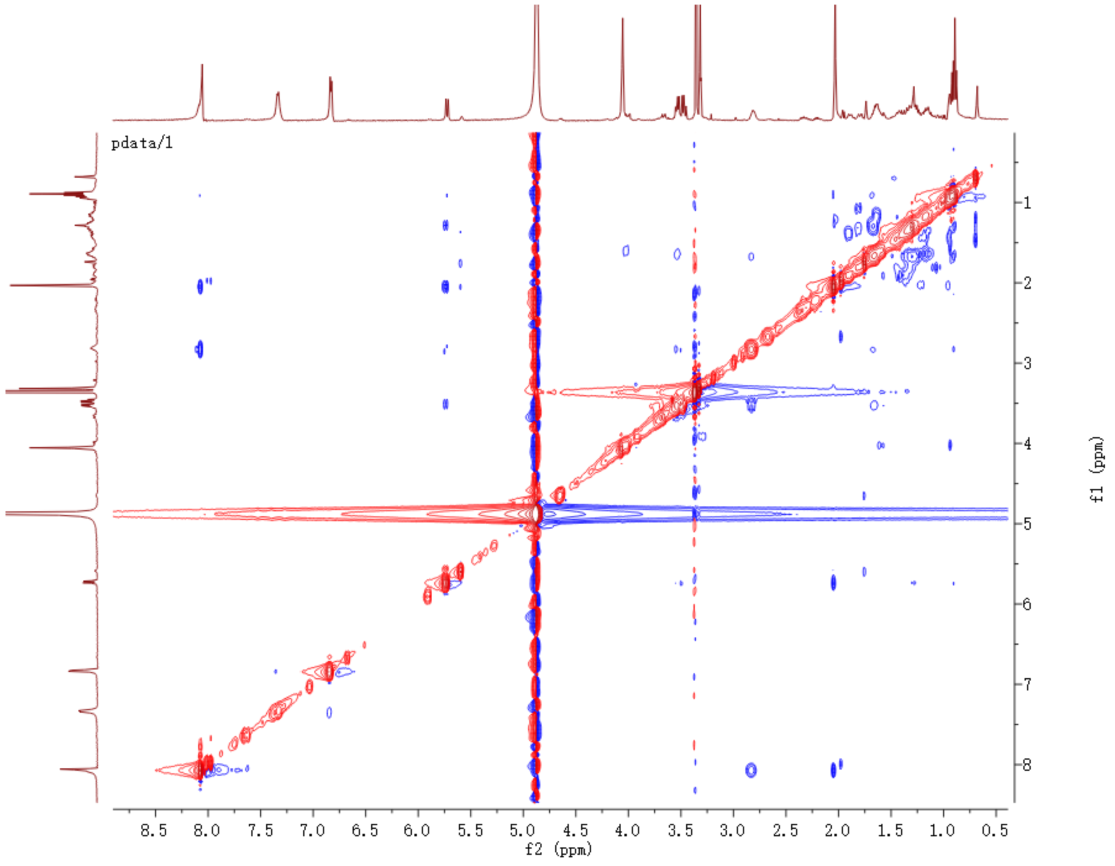

^{1}H-^{1}H NOESY spectrum of (10*Z*)-1-O-methyl-15-hydroxytenellin **(5)** (500 Hz, CD3OD-*d*$_4$).

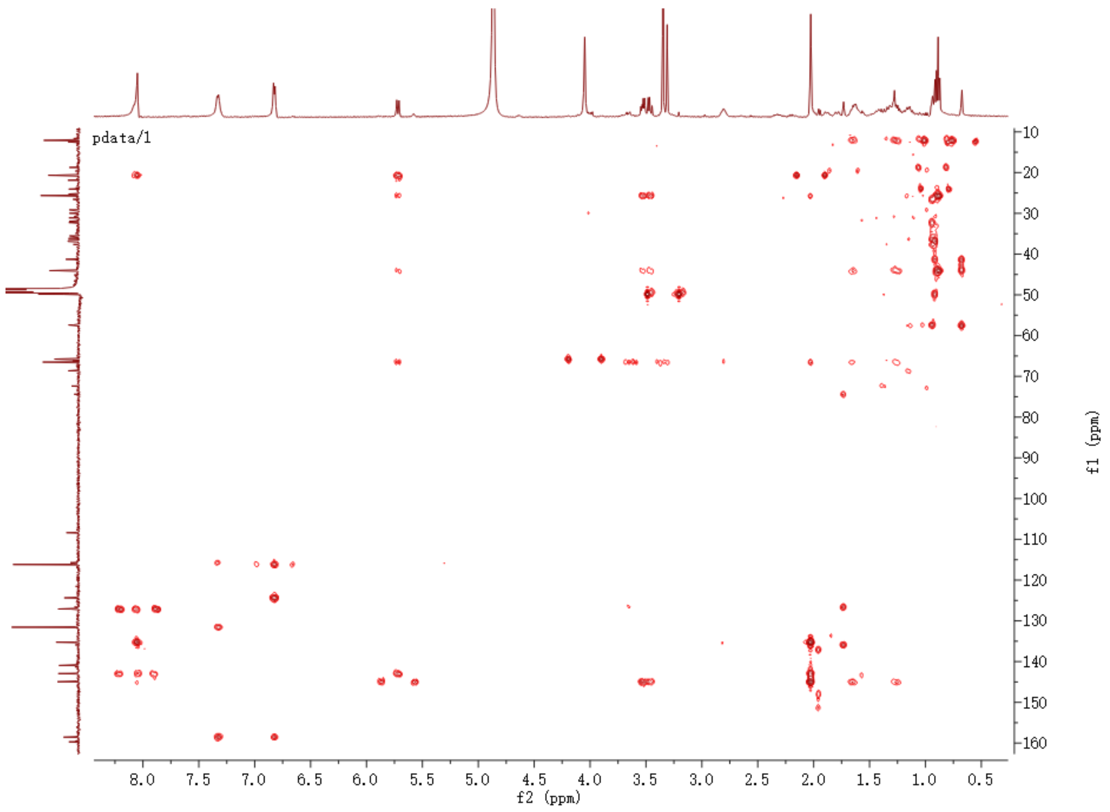

HMBC spectrum of (10*Z*)-1-O-methyl-15-hydroxytenellin (**5**).

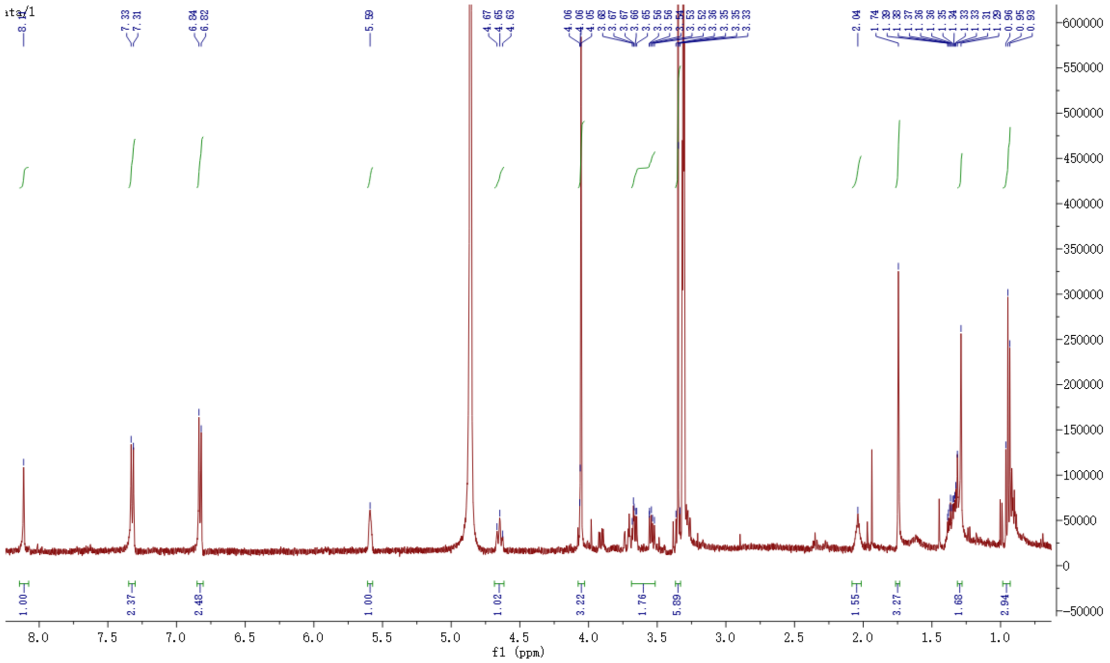

^{1}H NMR spectrum of *O*-methyltenellin A **(6)** (500 Hz, CD_3OD-d_4).

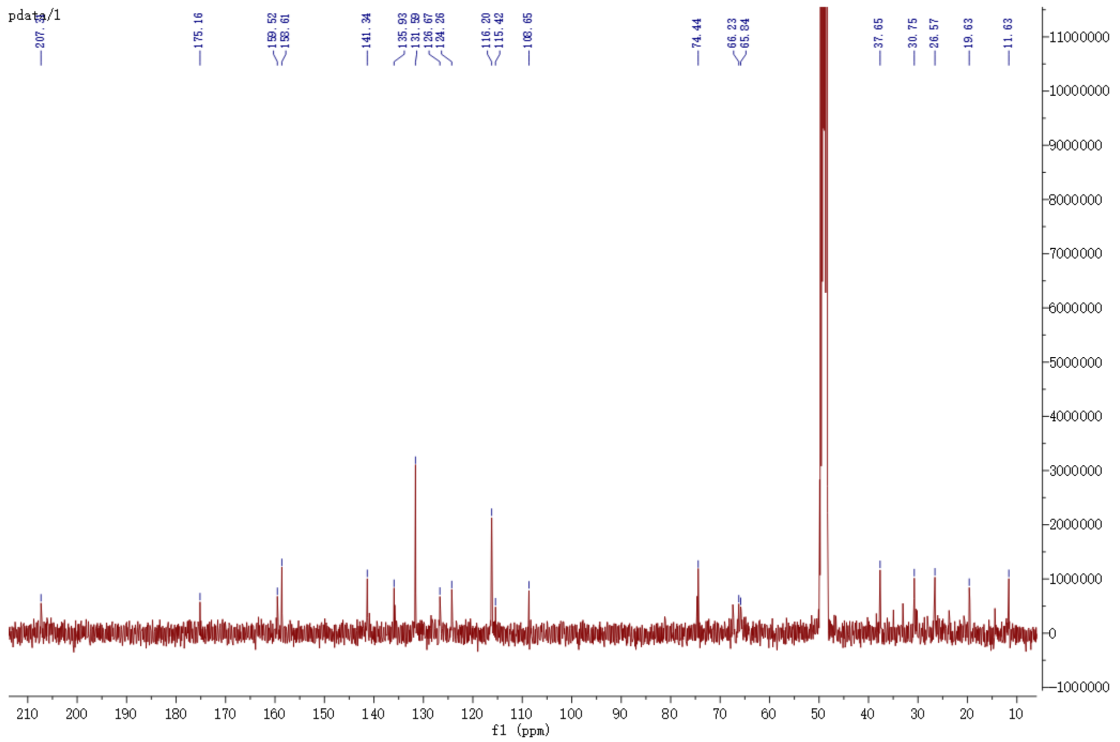

^{13}C NMR spectrum of *O*-methyltenellin A **(6)** (100 Hz, CD_3OD-d_4).

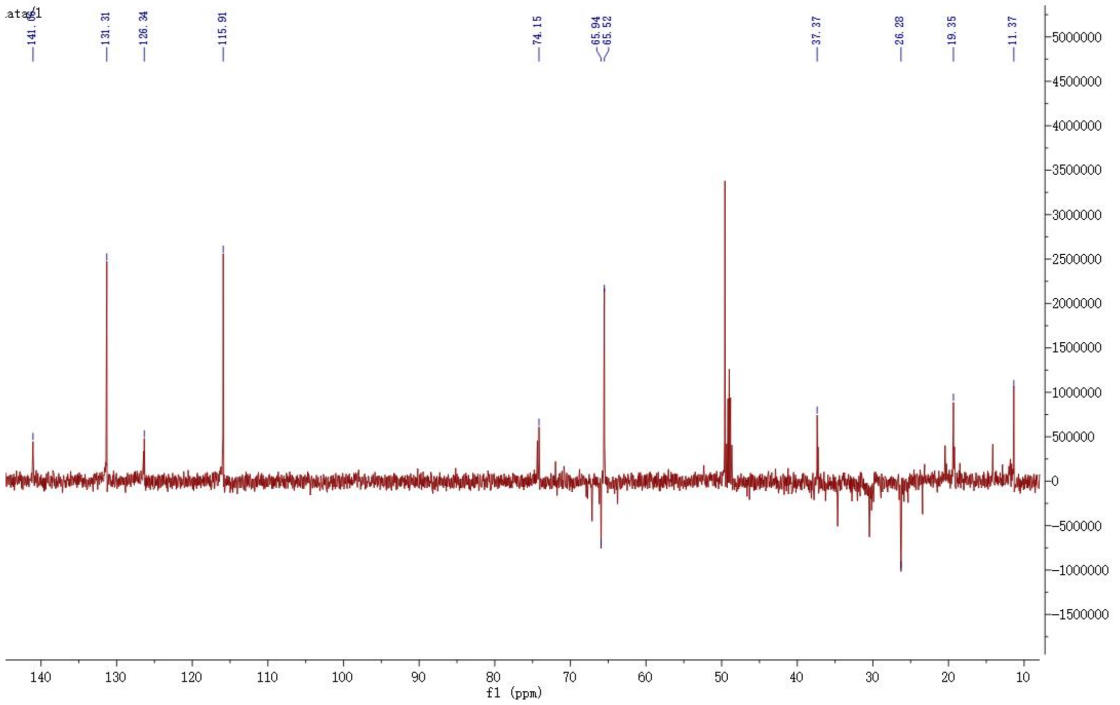

DEPT spectrum of *O*-methyltenellin A (**6**) (100 Hz, CD_3OD-d_4).

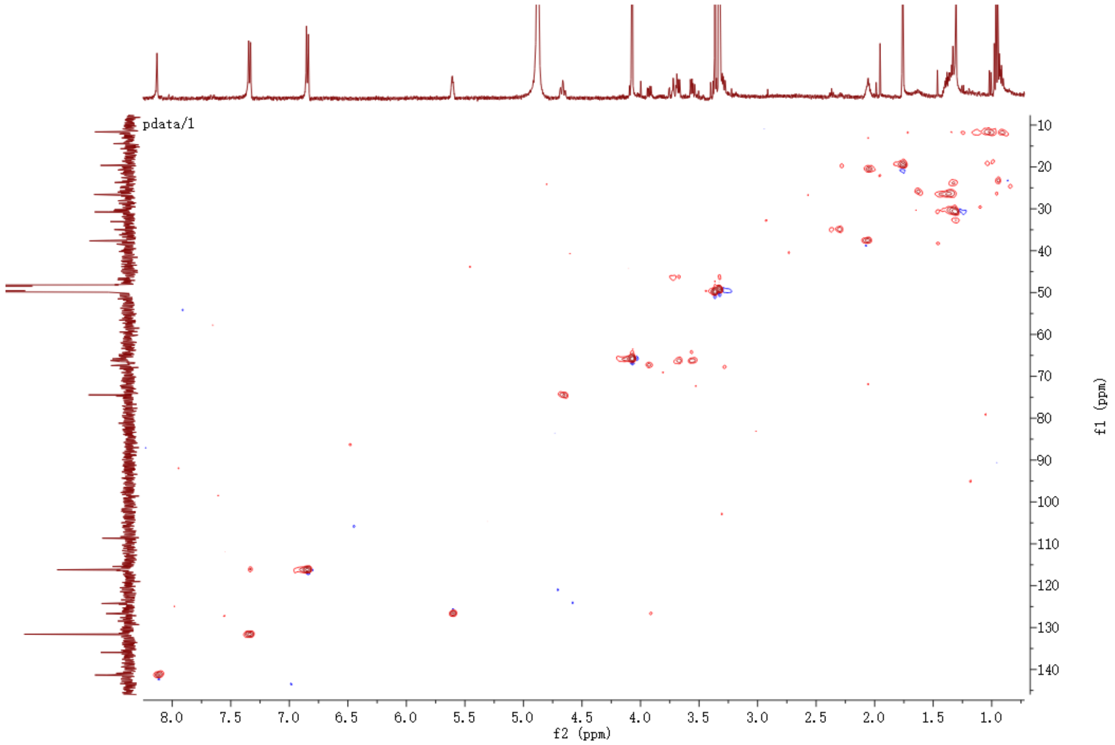

HSQC spectrum of *O*-methyltenellin A (**6**).

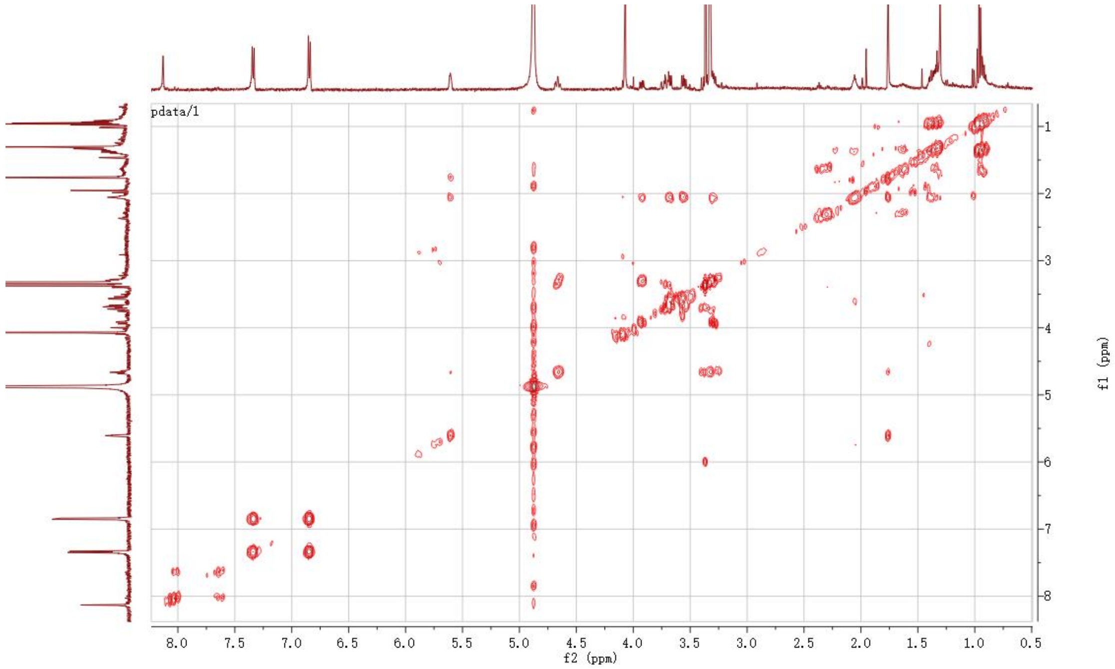

^{1}H-^{1}H COSY spectrum of *O*-methyltenellin A (**6**) (500 Hz, CD_3OD-*d_4*).

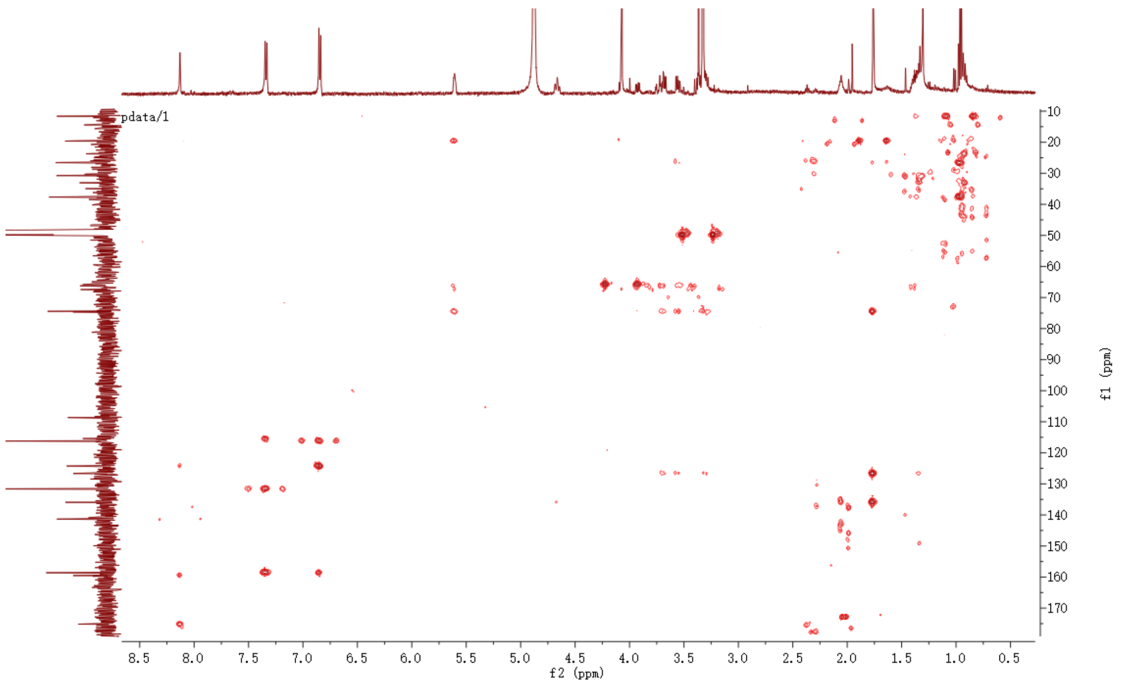

HMBC spectrum of *O*-methyltenellin A (**6**).

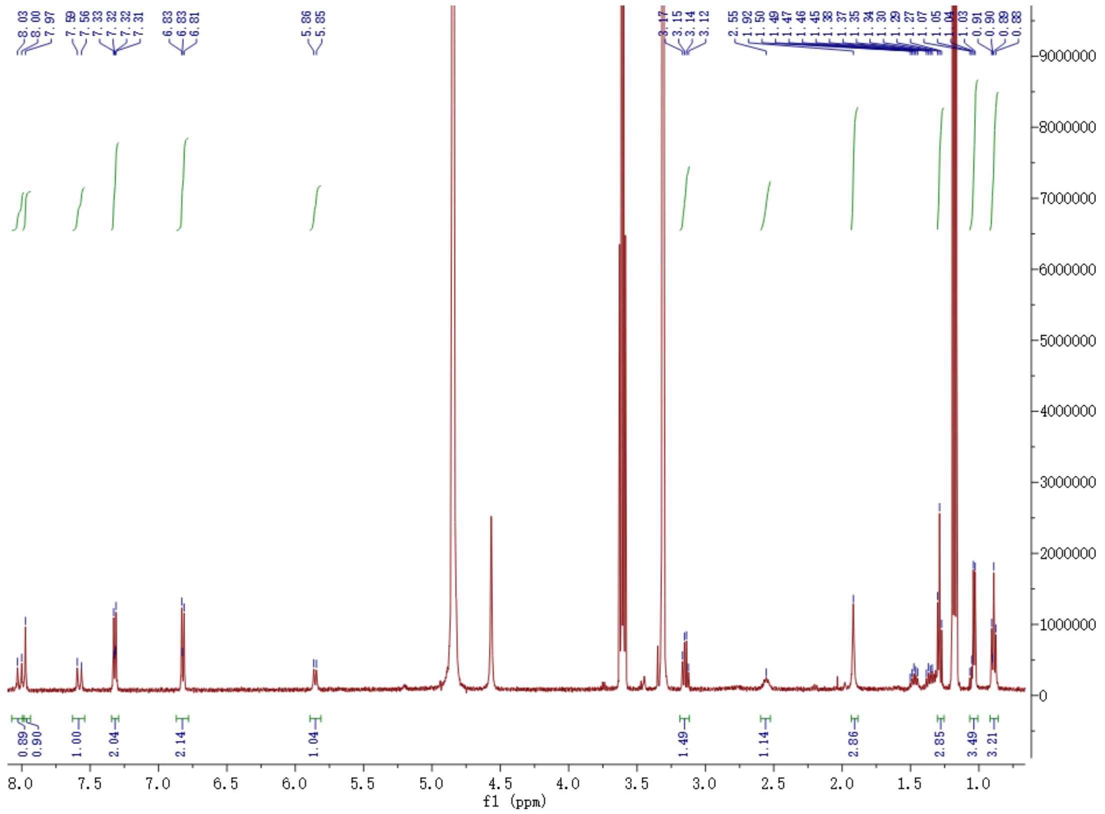

^{1}H NMR spectrum of tenellin (**7**) (500 Hz, CD3OD-d_4).

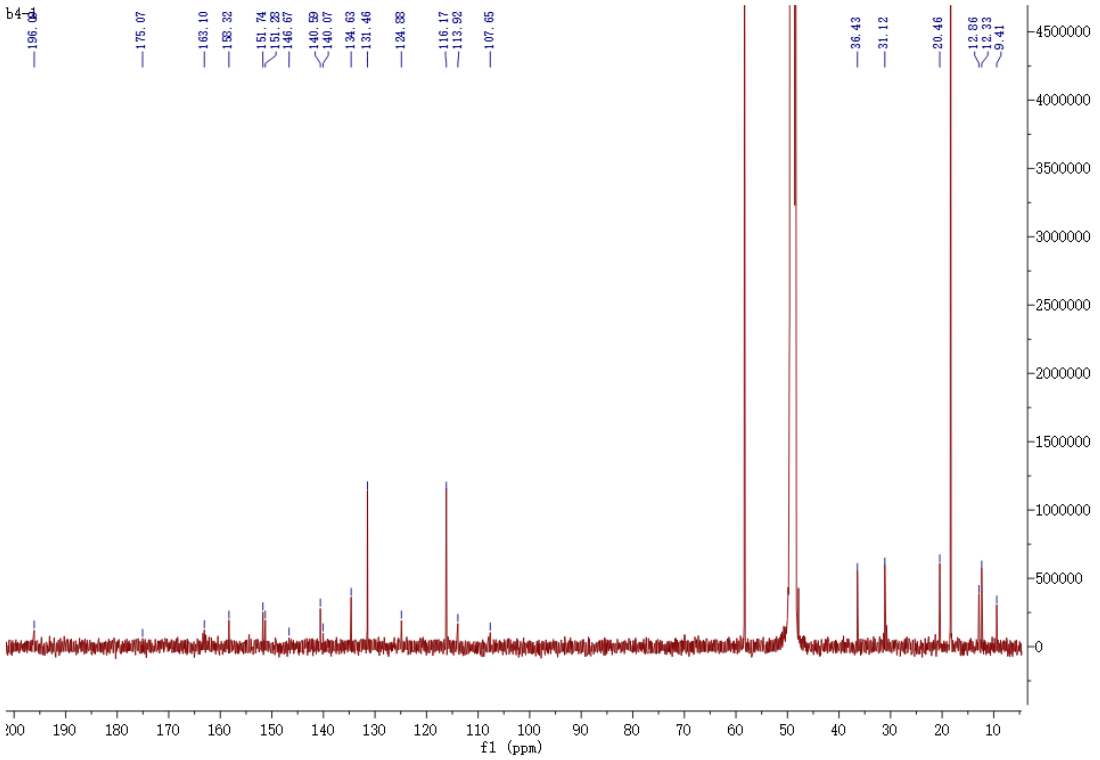

^{13}C NMR spectrum of tenellin (**7**) (100 Hz, CD3OD-*d*$_4$).

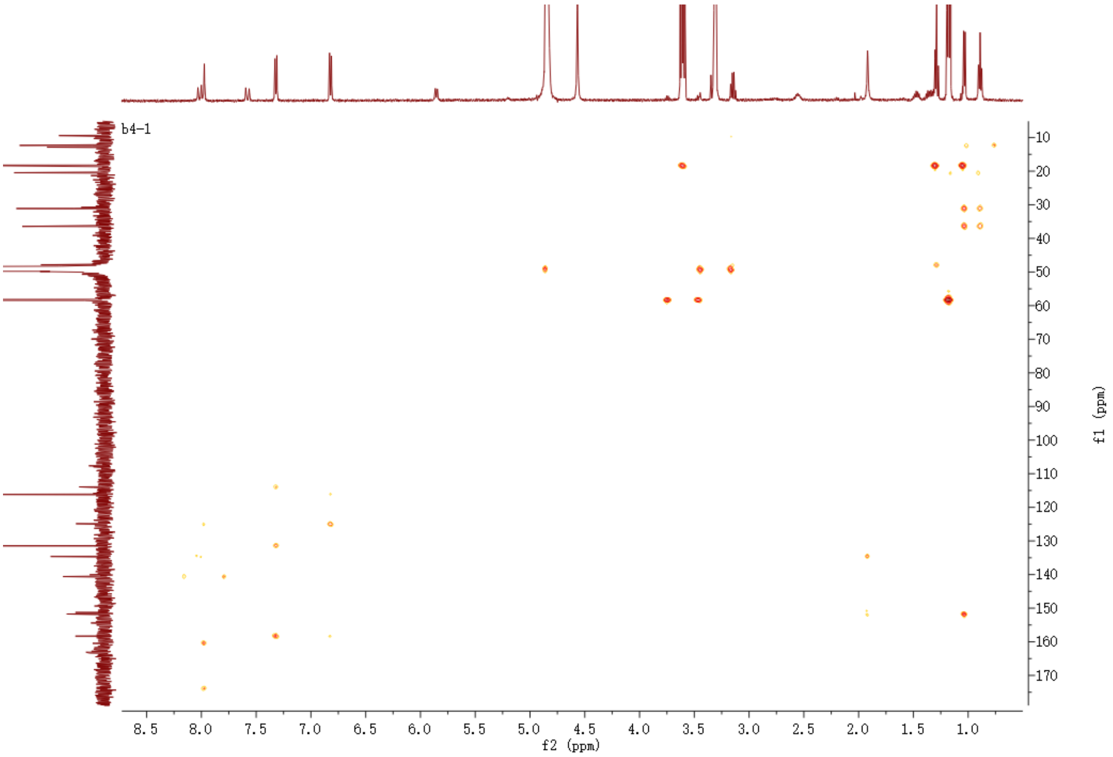

HMBC spectrum of tenellin (**7**).

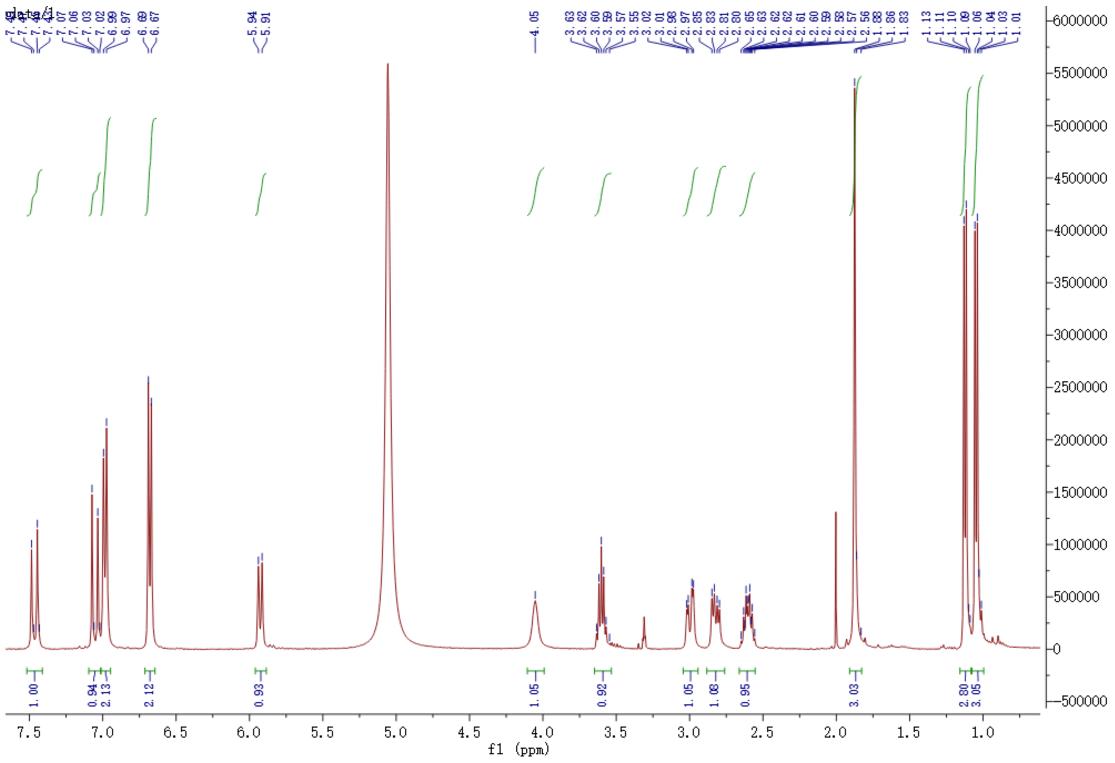

^{1}H NMR spectrum of 12-hydropretenellin A (**8**) (400 Hz, CD3OD-d_4).

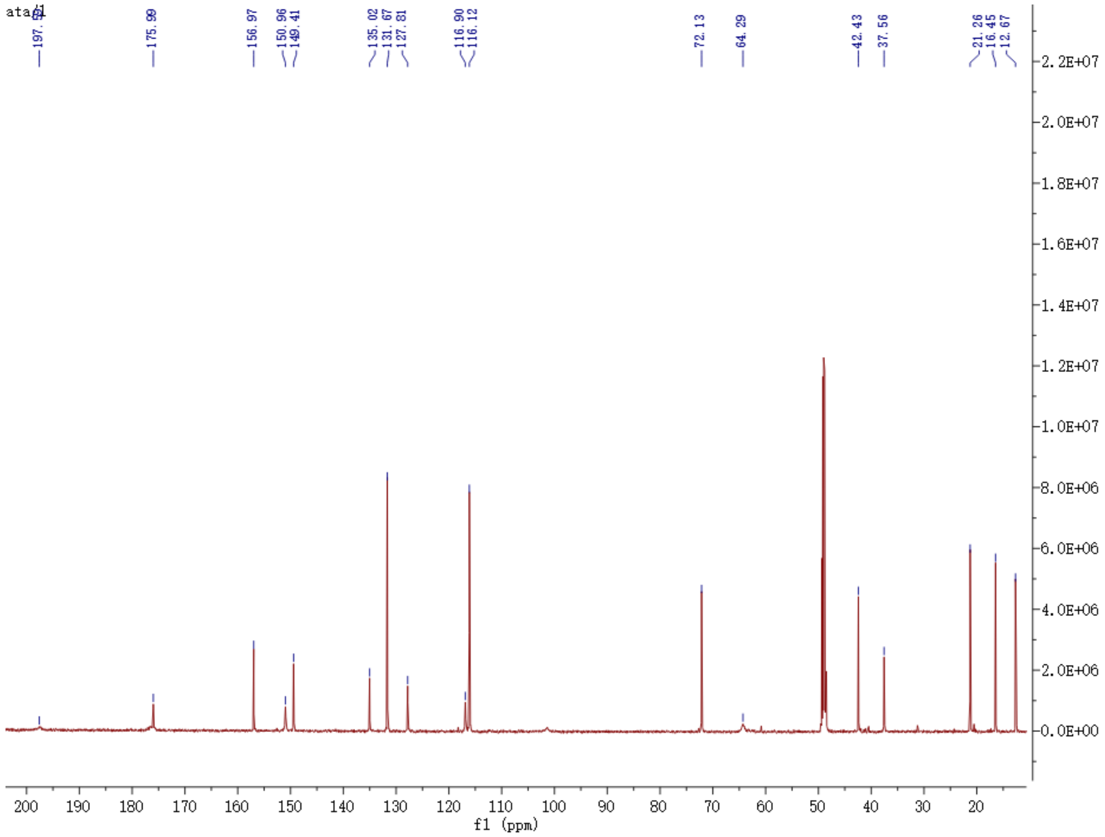

^{13}C NMR spectrum of 12-hydropretenellin A (**8**) (100 Hz, CD3OD-d_4).

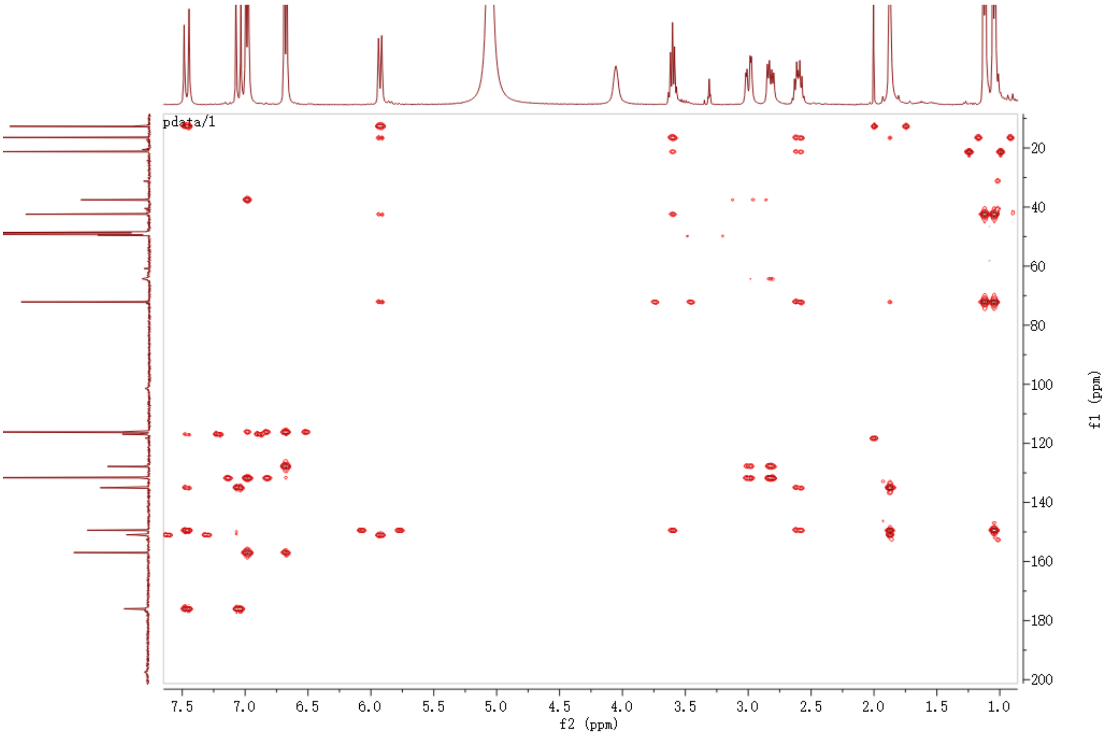

HMBC spectrum of 12-hydropretenellin A (**8**).

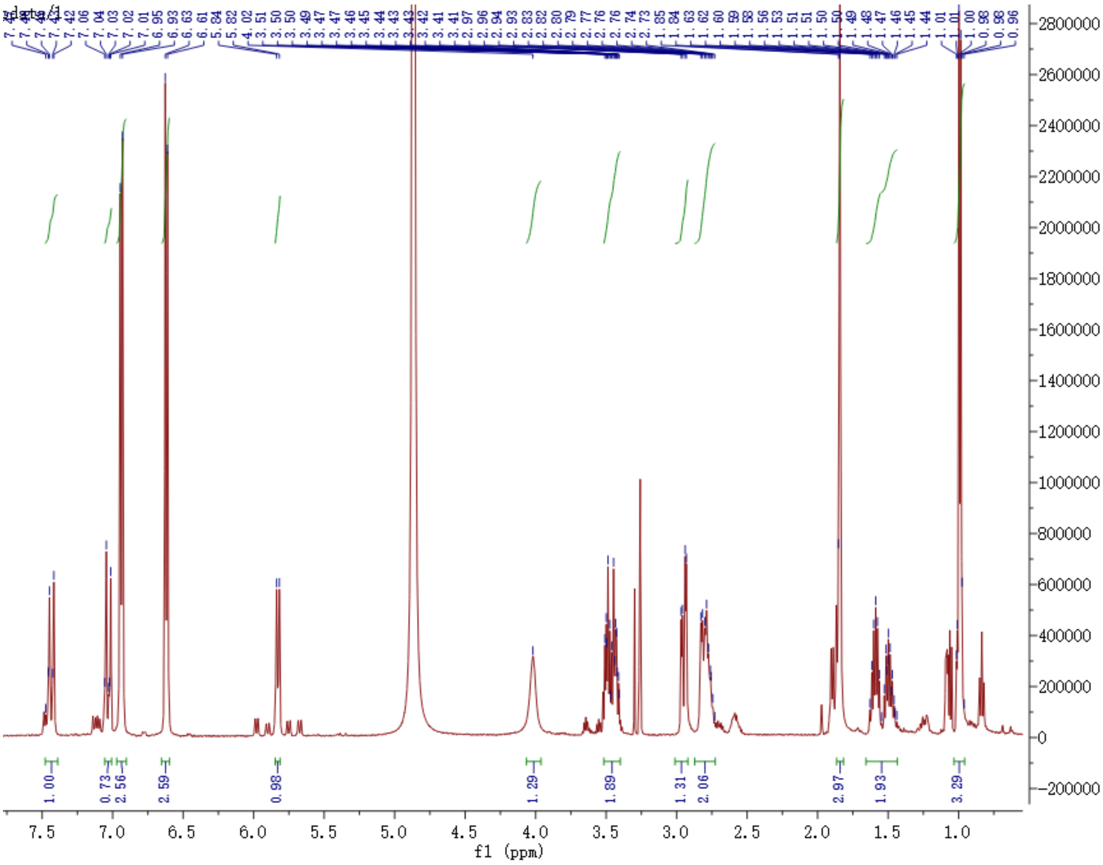

^{1}H NMR spectrum of 13-hydropretenellin A (**9**) (500 Hz, CD3OD-d_4).

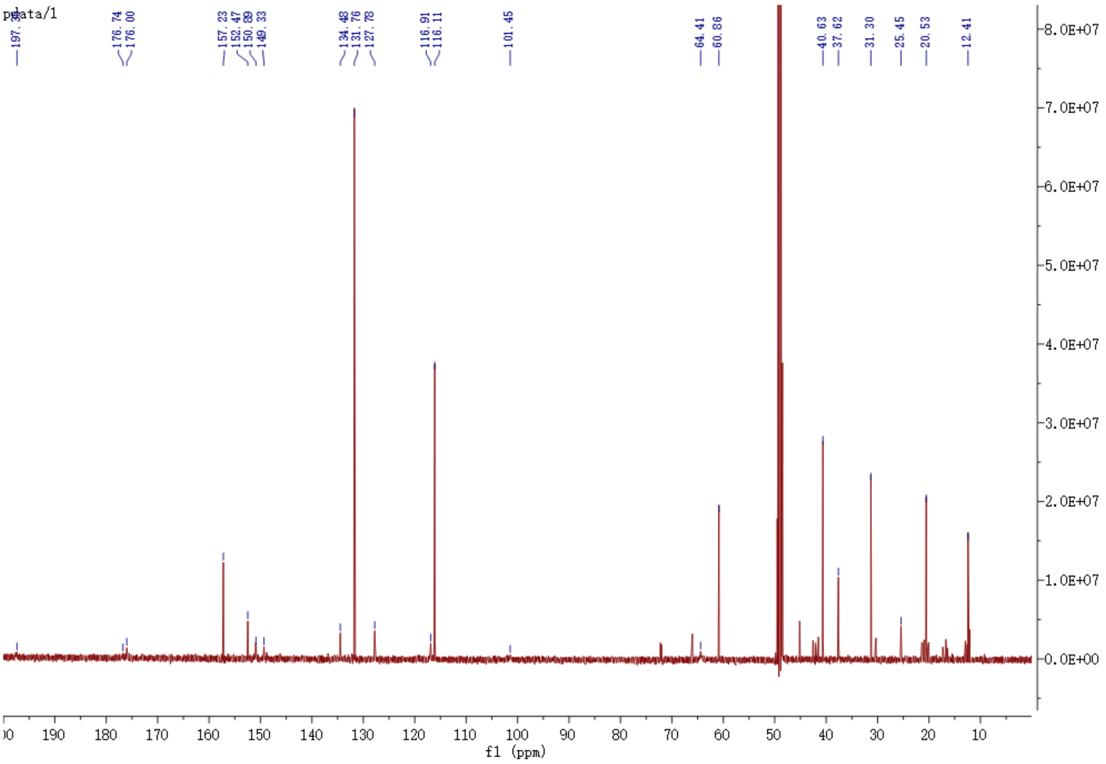

^{13}C NMR spectrum of 13-hydropretenellin A (**9**) (100 Hz, CD3OD-*d$_4$*).

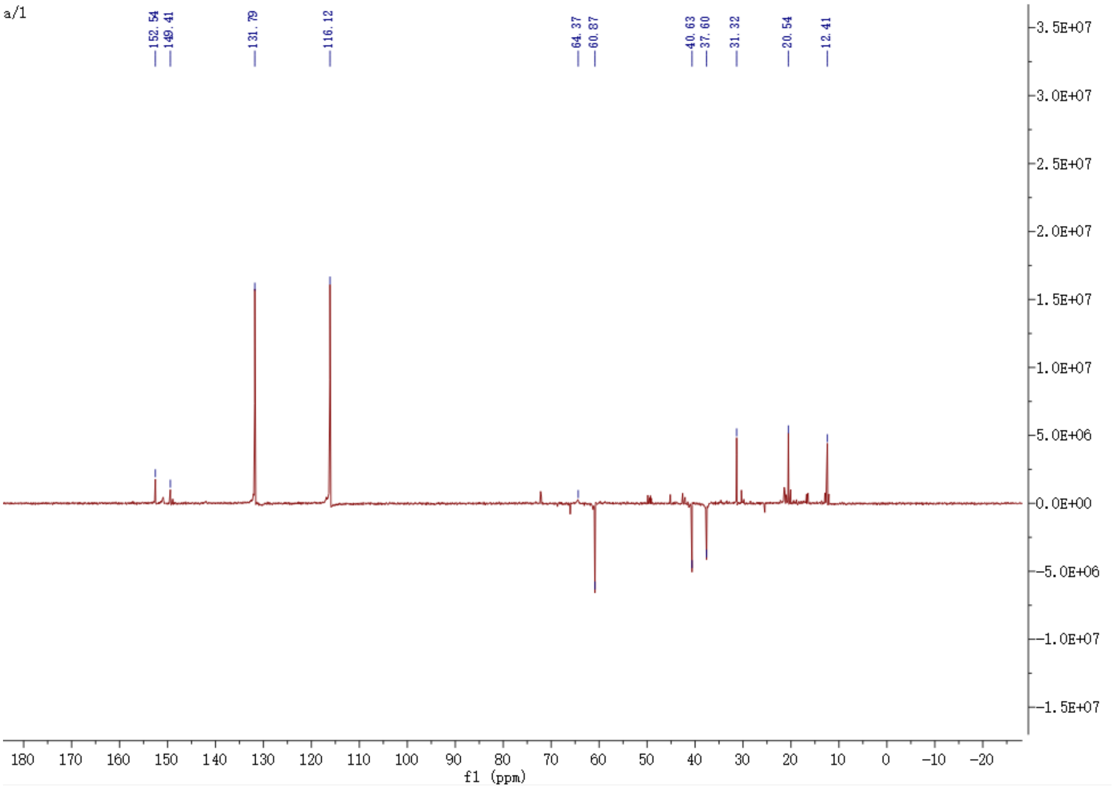

DEPT spectrum of 13-hydropretenellin A (**9**) (100 Hz, CD3OD-*d$_4$*).

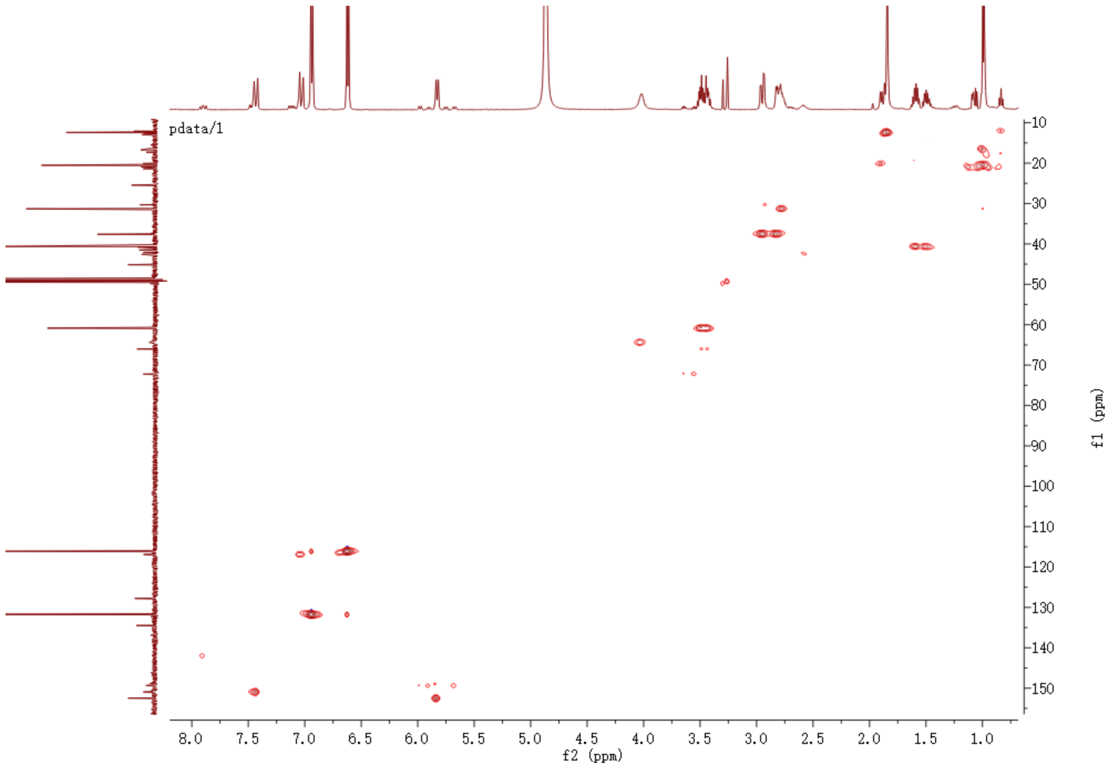

HSQC spectrum of 13-hydropretenellin A (**9**).

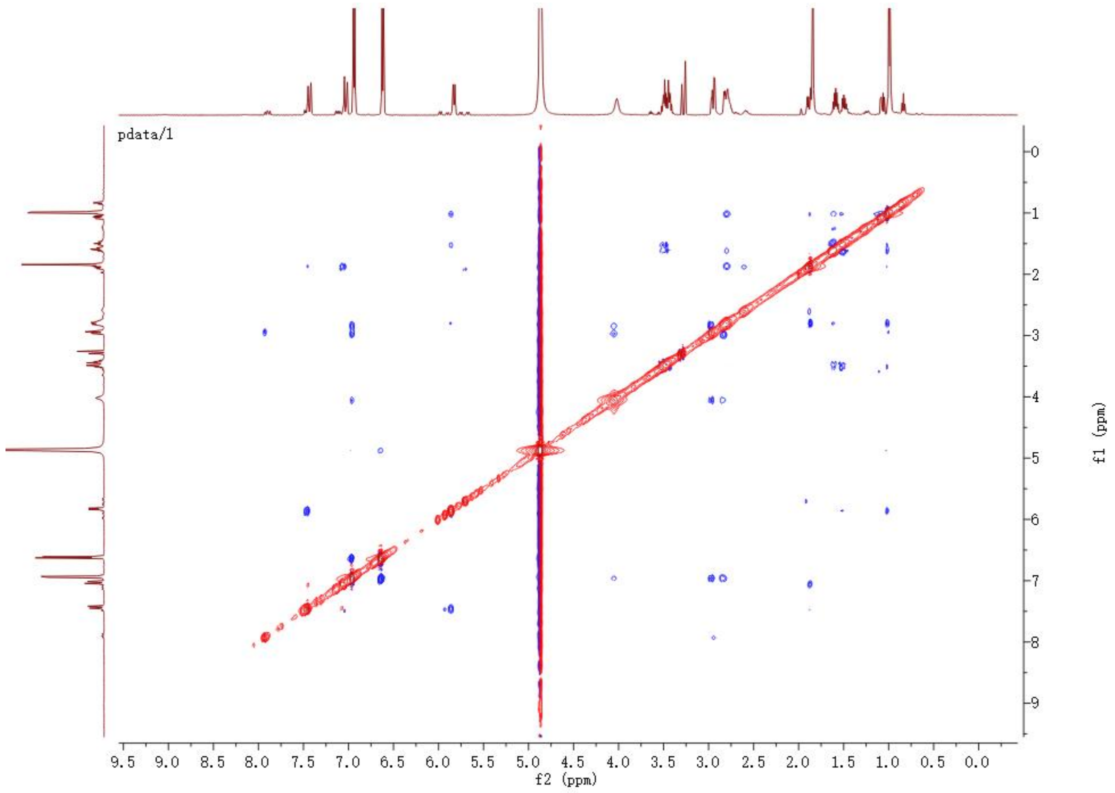

^{1}H-^{1}H COSY spectrum of 13-hydropretenellin A (**9**) (500 Hz, CD3OD-d_4).

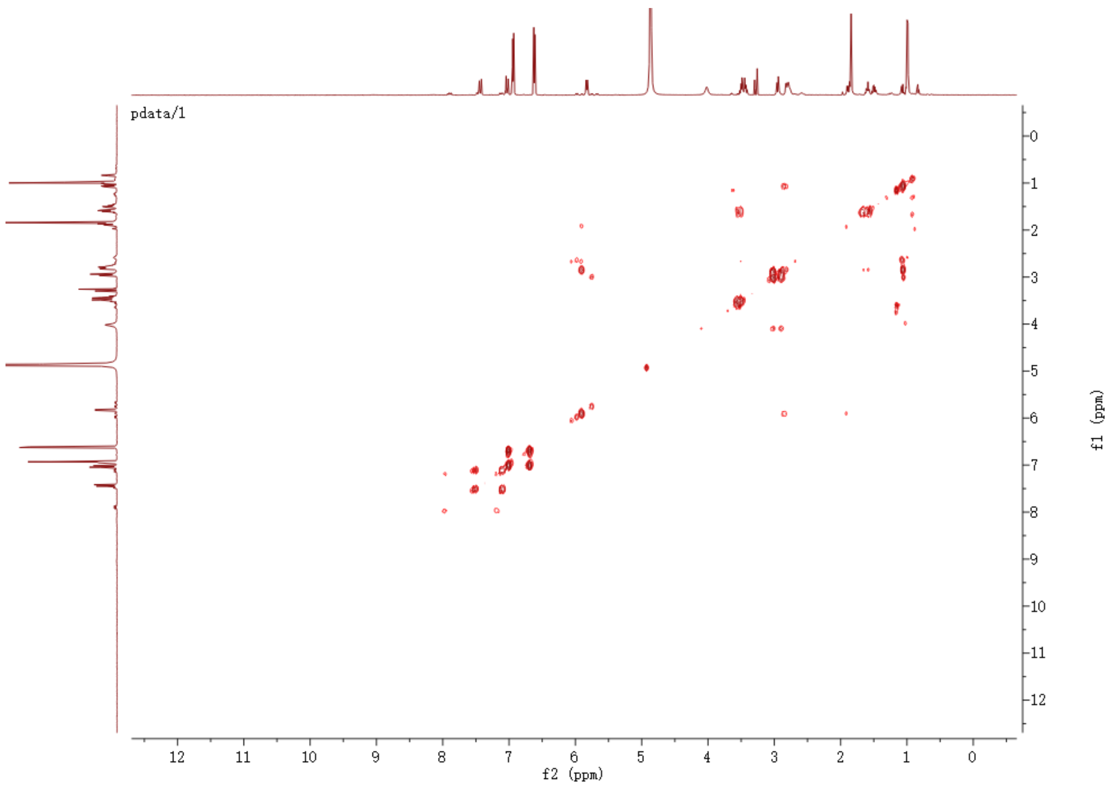

^{1}H-^{1}H NOESY spectrum of 13-hydropretenellin A (**9**) (500 Hz, CD3OD-*d*$_4$).

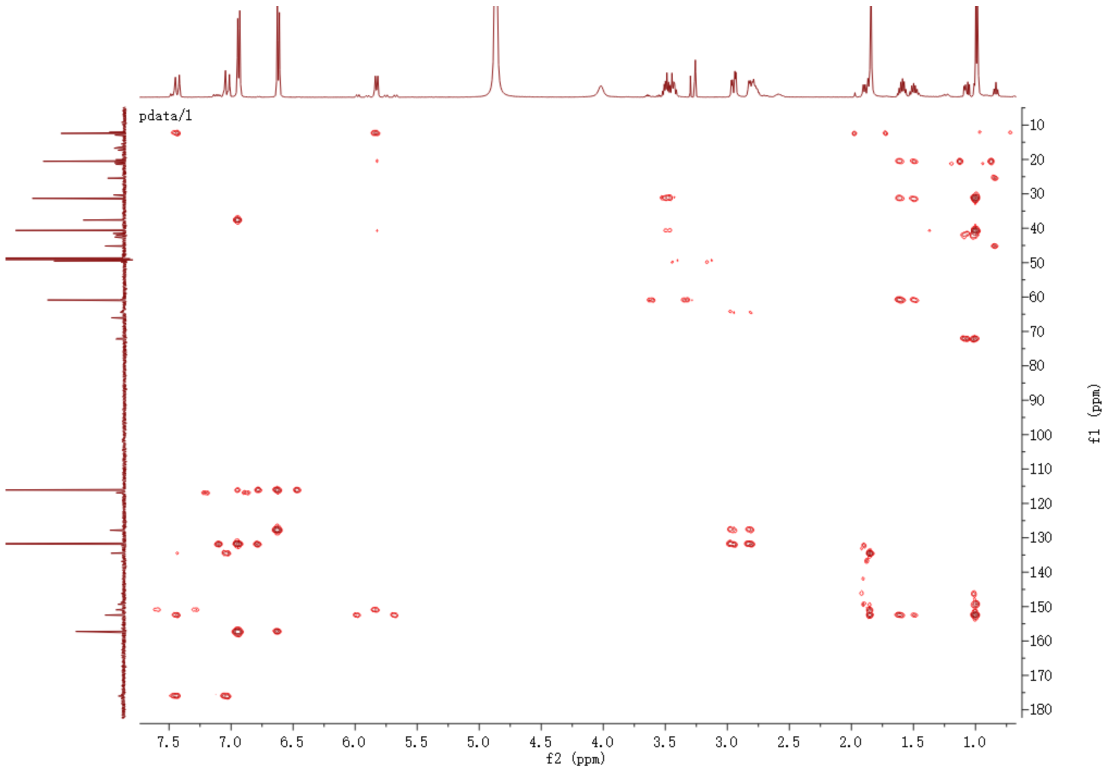

HMBC spectrum of 13-hydropretenellin A (**9**).

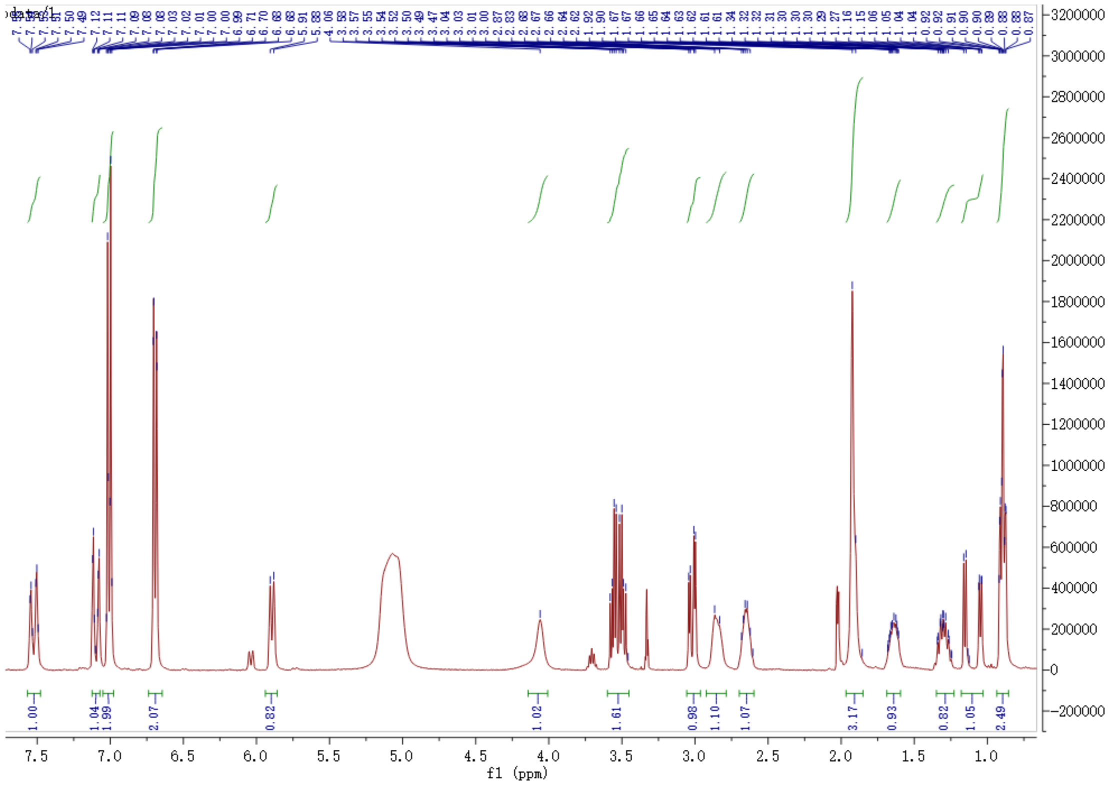

^{1}H NMR spectrum of 14-hydropretenellin A (**10**) (400 Hz, CD3OD-d_4).

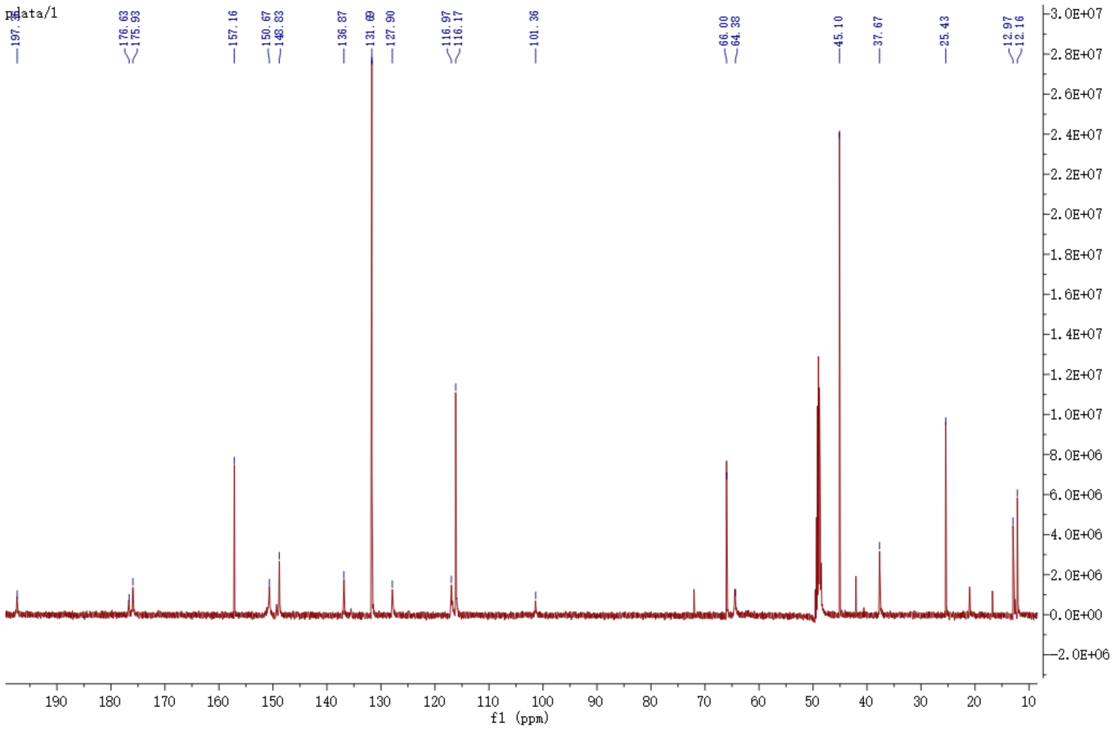

^{13}C NMR spectrum of 14-hydropretenellin A (**10**) (100 Hz, CD3OD-d_4).

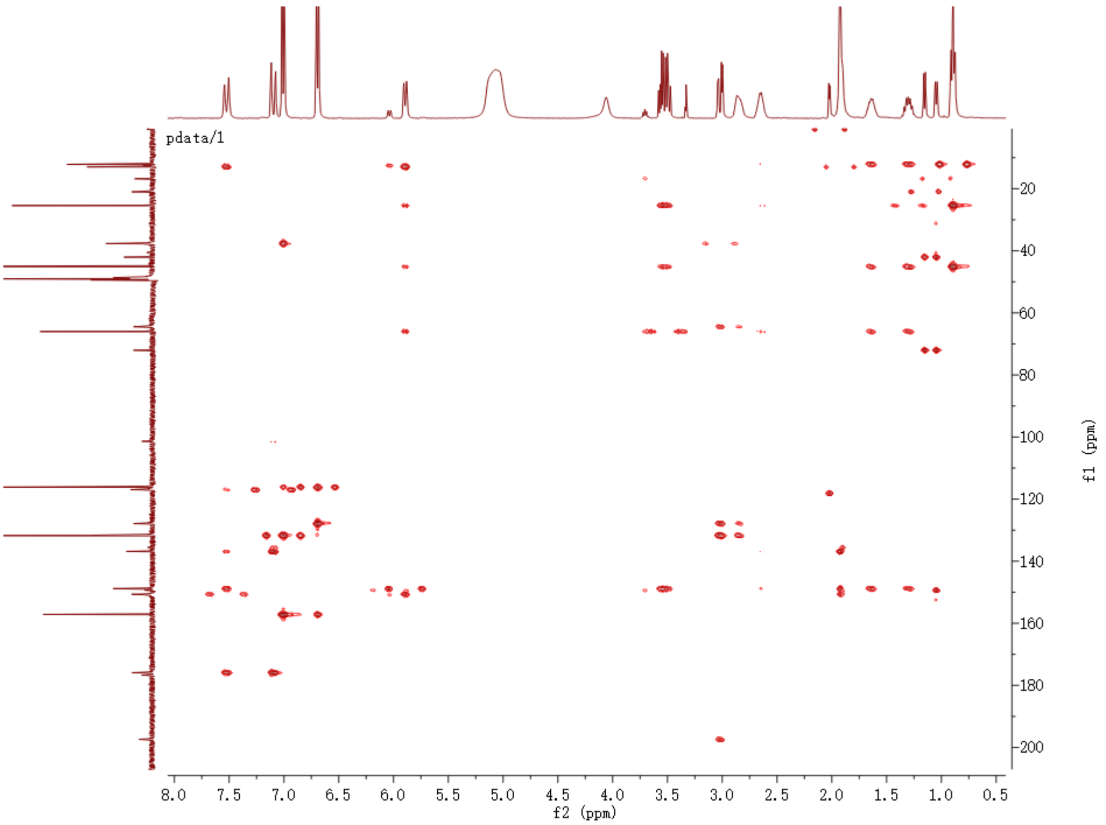

HMBC spectrum of 14-hydropretenellin A (**10**).

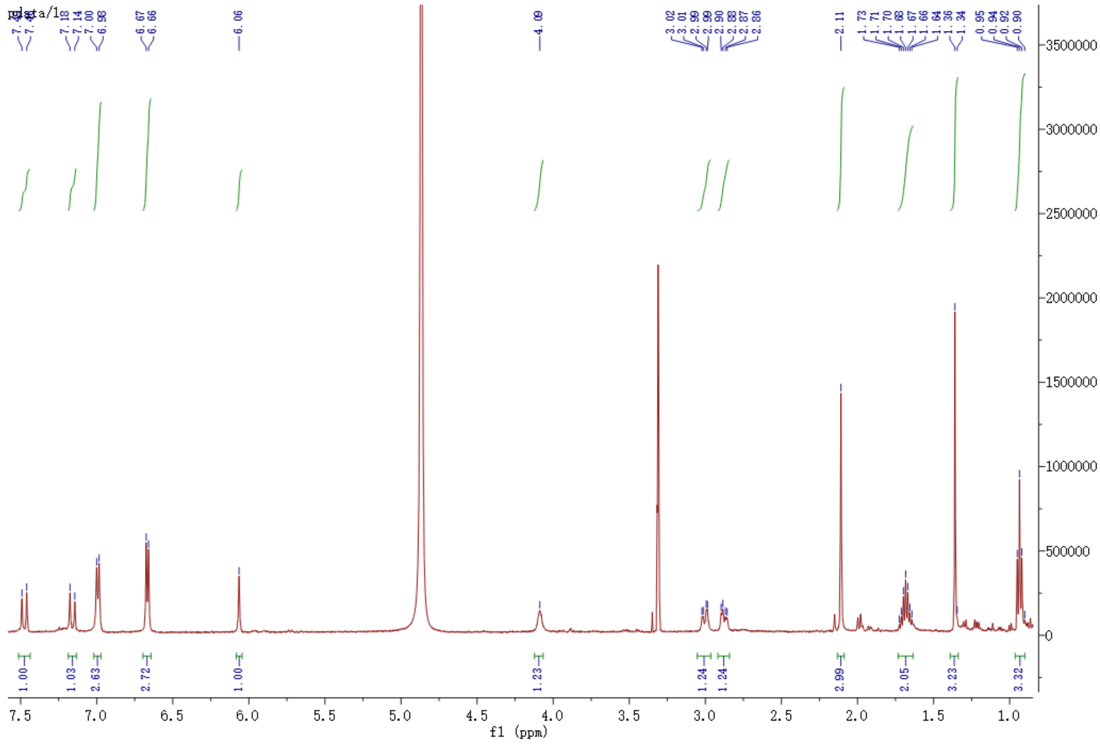

^{1}H NMR spectrum of 11-hydropretenellin A (**11**) (500 Hz, CD3OD-d_4).

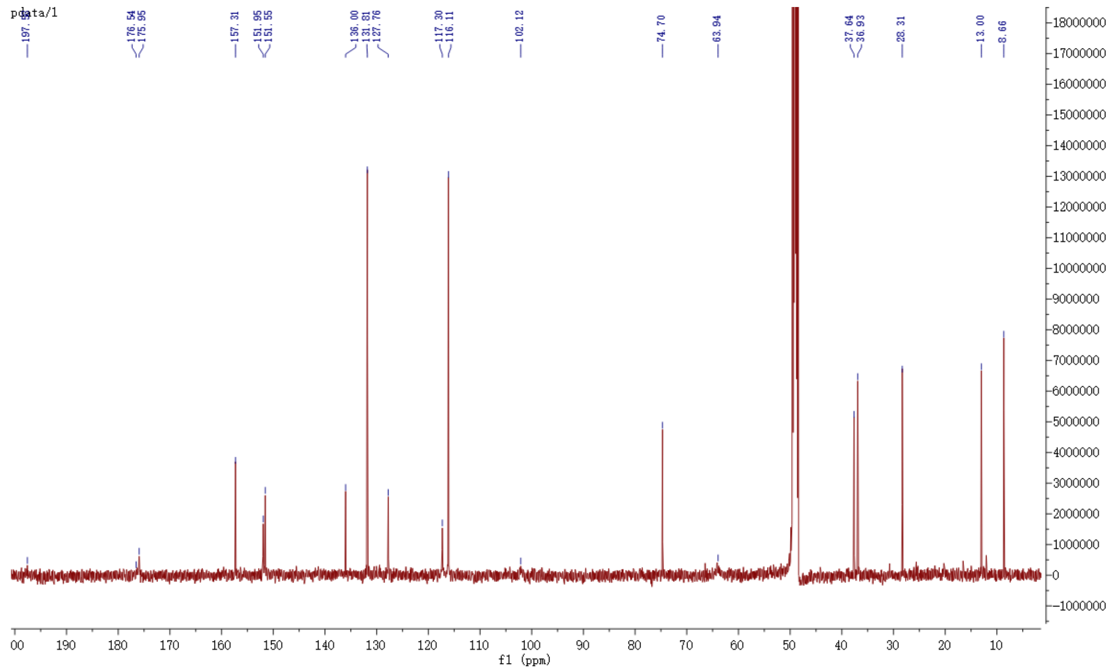

^{13}C NMR spectrum of 11-hydropretenellin A (**11**) (100 Hz, CD3OD-*d₄*).

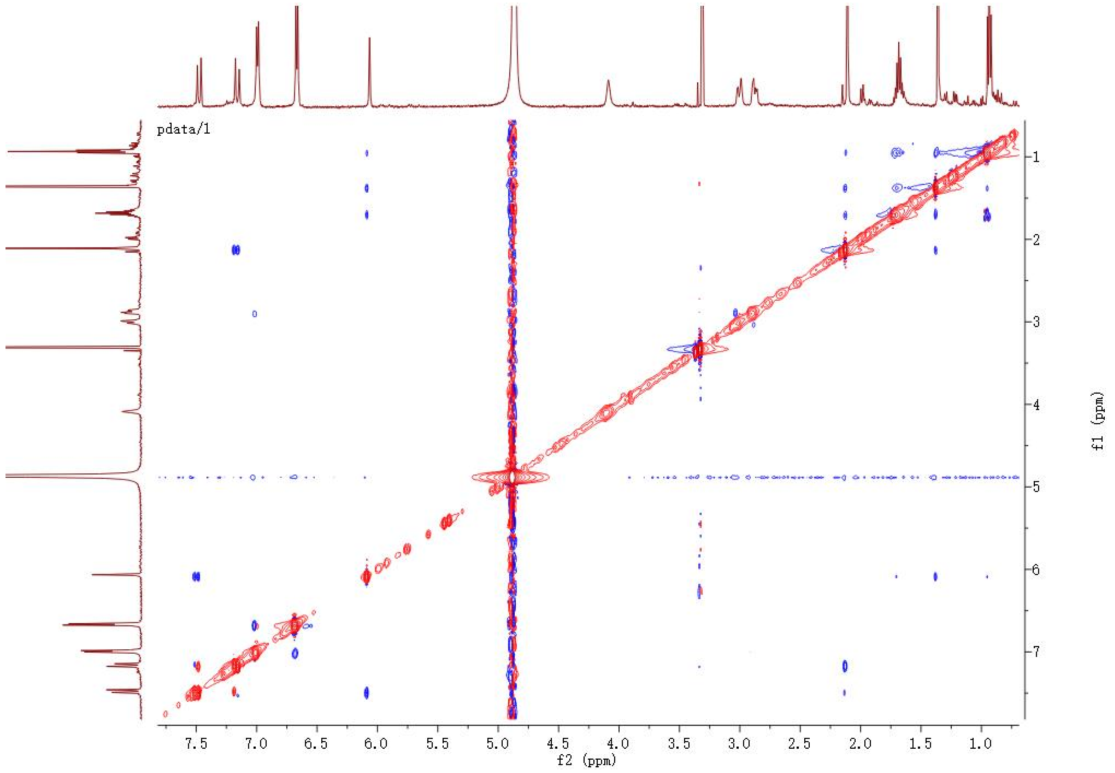

^{1}H-^{1}H NOESY spectrum of 11-hydropretenellin A (**11**) (500 Hz, CD3OD-*d₄*).

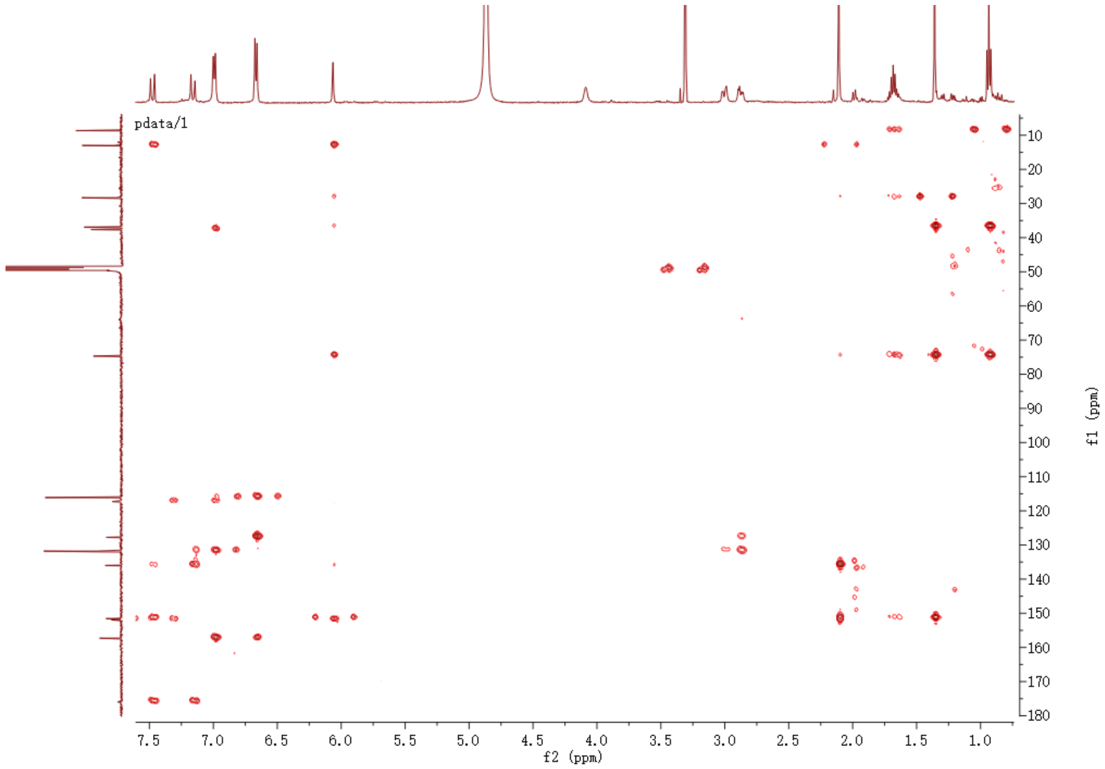

HMBC spectrum of 11-hydropretenellin A (**11**).

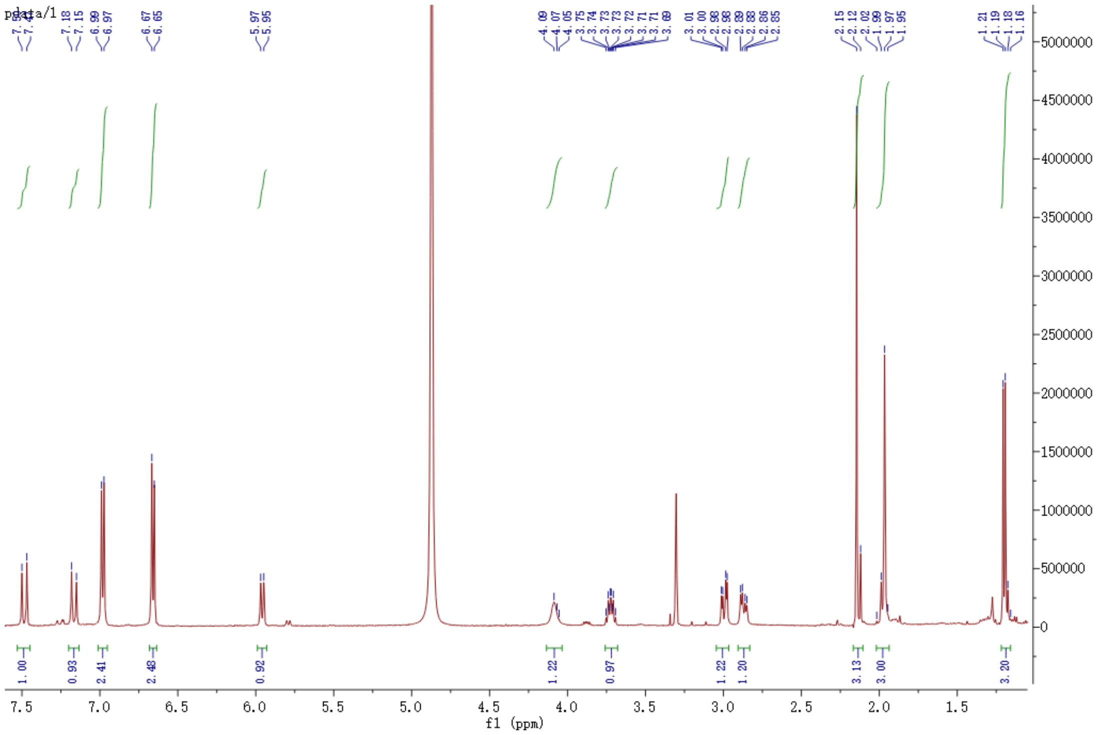

^{1}H NMR spectrum of 12-oxopretenellin A (**12**) (500 Hz, CD3OD-d_4).

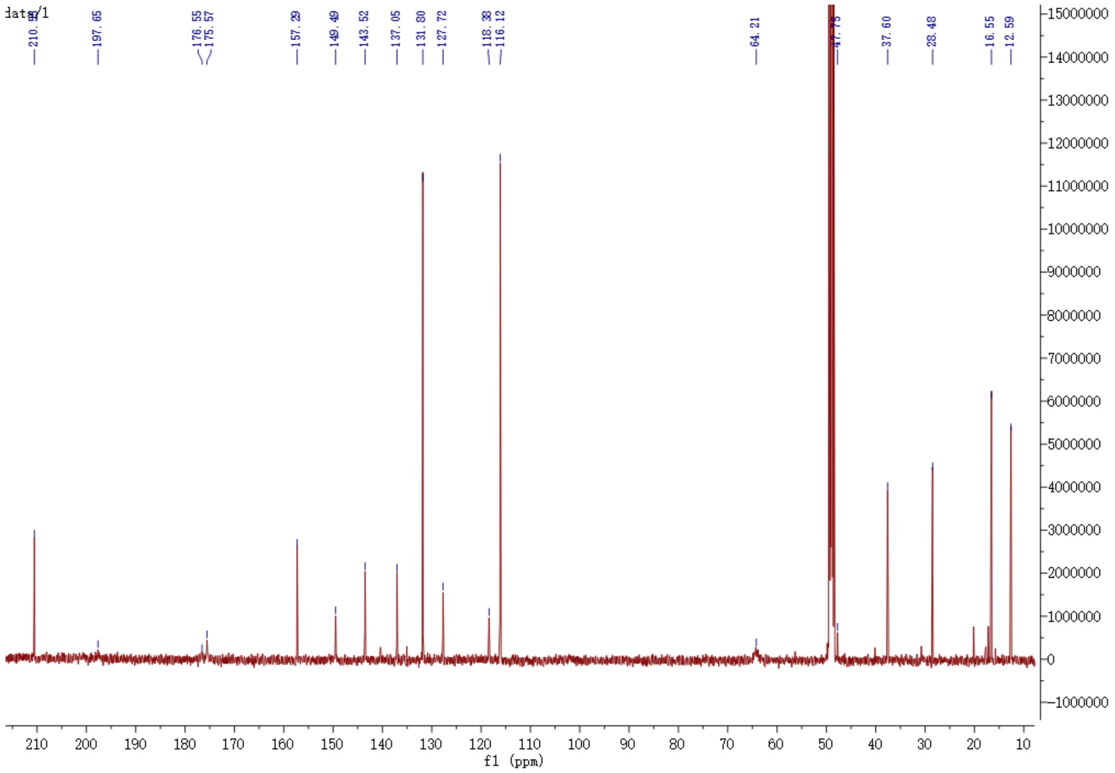

^{13}C NMR spectrum of 12-oxopretenellin A (**12**) (100 Hz, CD3OD-d_4).

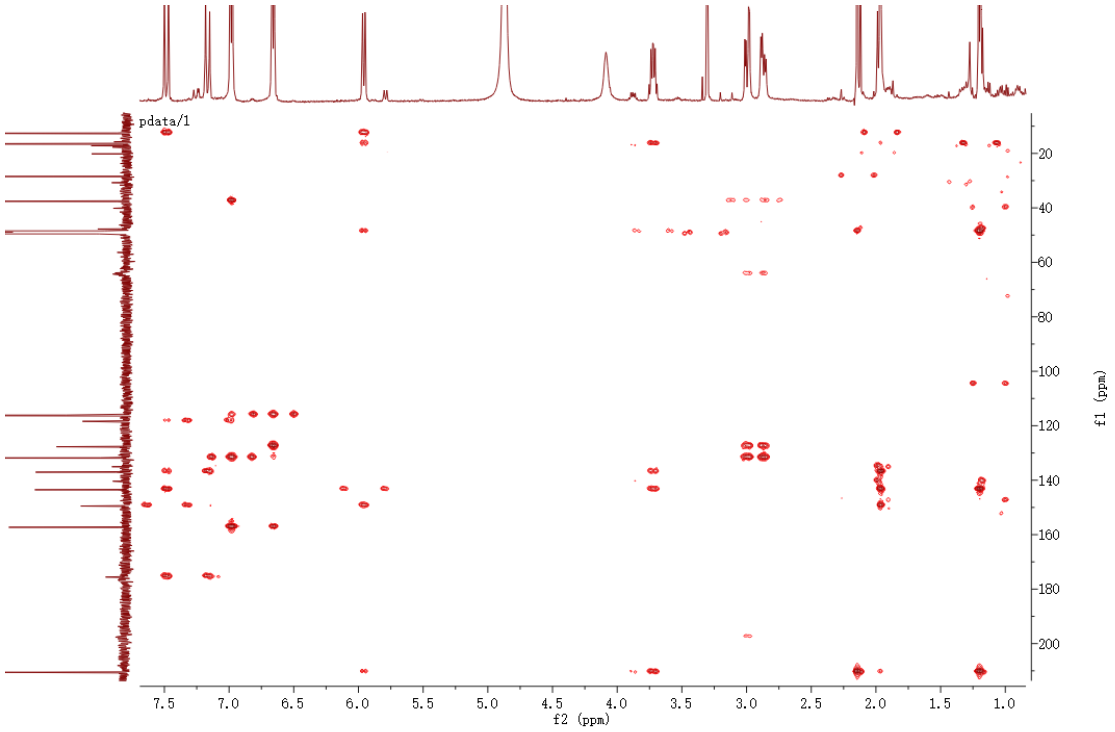

HMBC spectrum of 12-oxopretenellin A (**12**).

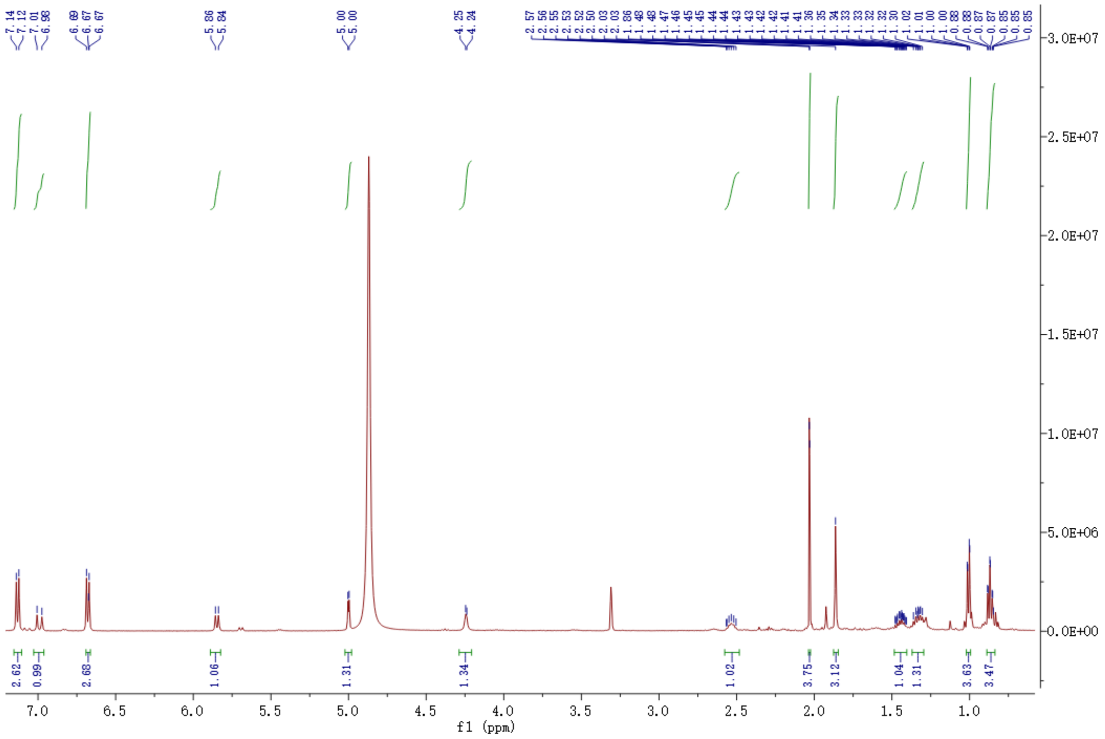

^{1}H NMR spectrum of prototenellin D (**13**) (500 Hz, CD3OD-d_4).

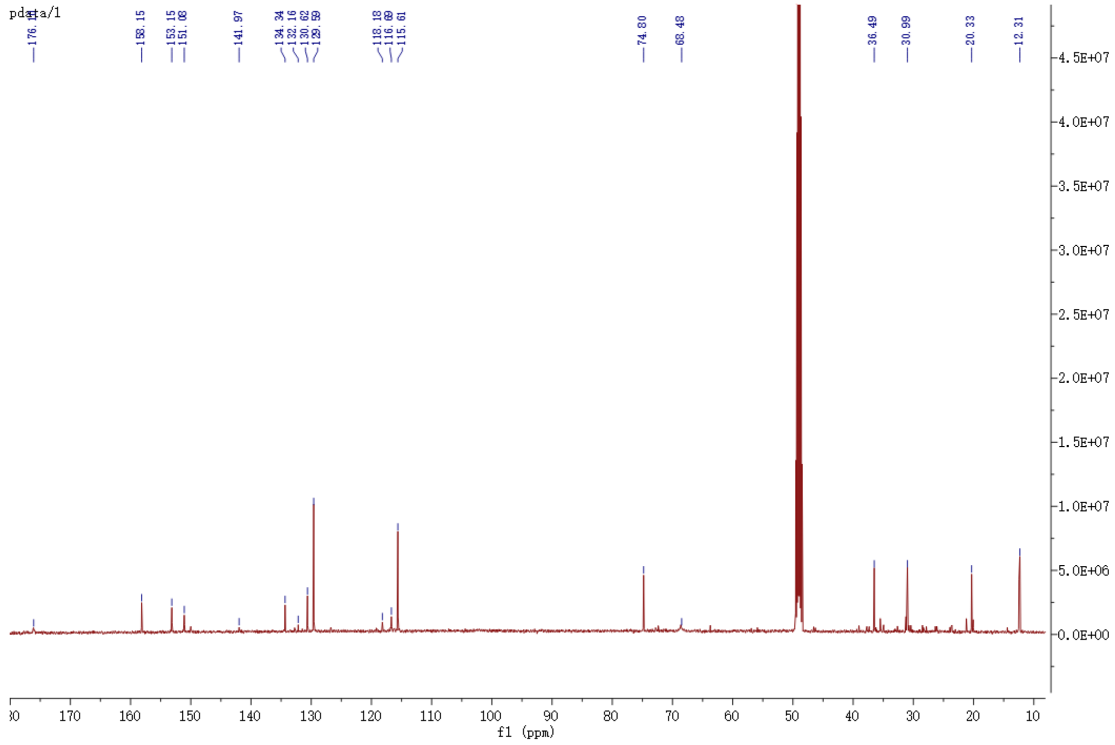

^{13}C NMR spectrum of prototenellin D (**13**) (100 Hz, CD3OD-d_4).

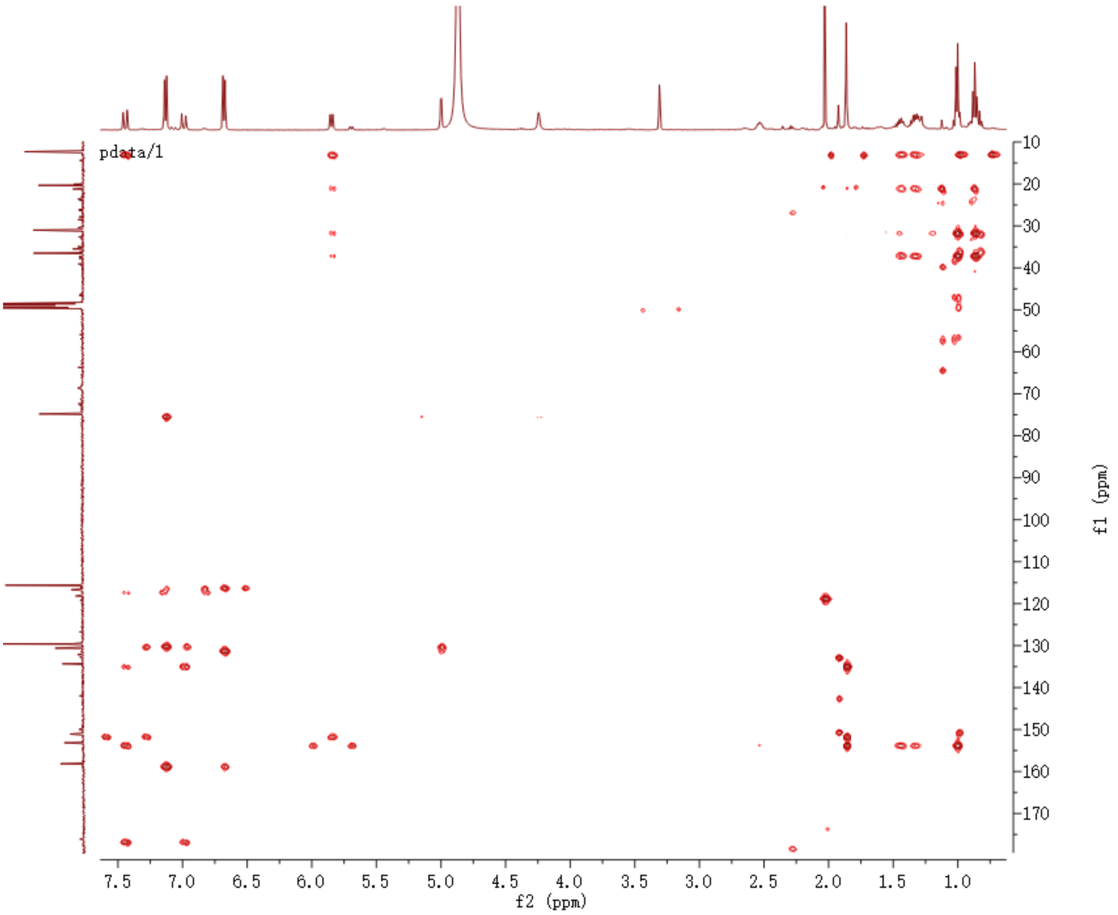

HMBC spectrum of prototenellin D (**13**).

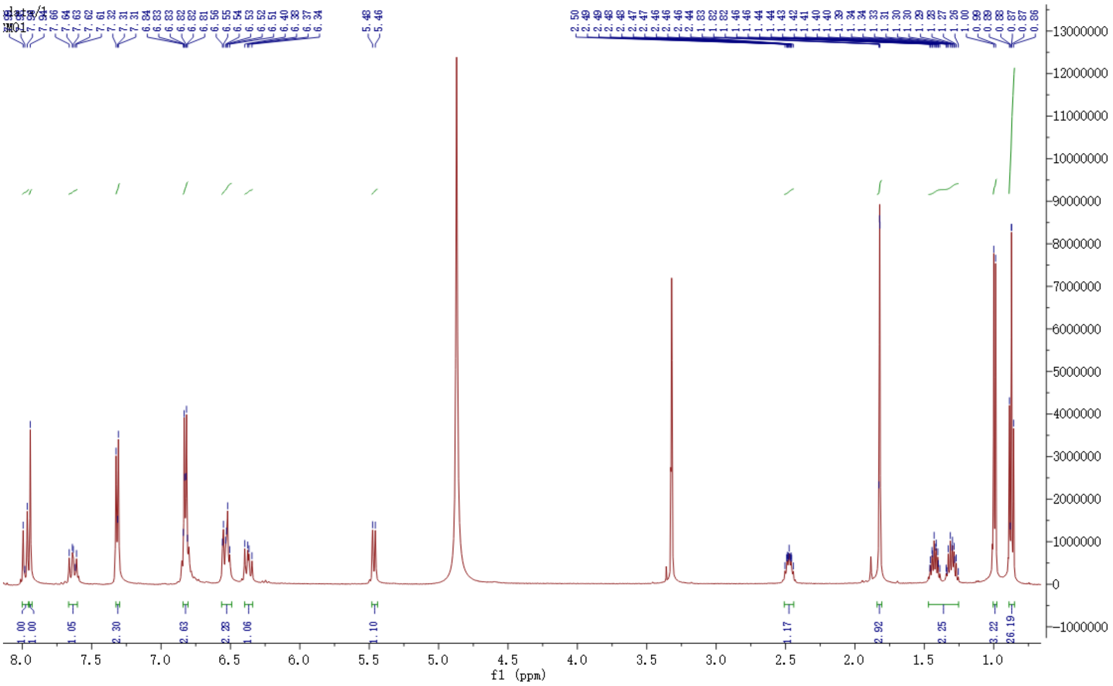

^{1}H NMR spectrum of **Farinosone B** (500 Hz, CD_3OD-d_4).

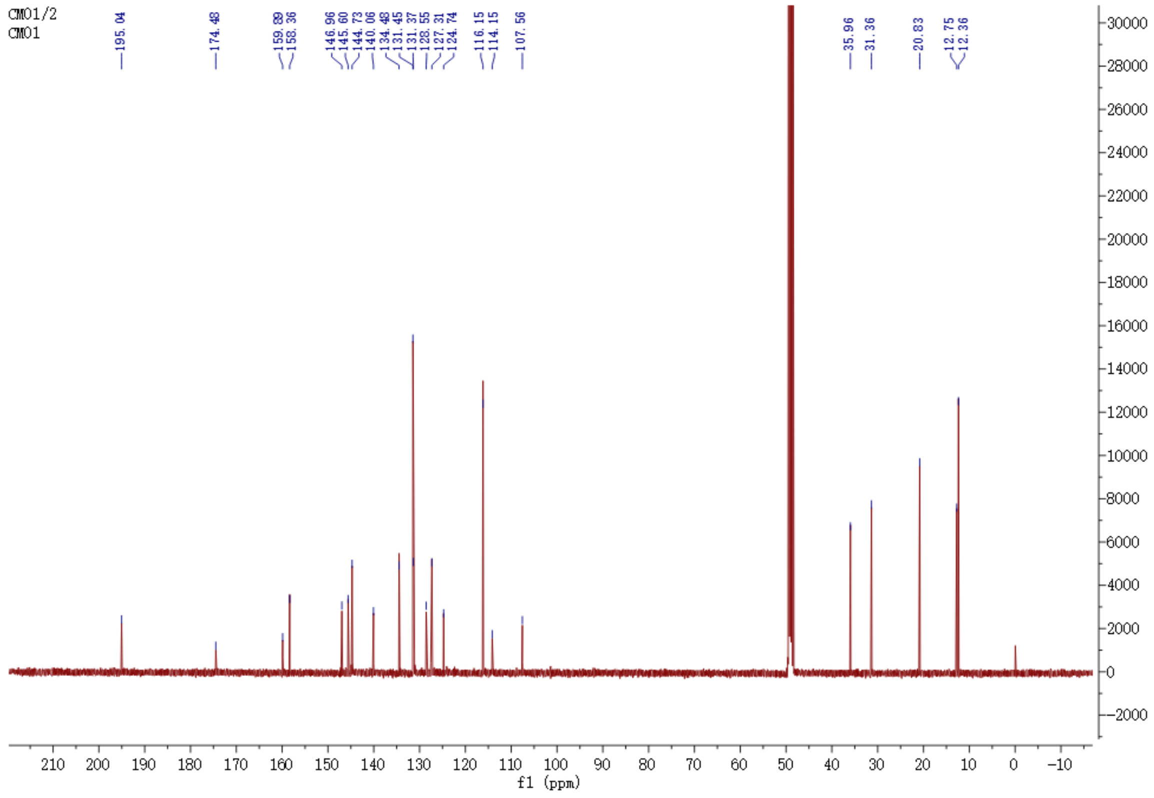

^{13}C NMR spectrum of **Farinosone B** (100 Hz, CD_3OD-d_4).

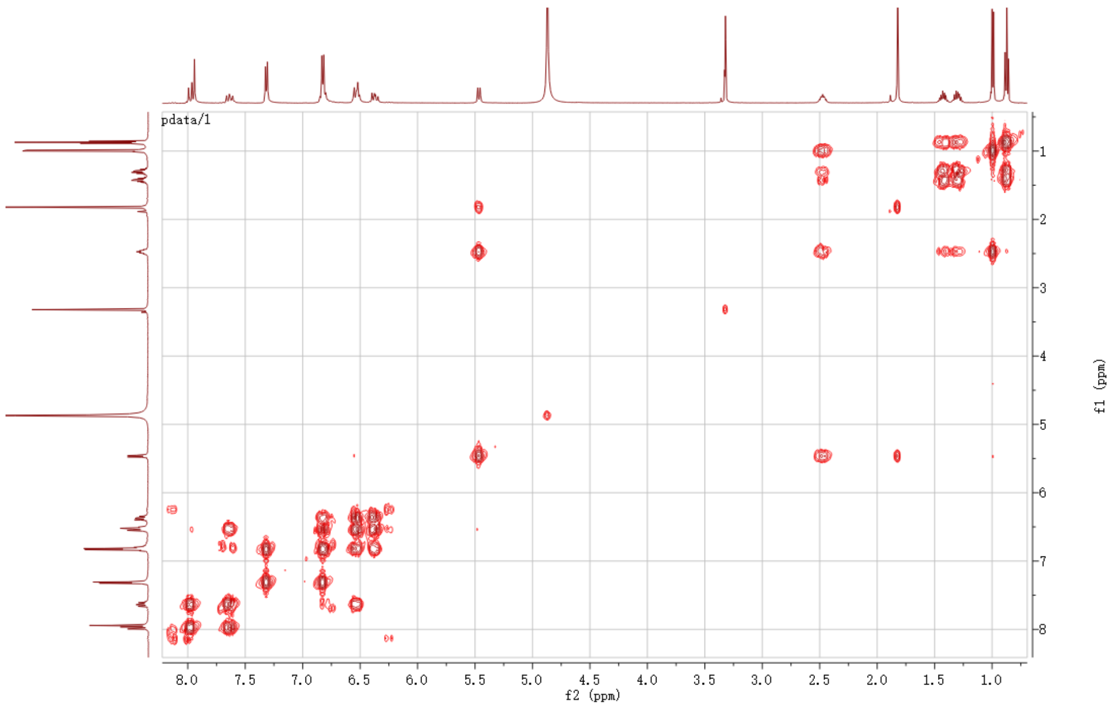

^{1}H-^{1}H COSY spectrum of **Farinosone B** (500 Hz, CD_3OD-d_4).

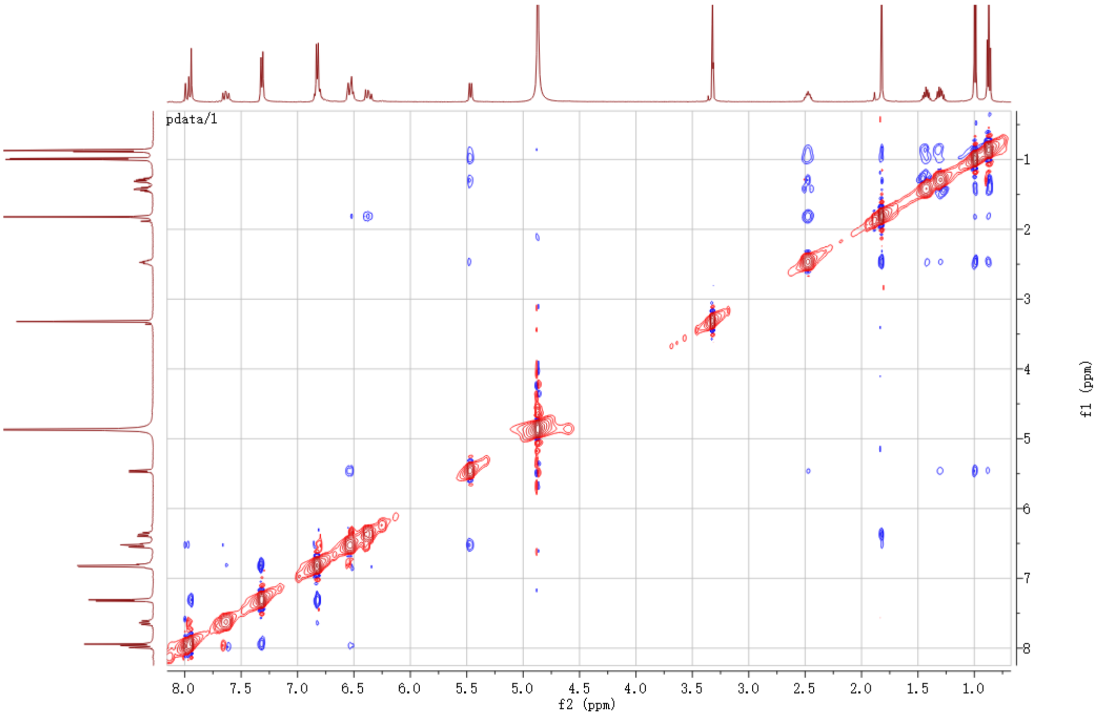

^{1}H-^{1}H NOESY spectrum of **Farinosone B** (500 Hz, CD_3OD-*d_4*).
