# Supplementary figures and images for: Inductive Production of the Iron-Chelating 2-Pyridones Benefits the Producing Fungus To Compete for Diverse Niches
Source: mBio. 2021 Dec 14;12(6):e03279-21. doi: 10.1128/mbio.03279-21 (PMC8669486; doi:10.1128/mbio.03279-21)

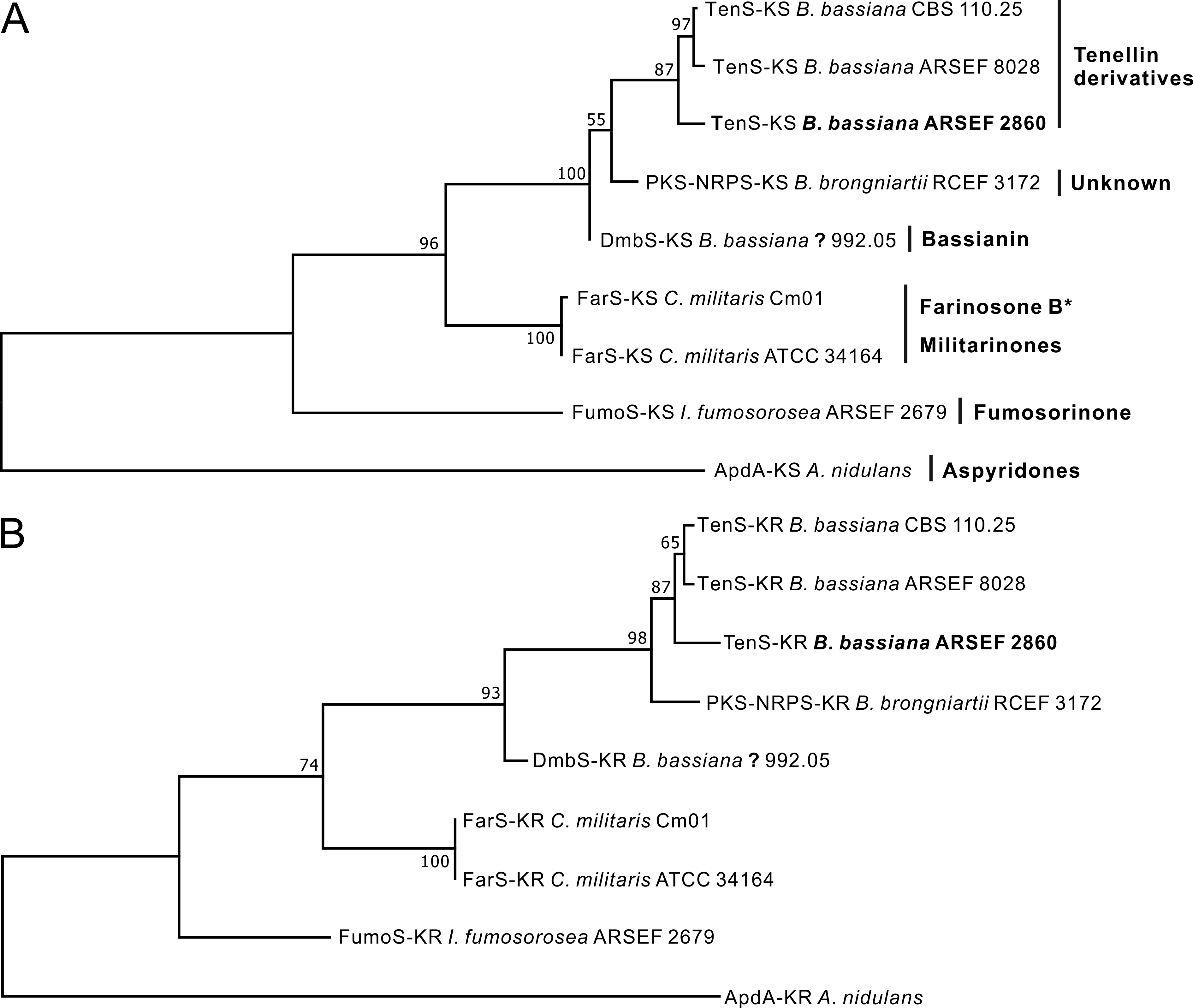

Supplement: FIG S2 [file mbio.03279-21-sf002.tif]

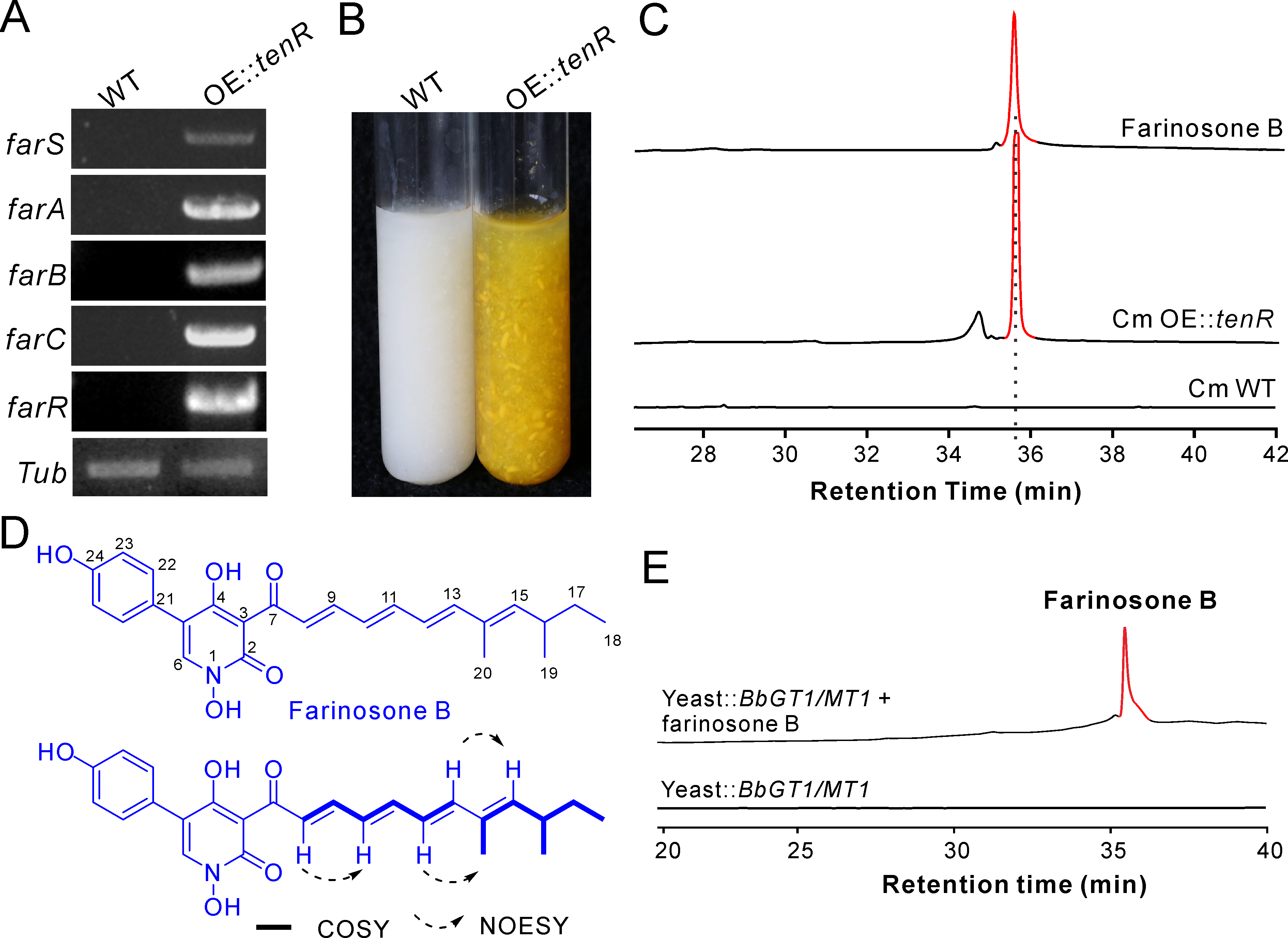

Supplement: FIG S3 [file mbio.03279-21-sf003.tif]

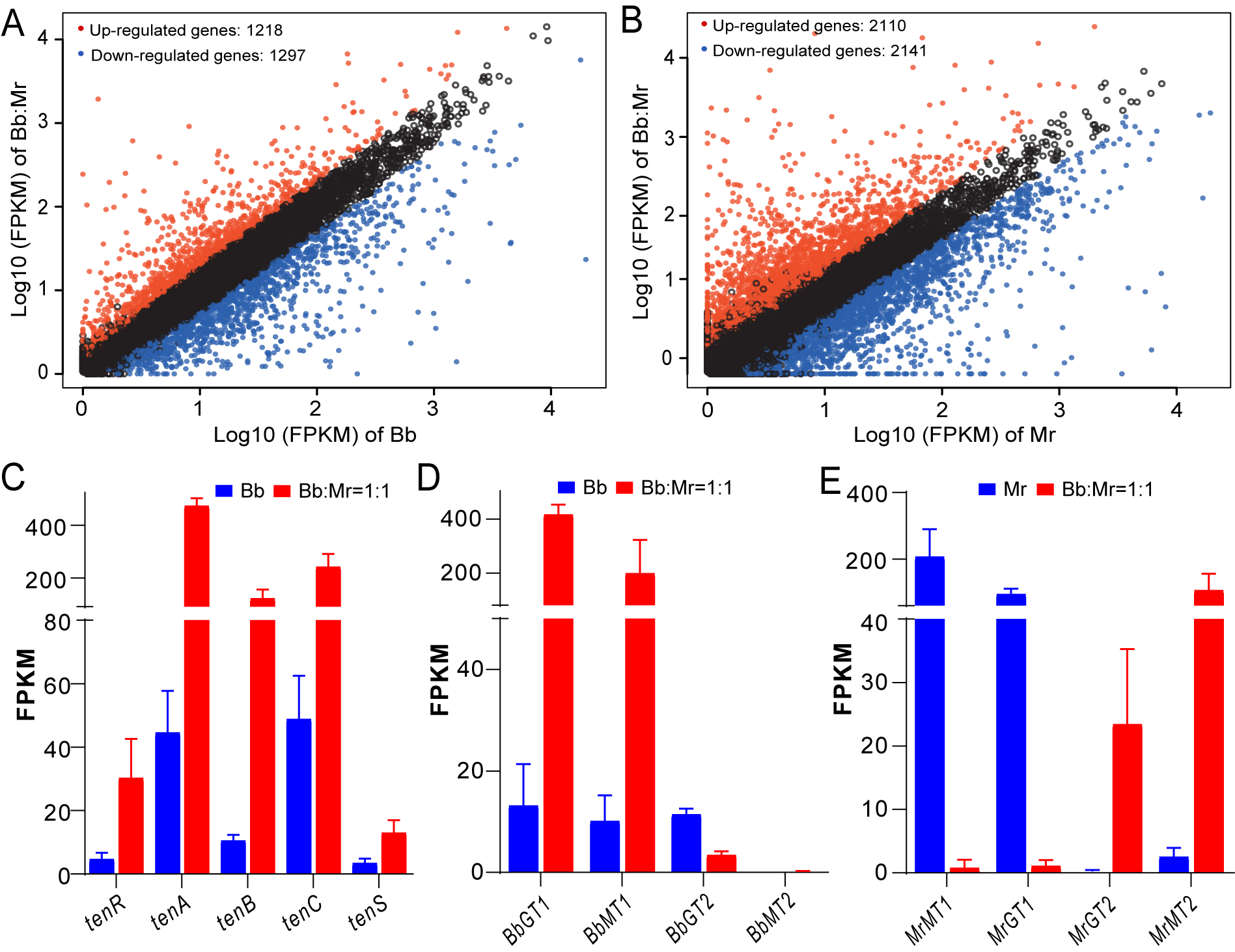

Supplement: FIG S4 [file mbio.03279-21-sf004.tif]

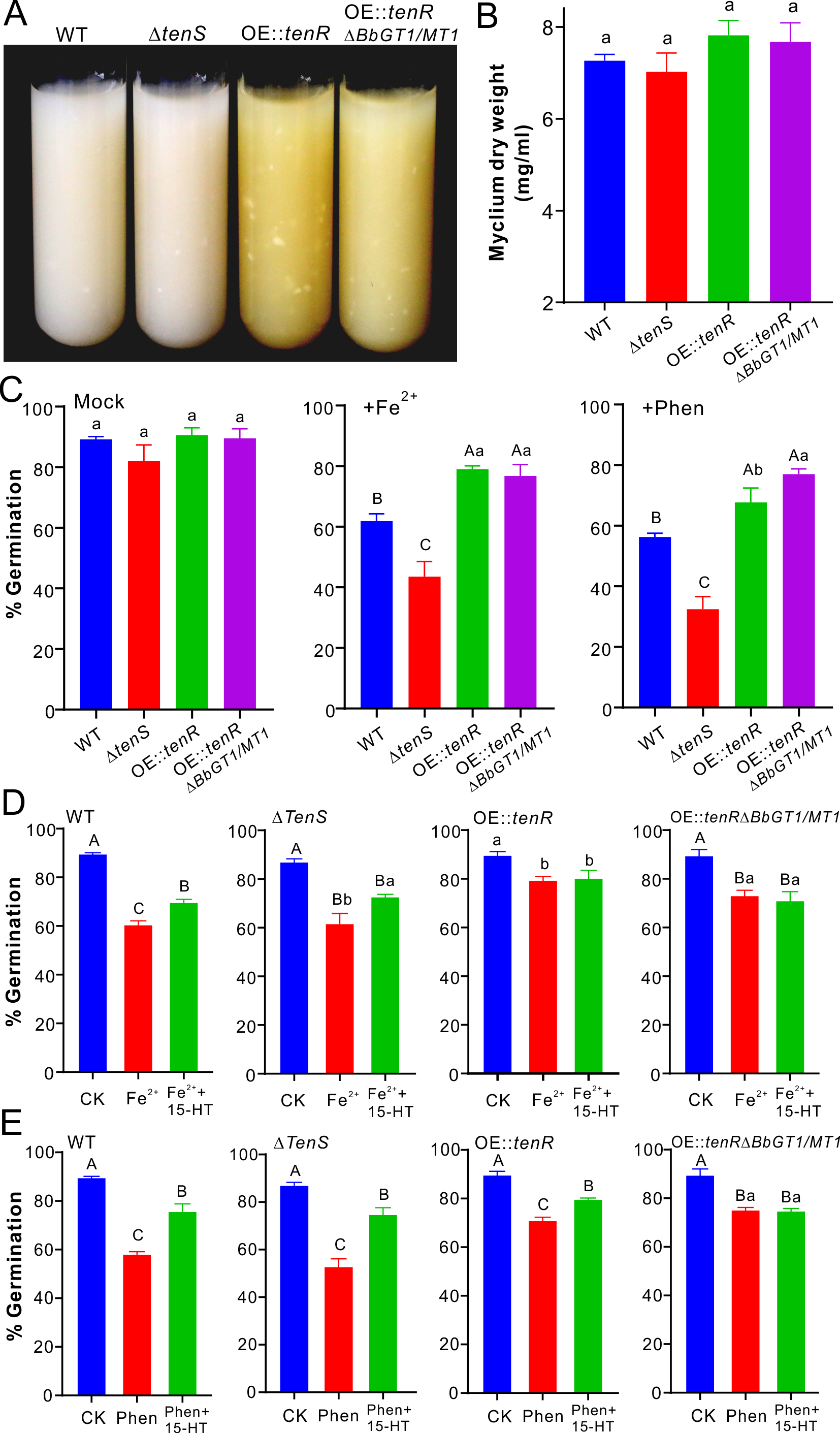

Supplement: FIG S5 [file mbio.03279-21-sf005.tif]

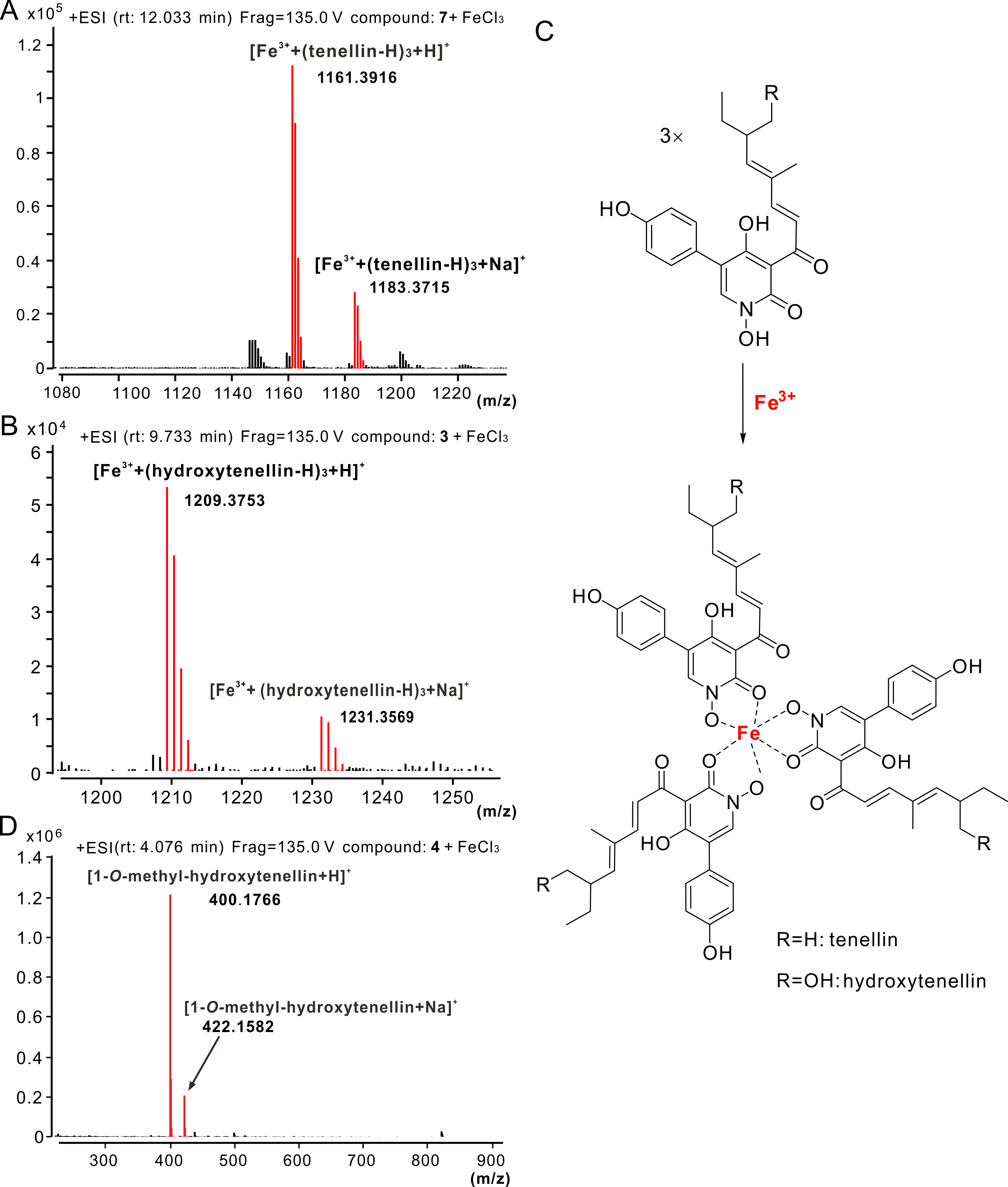

Supplement: FIG S6 [file mbio.03279-21-sf006.tif]
