# Supplementary material for: Inductive Production of the Iron-Chelating 2-Pyridones Benefits the Producing Fungus To Compete for Diverse Niches
Source: mBio. 2021 Dec 14;12(6):e03279-21. doi: 10.1128/mbio.03279-21 (PMC8669486; doi:10.1128/mbio.03279-21)
Supplement: TABLE S2 [file mbio.03279-21-st002.pdf]

TABLE S2 PCR primers used in this study.

**Mutants**	**Primers***	**Primer sequences**	***RE****	**Note**
Beauveria bassiana				
OE::*BBA_07334* (*tenR*)	Uf	TCAATAACAACTAGTTCTAGAATGGAGCCCGGCCCGGCG	*XbaI*	Overexpression of *tenR*
	Ur	CGCGGTGGCGGCCGCTCTAGATCACAGCGGCAGGCCT`AC		
OE::*BBA_07339*	Uf	TCAATAACAACTAGTTCTAGAATGGAAACAACGGTCAAGGC	*XbaI*	Overexpression of *BBA_07339*
	Ur	CGCGGTGGCGGCCGCTCTAGATCATTCGTCGATTGAAACAT		
Δ*tenS*	Uf	CTGCAGCCCGGGGGATCCTCTTATTTCTAGGGTAGGCG	*BamHI*	Gene deletion
	Ur	ATCTTCTGTCGACGGATCATTGGGACAAGGCGTTCA		
	Df	CGAGATCTGATGAAAAACGGCAGGTATTCCACT	*Spe1*	
	Dr	GGCCGCTCTAGAACTAGTTCTACGGTTCGCCACAAA		
	F	GGAGACAGTTCTGCCGACTT	/	Deletion mutant verification
	R	ATGCGATTGGAGATGATGC		
OE::*tenR*Δ*tenS*	Uf	CTGCAGCCCGGGGGATCCTCTTATTTCTAGGGTAGGCG	*BamHI*	Gene deletion
	Ur	ATCTTCTGTCGACGGATCATTGGGACAAGGCGTTCA		
	Df	CGAGATCTGATGAAAAACGGCAGGTATTCCACT	*Spe1*	
	Dr	GGCCGCTCTAGAACTAGTTCTACGGTTCGCCACAAA		
	F	GGAGACAGTTCTGCCGACTT	/	Deletion mutant verification
	R	ATGCGATTGGAGATGATGC		
OE::*tenR*Δ*tenA*	Uf	CTGCAGCCCGGGGGATCCTTTGTCAACTTTCGTAATCC	*BamHI*	Gene deletion
	Ur	ATCTTCTGTCGACGGATCGGTCTCAAGTCTGCCTCA		
	Df	CGAGATCTGATGAATCCAGTCGTCGCCATCAC	*Spe1*	
	Dr	GGCCGCTCTAGAACTAGTTAAACCAACGCCATTACC		
	F	TTTGCCGTTTGGTCCTCC	/	Deletion mutant verification
	R	CCTCCCATACCTTATCCTGT		
OE::*tenR*Δ*tenB*	Uf	CTGCAGCCCGGGGGATCCGAACGGCGAGTCACAACA	*BamHI*	Gene deletion
	Ur	ATCTTCTGTCGACGGATCCGGGAAATGGGAGGGTAG		
	Df	CGAGATCTGATGAAAGGCAACAAGCCGATTTA	*Spe1*	
	Dr	GGCCGCTCTAGAACTAGTCCAGCCAAGGTCGTAGTG		

	F	TGGAACGGCGAGTCACAA	/	Deletion mutant verification
	R	GAGACGCATCGCTTCACG		
OE*::tenRΔBbGT1*	Uf	CTGCAGCCCGGGGGATCCCCATTTGTTCCCGTAAGT	*BamHI*	Gene deletion
	Ur	ATCTTCTGTCGACGGATCTCAACCATTCTCGCTTTT		
	Df	CGAGATCTGATGAAGACCAACTTCTTGTCCCTG	*Spe1*	
	Dr	GGCCGCTCTAGAACTAGTACCTTTATCGCCTGCTGT		
	F	CTCGTCATCTAAACCTCG	/	Deletion mutant verification
	R	CATAGCCAGCGTTCAAAA		
OE::tenRΔBbMT1	Uf	CTGCAGCCCGGGGGATCC TGATTGAAGGGCGAGGAA	*BamHI*	Gene deletion
	Ur	ATCTTCTGTCGACGGATC GCATCTGCGGGTAGAACG		
	Df	CGAGATCTGATGAA TATCCAGTCACCTGCCCTAG	*Spe1*	
	Dr	GGCCGCTCTAGAACTAGT ACCGACCCTGAACGAAAT		
	F	TCGGTCTTCGGACATTTA	/	Deletion mutant verification
	R	CCATTTGTTCCCGTAAGT		
OE*::tenRΔBbGT1 /MT1*	Uf	CTGCAGCCCGGGGGATCCTTGTCTGCCACATTTCTG	*BamHI*	Gene deletion
	Ur	ATCTTCTGTCGACGGATCCGACCAACTTCTTGTCCCT		
	Df	CGAGATCTGATGAATGTTACGAGGTTTAGATGACG	*Spe1*	
	Dr	GGCCGCTCTAGAACTAGTGCAACTGCCTGGATACTTT		
	F	CGCCCTAGTAGATCAATCC	/	Deletion mutant verification
	R	CACCACTGGCTGAGTATGT		
OE::*tenRΔBbGT2/ MT2*	Uf	TCATCTTCTGTCGAC AACCCTCAATACACCAACCT	*PstI*	Gene deletion
	Ur	TTGATATCGAATTCC CAGAAGCCCTCTACACCC		
	Df	CCGCTCTAGAACTAG GAGCCCTACCAGCTATGA	*SpeI*	
	Dr	GTCACCGAGATCTGA GAAGGTCCTCAACTACTATG		
	F	ACTGGCATCTTCTTAGGGC	/	Deletion mutant verification
	R	GGACATTGTGGGAAACTCG		
tubulin	Tub-F	GAGATTGTGAGTGCTCCTACC	/	RT-PCR reference
	Tub-R	CCCTTAGCCCAGTTGTTA		
Metarhizium robertsii				
Δ*MrGT1*	Uf	GCTTGATATCGAATTCCTGCAGTCGCCTAAAGATGCTGGG	*PstI*	Gene deletion
	Ur	CAATATCATCTTCTGTCGACTGTTTGTAAACTGAGACGGCC		

	Df	GTCACCGAGATCTGATCCTTCCACTCATAACCTAGCTAG		
	Dr	AAGGGAACAAAAGCTGGAGGCAAGAAGGGTTTCC		
	F	TTCACGTAACTGGCAGGCAA	/	Deletion mutant verification
	R	ATCTCGCCACTTCGCTCTTC		
Δ*MrMT1*	Uf	GCTTGATATCGAATTCCTGCAGTCCTTCCACTCATAACCTAGCTAG	*BamHI*	Gene deletion
	Ur	TCATCTTCTGTCGACAGGCAAGAAGGGTTTCCAG		
	Df	ACCGAGATCTGATGAACTAGTTGAGATGGCTGCTCTCAGC	*XbaI*	
	Dr	AGGGAACAAAAGCTGACTAGTTGCAGATCTCGACCGTCAC		
	F	ACCCTTGAAGCGTCCATC	/	Deletion mutant verification
	R	AAGGGCGAAGTGTCTCC		
Saccharomyces cerevisiae				
BbGT1 (*Leu-*)	F	TGACGACAAGCTTCATATGTCTCCGTCACGCAGCGAG	*NdeI*	Plasmid modification by including the both auxotrophic marker genes
	R	TGGTGATGTCCGTTTAAACTCAATCCATAGCCGCCCACTTGAG	*PmeI*	
BbMT1 (*Trp-*)	F	TGACGACAAGCTTCATATGGCTCTCGTCGAAAAAATTCAGT	*NdeI*	
	R	TGGTGATGTCCGTTTAAACTCAGTTTCTTTTTGCGATCAACATGT	*PmeI*	

*, Uf and Ur represent the forward and reverse primers for amplification of the upstream (5'-flanking) region for each target gene; Df and Dr represent the forward and reverse primers for amplification of the downstream (3'-flanking) region for each target gene; **, RE, restriction enzymes.
